# Unraveling biomolecular and community grammars of RNA granules via machine learning

**DOI:** 10.1093/pnasnexus/pgaf093

**Published:** 2025-03-19

**Authors:** Zhan Ban, Yuchen Lin, Yan Yan, Kenneth A Dawson

**Affiliations:** Centre for BioNano Interactions, School of Chemistry, University College Dublin, Dublin 4, D04 N2E5, Ireland; School of Biomolecular and Biomedical Science, UCD Conway Institute of Biomolecular and Biomedical Research, University College Dublin, Dublin 4, D04 N2E5, Ireland; Centre for BioNano Interactions, School of Chemistry, University College Dublin, Dublin 4, D04 N2E5, Ireland; School of Biomolecular and Biomedical Science, UCD Conway Institute of Biomolecular and Biomedical Research, University College Dublin, Dublin 4, D04 N2E5, Ireland; Centre for BioNano Interactions, School of Chemistry, University College Dublin, Dublin 4, D04 N2E5, Ireland; School of Biomolecular and Biomedical Science, UCD Conway Institute of Biomolecular and Biomedical Research, University College Dublin, Dublin 4, D04 N2E5, Ireland; Centre for BioNano Interactions, School of Chemistry, University College Dublin, Dublin 4, D04 N2E5, Ireland; School of Biomolecular and Biomedical Science, UCD Conway Institute of Biomolecular and Biomedical Research, University College Dublin, Dublin 4, D04 N2E5, Ireland

**Keywords:** RNA granule proteome, machine learning, P-body, stress granule, protein-protein interaction network

## Abstract

Membraneless RNA granules are essential for posttranscriptional gene regulation, influencing cellular functions and contributing to neurodegenerative diseases. However, a comprehensive understanding of their compositions and organization has been challenging due to their complex nature. In this study, we develop robust machine learning models to reliably identify RNA granule proteomes within the human proteome, capturing central RNA granule characteristics despite the heterogeneity across diverse in vitro conditions. Furthermore, we uncover protein–protein interaction (PPI) community grammars within the RNA granule proteome, highlighting PPIs as key stabilizers of RNA granule structure and function. Dense PPI clusters serve as stable “cores,” forming key functional subunits across heterogeneous RNA granules. We introduce a state-of-the-art framework for understanding RNA granule biology and underscore the critical role of PPIs in maintaining RNA granule integrity.

**Significance Statement**

RNA granules, essential for posttranscriptional gene regulation, are implicated in neurodegenerative diseases, yet their complex composition and dynamic assembly remain poorly understood. This study leverages machine learning to predict RNA granule proteomes within the human proteome, revealing key biomolecular mechanisms that drive granule formation and identifying crucial protein–protein interactions (PPIs) that stabilize their structure. By uncovering dense PPI clusters that form stable “core” substructures across diverse in vitro conditions, we provide a novel framework for understanding RNA granule biology. This approach advances the field by offering new insights into granule organization and paving the way for therapeutic exploration of diseases linked to RNA granule dysfunction.

## Introduction

RNA granules are membraneless organelles composed primarily of RNA and RNA-binding proteins (RBPs), playing a pivotal role in posttranscriptional gene regulation, including RNA metabolism, transport, and translation (1). Dysregulation of RNA granules has been implicated in various neurodegenerative diseases, such as frontotemporal lobar degeneration and amyotrophic lateral sclerosis (2, 3). Despite their biological significance, the comprehensive identification and characterization of RNA granule components and their organization remain challenging due to the inherent heterogeneity and dynamic nature of these membraneless structures. Consequently, there is a lack of frameworks for providing systematic, a priori guidance for research on RNA granule formation and functionality.

Traditional approaches for studying RNA granules have relied heavily on methods like co-localization with known markers and catalog construction (4), which are often time-consuming and limited in scope. These methods also fail to provide a holistic understanding of the molecular mechanisms driving RNA granule formation and function. Advanced methods, like an engineered ascorbate peroxidase (APEX)-based proximity labeling technique, have emerged to map protein components and their interactions within RNA granules, revealing previously unknown components and providing insights into their dynamic assembly (5–7). However, these techniques also have limitations; they struggle to offer a comprehensive view of the RNA granule proteome due to the high heterogeneity arising from the complex and context-dependent nature of these granules under diverse in vitro conditions (5). Recently, RNA granule studies have increasingly utilized machine learning models to understand liquid–liquid phase separation (LLPS), a key driving force in RNA granule formation. Although LLPS models (8–10) have significantly advanced our understanding of the biophysical processes (i.e. LLPS) involved in granule formation, they fail to capture the full complexity of RNA granules. For instance, these models may overlook the role of RNA–protein interactions through RNA-binding domains (11) and the critical regulatory functions of RBPs in granule formation and functionality. In addition to the component studies, recent research, such as those by Jain et al. (12) and Markmiller et al. (5), shed light on relatively stable “core” substructures and indeed dense protein–protein interaction (PPI) networks within stress granules (SGs). However, without a fundamental approach to predict RNA granules, comprehensive perspectives on the highly dynamic, stress-dependent, and heterogeneous RNA granule systems remain limited.

In response to this gap, we developed a machine learning–based approach that integrates a diverse set of sequence-based protein features to accurately predict the RNA granule proteome within the human proteome. Despite the limited availability of high-confidence RNA granule proteins (e.g. 280 tier 1 proteins for SGs) in existing databases (4), our models demonstrate robust performance, enabling the identification of critical RNA granule components across a wide range of heterogeneous experimental conditions. The predicted proteome is significantly enriched in biological functions that are critical to key activities of RNA granules, such as RNA transport (13), RNA editing (14) and RNA decay (14), and key protein domains. In this study, we address the limitations of previous LLPS methods by incorporating RNA-binding domain information with RBP-specific features, identifying essential RBP components, and capturing the critical biological functions of RNA granules beyond simple phase separation properties. To further elucidate the molecular underpinnings of RNA granules, we construct a PPI network using the predicted RNA granule proteome. By applying graph theory and analyzing biomolecular propensities, we uncover community grammars that govern RNA granule formation and function. Our analysis identifies central components within the dense PPI network that are crucial for maintaining the stability and functionality of RNA granules. Additionally, we discover three key PPI clusters characterized by dense interactions and high predictive propensities. These clusters, conserved across various RNA granules and stress conditions, likely represent stable “core” substructures that serve as functional subunits within the granules.

In summary, this study introduces a novel, machine learning–driven framework for predicting RNA granule proteomes and understanding the complex molecular and community-level interactions that drive RNA granule formation and functionality. Our findings underscore the critical role of PPIs in these processes and provide a comprehensive perspective on RNA granule biology, setting the stage for future experimental validation and exploration of RNA granules in diverse biological contexts.

## Results

### Specific features of RNA granule proteins

#### Learning set construction and protein feature selection

**Fig. 1.**
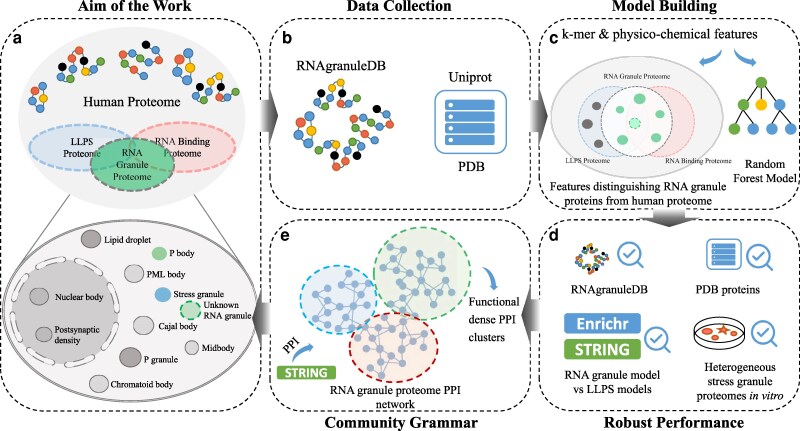
Schematic of the workflow of our work to reveal biomolecular and community grammars via machine learning models. a) To identify the RNA granule proteome from human proteome, the workflow comprises four critical stages. b) Data collection from RNAgranuleDB (4), UniProt, and PDB. c) Model building, where *k*-mer and physicochemical features are employed to characterize the specific attributes of RNA granule proteomes. d) Robust performance evaluation, utilizing diverse datasets including RNAgranuleDB, PDB proteins, in vitro SG proteomes, and predicted LLPS proteomes. e) Revealing community grammars (e.g. functional dense PPI cluster identification), facilitating the characterization of RNA granule proteome.

For the assessment of protein sequences for their potential to form RNA granules (i.e. SG, P-body [PB], and PB or SG [PBSG]), we utilize an essential RNA granule database, the RNAgranuleDB (4), that includes human SG and PB proteins as the positive learning set in our models (see Table S1). Negative protein candidate databases were randomly generated from the human proteome. In our analysis, we develop binary classification models using high-confidence RNA granule proteins with strong experimental evidence (4). These include tier 1 proteins of SG (280 tier 1 SG proteins + 280 negative proteins) and PBSG (473 tier 1 PBSG proteins + 473 negative proteins), or a combination of tier 1 and tier 2 proteins of PB (198 tiers 1 and 2 PB proteins + 198 negative proteins). Building upon prior machine learning models addressing protein condensate propensity (8) and PPI models (15, 16), this study uses physicochemical features (*n* = 19, e.g. length, isoelectric point, gravy values, and low complexity region [LCR] fraction), amino acid (aa) compositions, local sequence (*k*-mer content) features to capture the characteristics of RNA granule proteins (Fig. 1). The distinct distributions of selected fundamental physicochemical properties are evident between observed RNA granule protein candidates and the human proteome (excluding the collected RNA granule protein candidates), as shown in Fig. S1. The result indicates the high-confidence RNA granule proteins tend to be more hydrophobic (i.e. less gravy values), larger (i.e. higher molecular weights), and more disordered (i.e. with more LCR fractions) compared with the human proteome (*P* < 0.001).

#### *K*-mer features with characteristics of RNA granule proteins

*K*-mers, substrings of length *k* within biological sequences, are commonly used in sequence analysis (e.g. the evaluation of RNA–protein interactions (17, 18)). In this study, we extract distinct and abundant (the *k*-mers with the highest abundance among RNA granule protein candidates) 2-mer (*n* = 50) and 3-mer (*n* = 50) features with fractions of each *k*-mer to characterize each protein. To further evaluate the specific biomolecular grammars of RNA granules, we compare their *k*-mer features with those of the human proteome (Fig. S2) and the collected RBP proteome (Figs. S2 and S3 and Table S1). We observe a statistically significant enrichment of the specific RGG/RG motif, including arginine–glycine (RG), arginine–glycine–glycine (RGG), and glycine–arginine–glycine (GRG) *k*-mers, in tier 1 RNA granule protein candidates compared with both the human proteome and the RBP proteome from the RBPbase dataset. Notably, the enriched contents of RGG/RG motifs, predominantly located in intrinsically disordered regions (IDRs) of proteins, such as FUS, are known to play a vital role in the LLPS processes (19) of cells. This observation illuminates the potential of our feature representation methods in machine learning to capture relevant RNA granule formation at the molecular level (Fig. S4). However, we did not observe a significantly different distribution of *k*-mers associated with RGG/RG motifs across overall RNA granule protein candidates (tier 1 to tier 4) compared with the collected RBP proteome (Fig. S3). This suggests that high-confidence RNA granule proteins (tier 1), often found in the cores of RNA granules, may exhibit unique characteristics when compared with other RNA granule components (Fig. S4), consistent with the observed significance of stable “cores” in SGs' formation and functionality (12).

### Robust models distinguishing RNA granule proteome from human proteome

#### Robustness evaluation

**Fig. 2.**
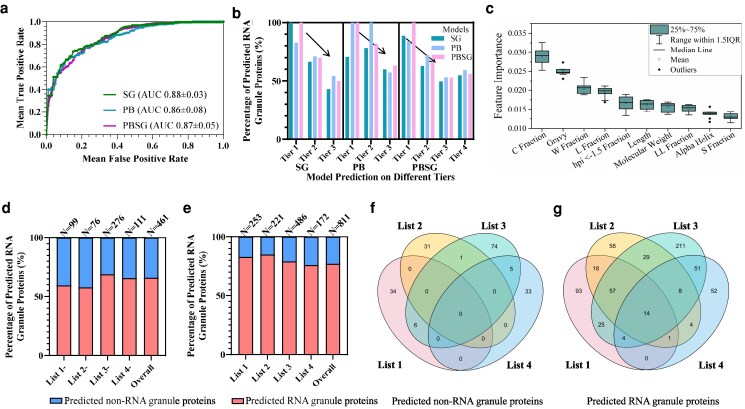
Robustness analysis of RNA granule protein models. a) The analysis applied the 10-fold cross-validation method, to estimate the model prediction performance (i.e. SG, PB, and PBSG models), measured by the AUC values (shown in mean± SEM, *n* = 10), respectively. b) We employed SG, PB, and PBSG models to predict observed RNA granule proteins with varying tiers in the RNAgranuleDB, respectively. c) The average Gini feature importance of 10-fold validated models (*n* = 10) was applied to screen top 10 most important features in the selected PBSG models (top features of SG and PB models in Fig. S8). We performed our RNA granule models to predict the experimental SG proteomes (*n =* 253 in list 1 (22), *n =* 221 in list 2 (5), *n =* 486 in list 3 (12), and *n =* 172 in list 4 (23)), excluded the training set proteins (d) and with overall proteins (e), respectively. Venn plots show the distribution of predicted non-RNA granule proteins (f) and predicted RNA granule proteins (g), among the four experimental SG proteomes, respectively. HPI, hydrophobicity or hydrophilicity values; C, cysteine; W, tryptophan; L, leucine; LL, leucine-leucine; S, serine.

With the selected protein features, we construct our RNA granule model to distinguish RNA granule proteins from the human proteome with the random forest algorithm (20), inspired by previous LLPS models (8, 9). Initially, we evaluate the robustness of the constructed RNA granule models with the 10-fold cross-validation method (21), as shown in Figs. 2 and S5–S7. Our RNA granule models could achieve robust and superior predictive performance (with areas under the receiver operating characteristic curve (AUCs) up to 0.88 ± 0.03; with area under precision-recall curve (PR AUC) up to 0.87 ± 0.05, as shown in Figs. S5 and S6). These results surpass classic LLPS protein prediction models, such as PSAP (AUC 0.89, PR AUC 0.25) and PScore (AUC 0.84, PR AUC 0.11), particularly in precision-recall performance (8). Furthermore, we proceed to predict proteins with varying tiers from the RNAgranuleDB, as illustrated in Fig. 2b. Notably, the identification percentages of different protein tiers are consistent with the evidence confidence (e.g. from 100% of tier 1 to 56% of tier 4 PBSG proteins). Moreover, the observed tendency aligns with six existing related models (i.e. PLAAC (24), Cat Granule (25), PScore (26), DDX4-like (27), R + Y (28), and LARKS (29)) on identifying different tier proteins as LLPS-prone proteins, but with much higher identified percentages (e.g. 42.9–100% by our models versus up to 16% (30) by the six related models) in the RNAgranuleDB. Therefore, our proteome-wide RNA granule models are capable of achieving superior and more reliable performance in predicting RNA granule protein compositions, specifically, compared with related, more general LLPS models.

Using robust RNA granule models, we identify cysteine residue content and gravy value as the top two important protein features affecting RNA granule protein classifications (Figs. 2c and S8–S10). Both show a negative correlation with RNA granule protein prediction propensities (Figs. S9 and S10), suggesting that higher cysteine content and increased hydrophilicity reduce the likelihood of a protein being classified as an RNA granule component. Specifically, cysteine residues, known to stabilize protein structures through disulfide bond formation, can inhibit LLPS by affecting protein folding (31). Therefore, the top two important protein features highlight the significance of LCRs with hydrophobicity (9, 32) in RNA granule formation. In addition, our RNA granule models exhibit the capability to identify crucial domains (such as RNA recognition motif 2 [RRM2] in hnRNPA2B1 and helicase ATP binding in DDX6) and regions (comprising all IDRs in hnRNPA2B1, DDX6, and DCP2) in Figs. S11 and S12, playing pivotal roles in RNA granule formation (30, 33, 34). Furthermore, we observe that probability peaks tend to manifest alongside low gravy values and a low fraction of cysteine residues. This trend also underscores the predominance of hydrophobicity and IDRs in RNA granule formation.

#### Robust model performance on heterogeneous experimental SG proteomes

In addition, we assess the model's accuracy using four important SG proteomes (list 1 (22), list 2 (5), list 3 (12), and list 4 (23)), as depicted in Fig. 2d–g. Our RNA granule models effectively identify ∼77% of the proteins across different SG proteomes, despite the heterogeneity in identification methods (e.g. proteomic analysis (12) and proximity mapping (22)), experimental conditions (e.g. 30 °C (12) and 37 °C (22)) and diverse stresses (e.g. heat shock stress (12) and arsenite stress (22)). Notably, only 14 out of 811 proteins (∼2%) are shared across all four proteomes, highlighting the strong context-dependence of SG compositions (5). Furthermore, as shown in Figs. 2f–g and S13, the accurately identified SG components (77%) by our models tend to be popular and center components among heterogeneous SG proteomes, while the unidentified SG components (23%) by our models mainly come from the specific and off-center components among heterogenous SG proteomes. It indicates that our RNA granule model holds promising potential to offer a holistic perspective for studying the general RNA granule components, potentially revealing core functional or structural features conserved across stress conditions.

### RNA granule proteome versus LLPS proteome

**Fig. 3.**
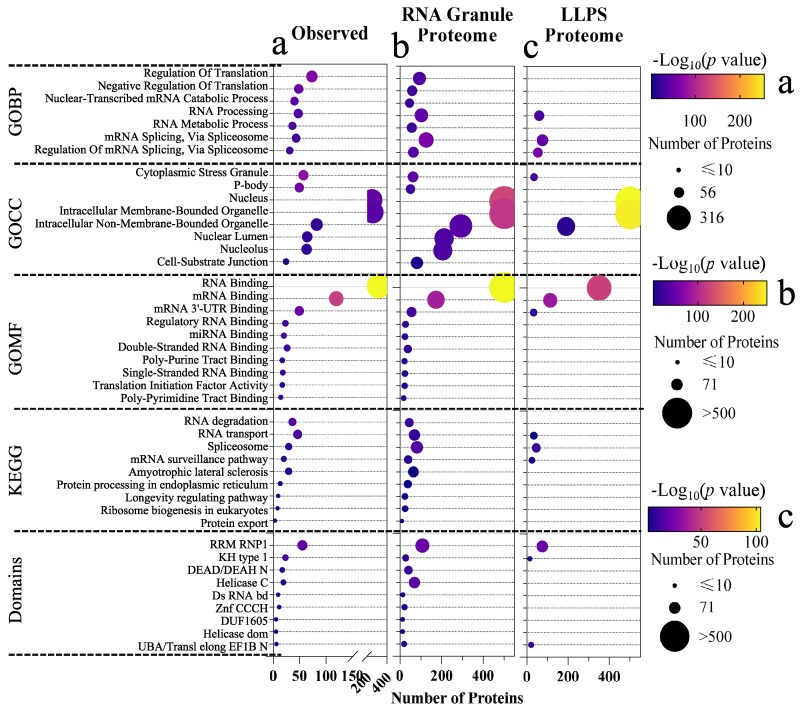
Functional enrichment analyses on the observed RNA granule proteins (a, *n* = 473), the identified high-confidence RNA granule proteome (b, *n* = 2,225), and the predicted LLPS proteome (c, *n* = 2,225). a) We collected observed high-confidence RNA granule proteins used to train our models (i.e. tier 1 PBSG proteins, *n* = 473). b) We selected the predicted high-confidence RNA granule protein candidates (with prediction probabilities over 0.7, *n* = 2,225) by the selected PBSG model. Then, we completed the functional enrichment analysis on GO biological process (GOBP), GO cellular component (GOCC), GO molecular function (GOMF), Kyoto Encyclopedia of Genes and Genomes (KEGG), and domains of the group of proteins. We show the shared terms of top 20 significantly enriched terms (according to their *P*-values) by the observed (*n* = 473) and predicted high-confidence RNA granule proteins (*n* = 2,225). c) We utilized three classic LLPS prediction models (PSAR, DeePhase, and PScore) to identify high-confidence LLPS-prone proteins (*n* = 2,225) according to the average rank percentile values of prediction LLPS scores. We applied the Enrichr platform to achieve the enrichment analysis.

Having established the comprehensive RNA granule proteome, we identify similar physicochemical patterns in high-confidence RNA granule protein candidates and the RNA granule proteome, in contrast to unlikely LLPS Protein Data Bank (PDB) proteins (Figs. S14–S16). Thus, highlighting the reliability of our machine learning models in capturing key determinants of RNA granule formation, like hydrophobicity across varying prediction probabilities (Fig. S16). In this study, we compare the enriched biological functions and domains of the identified RNA granule proteome with the observed RNA granule proteins and the LLPS proteome predicted by classic LLPS models (8, 9, 26) in Figs. 3, S17, and S18. We find a similar tendency between predicted LLPS scores and RNA granule propensities in Fig. S17. Interestingly, RNA granule proteins with higher predicted propensities exhibit higher likelihood as LLPS proteins and RBPs (Figs. S17 and S19), respectively, highlighting the close association of RNA granule proteins with phase separation and RNA binding.

As anticipated, the analysis (Figs. 3 and S18) confirms consistent enrichment of observed RNA granule proteins with those in our predicted RNA granule proteome, particularly for high-confidence predictions (probabilities ≥0.7), in biological processes, such as RNA transport (13), RNA editing (14), and nonmembrane organelle assembly. Notably, our RNA granule proteome shows significant enrichment of pivotal RNA-binding domains, such as RRM RNP1 (35), critical for RNA granule function. Additionally, the distinct differences between the predicted LLPS proteome and observed RNA granule proteins in the pivotal functional terms and domains (Figs. 3c and a and S18c and a) highlight the limitations of general LLPS models in identifying specific RNA granule proteins. These findings further validate the reliability of our machine learning models in identifying RNA granule proteomes from human proteomes by capturing RNA granule dynamics and critical RNA granule functions in cells.

### Community grammars in the identified RNA granule proteome

#### Central RNA granule proteins with high propensities

**Fig. 4.**
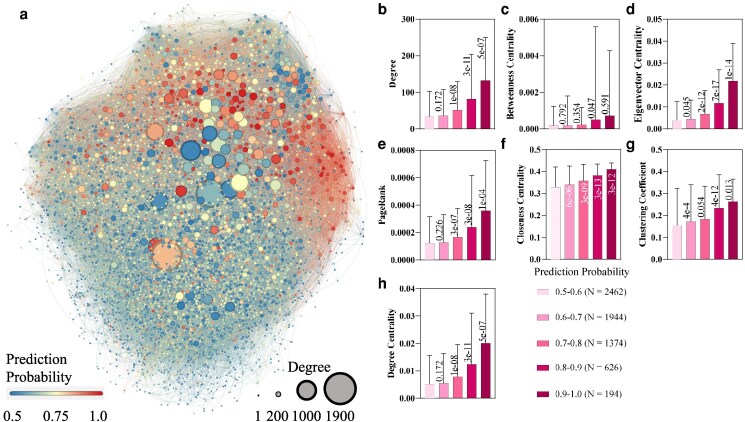
The centrality of identified high-confidence RNA granule proteins in RNA granule community. a) The network visualizes PPIs among the identified RNA granule proteome (*n* = 6,600). Nodes represent proteins, and edges represent interactions between them. Degree represents the number of interactions of each protein with other identified RNA granule proteins. b–h) Various measures of node importance were computed to identify central proteins within the network, including degree (b), betweenness centrality (c), eigenvector centrality (d), PageRank (e), closeness centrality (f), clustering coefficient (g), and degree centrality (h). The bar represents the average value of each feature and error bars represent the standard deviation value of each feature. The *P*-value of the one-way ANOVA test comparing the target group with the previous group (e.g. the *P*-value is 0.172 compared degrees of proteins with prediction probabilities from 0.5 to 0.6 with proteins with probabilities from 0.6 to 0.7) was shown on/below the bar.

We construct the RNA granule proteome PPI community (*n* = 6,600, Fig. 4a) from the STRING database (36) and employ seven metrics: degree, betweenness centrality (37), eigenvector centrality (38), PageRank (39), closeness centrality (40), clustering coefficient, and degree centrality, to discern key nodes contributing significantly to the community (Fig. 4b–h). This demonstrates a conspicuous increasing trend in all seven community importance metrics, ranging from predicted low- to high-probability (i.e. prediction probability from 0.5 to 1.0) RNA granule proteins. It indicates that RNA granule cores contain a dense PPI network (5). Notably, most of the central RNA granule proteins are newly predicted by our models, with only 33% (207 in 626) of the proteins involved in the training set, as listed in Table S2. The highly evaluated and popular SG proteins in heterogeneous experimental SG proteomes also perform high centrality with dense interactions in our RNA granule community. For example, the YBX1 (41) and UPF1 (42) present a central role in the RNA granule PPI network with respect to their 0.99 and 0.97 percentile ranks of their PageRank values, listed in Supplementary material. It indicates the centrality of these proteins beyond over 97% of proteins in the RNA granule PPI community.

#### Central RNA granule proteins are prominent to RNA granule functions and formations

To further assess the generalization of biomolecular and community grammars in the RNA granule community, we collect the protein components of typical RNA granules with non-RNA granules and compare the components of diverse typical RNA granules (i.e. PB, SG, ribonucleoprotein granule, Cajal body, P granule, chromatoid body, nuclear body, and Midbody) and typical non-RNA granules (lipid droplets, PML bodies, and postsynaptic densities) in Table S3 and Figs. S20 and S21. Generally, classic RNA granules, such as PB, cytoplasmic SG, and Cajal body, tend to contain more both high propensity (e.g. probabilities over 0.7) and high centrality RNA granule proteins in the RNA granule PPI community in Fig. S20. Specifically, the popular and central RC3H1, RC3H2, VCP, and G3BP1 across diverse RNA granules (Fig. S20) play pivotal roles in the fundamental functionality of RNA granules (e.g. mRNA translation, mRNA stability (43), and stress responses (43)). The results highlight the significance of PPIs on the functionality of key RNA granule proteins. Additionally, highly popular RNA granule proteins tend to show high prediction biomolecular propensities and high centrality in the RNA granule community among highly heterogeneous SG proteomes (Figs. S21 and S22). Despite this heterogeneity, consistent with the results in Fig. S20, crucial and well-studied RNA granule proteins, including G3BP1, RC3H1, and RC3H2, emerge among all the four SG proteomes with high predicted biomolecular propensities and high centrality in the community. The result suggests high biomolecular propensities and central roles of RNA granule proteins in the RNA granule PPI community may contribute to the stability of RNA granule formation and functionality against the component heterogeneity without the help of membranes.

#### Central proteins contribute to the main characteristics

To explore the relationship between the main characteristics of RNA granules and the centrality of their protein components in the RNA granule PPI network, we conduct a Gene Ontology (GO) enrichment analysis on identified RNA granule proteins with different centrality, as presented in Fig. S23. Proteins with higher centrality (e.g. higher percentile ranks) show a more significant correlation with fundamental RNA granule processes, such as RNA binding and mRNA binding. The analysis reveals a clear association between the centrality of identified RNA granule proteins and their critical biological roles in RNA granule formation and functionality. Consequently, the clear tendency suggests that central components, exhibiting high prediction propensity and dense PPIs, may contribute to the main characteristics of the formation and functionality of RNA granules, while off-center proteins, with low prediction propensity and limited PPIs, likely contribute specificity in the formation and functionality of RNA granules by interacting with other molecules and responding to environmental cues, as shown in Fig. S24.

### Dense PPI clusters perform key functional subunits of diverse RNA granules

#### Dense PPI clusters extracted

**Fig. 5.**
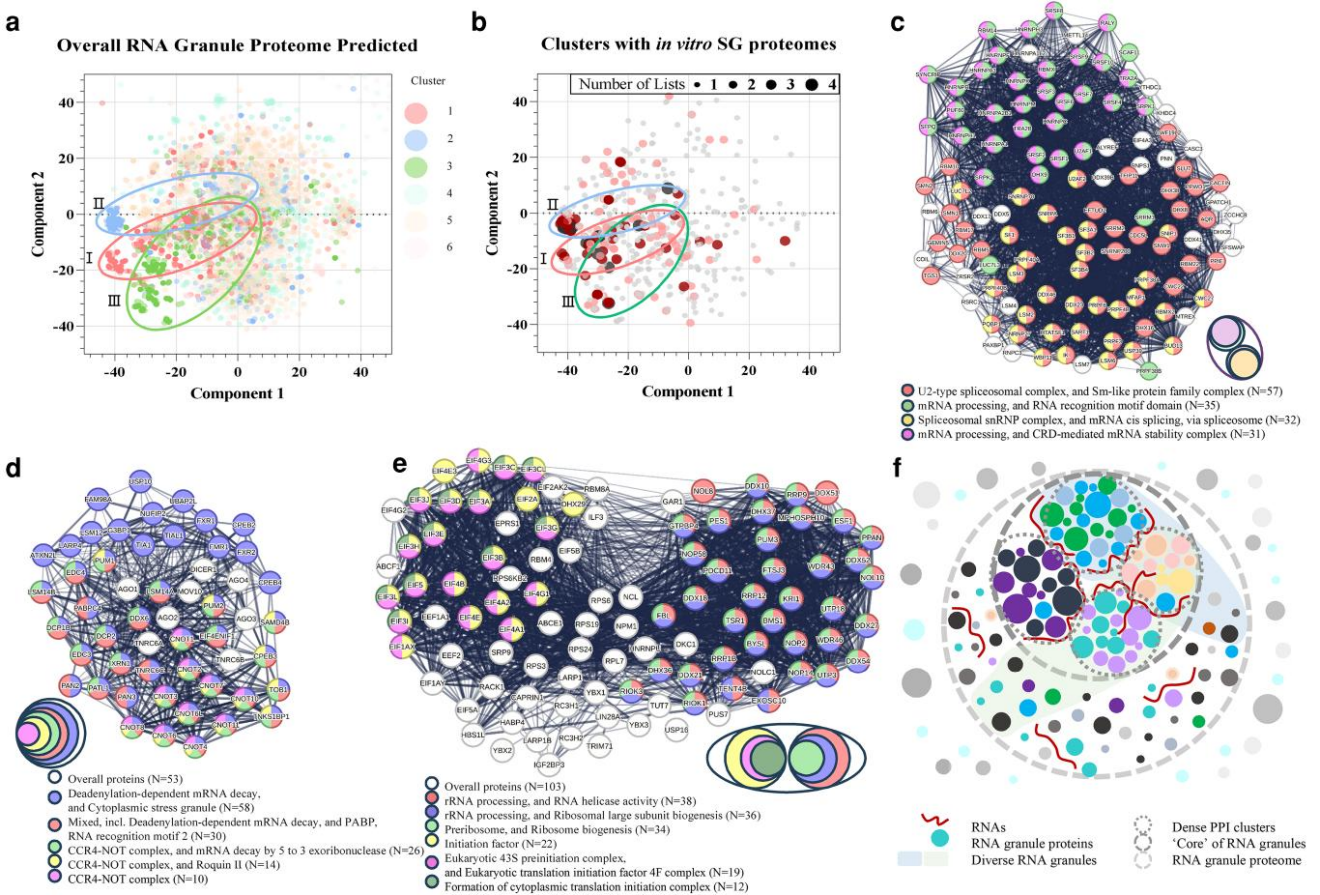
The functional dense PPI clusters in RNA granules. a) We visualized the extracted clusters in the identified RNA granule proteome PPI network (*n* = 6,600). We applied the *t*-distributed Stochastic Neighbor Embedding (*t*-SNE) (44) method to visualize the high-dimensional network in a 2D map, assigning each protein a 2D location (component 1, component 2). b) We visualized the common RNA granule proteins shared in different collected in vitro SG proteomes using the *t*-SNE method (Table S5). The circle I: cluster 1. The circle II: cluster 2. The circle III: cluster 3. c–e) We focused on high-confidence extracted clusters (i.e. clusters 1, 2, and 3) with high-confidence prediction RNA granule proteins (with prediction probabilities over 0.7). We evaluated the potential biological implications of each extracted cluster (i.e. mRNA splicing for cluster 1 in c: *n* = 118, mRNA decay for cluster 2 in d: *n* = 53, rRNA processing and translation for cluster 3 in e: *n* = 103), by exhibiting the related local network clusters significantly (*P* < 0.05) enriched, respectively. We applied the STRING platform to achieve the enrichment analysis. The full lists of proteins in the three extracted clusters are listed in Supplementary material. f) The hypothesis about RNA granule formation and functionality via functional PPI subunits. Our analysis reveals that dense PPI clusters form relatively stable “cores” within RNA granules and contribute to the stability of formation and functions of dynamic RNA granules.

We visualize the complex RNA granule proteome PPI communities in 2D maps, as depicted in Figs. 5a, S25, and S26. There are dense PPI subcommunities involving RNA granule proteins with high prediction probabilities (over 0.7) within the whole cell, cytosol, and nucleus RNA granule proteome PPI communities. To further extract the dense subcommunities in the overall RNA granule proteome PPI network, the analysis applies the Louvain algorithm (45) to detect the dense subcommunity structures in the network. As shown in Figs. 5a and S27–S30, Table S4, and listed in Supplementary material, we focus on high-confidence RNA granules (with prediction probabilities over 0.7) cluster 1 (*n* = 331), cluster 2 (*n* = 193), and cluster 3 (*n* = 239), respectively. These high-confidence RNA granule clusters show high prediction probabilities (average prediction probabilities of 0.83, 0.84, and 0.81) and high community centrality (average percentile ranks of PageRank of 0.72, 0.70, and 0.75) capturing the main biological implications of each cluster (Fig. S30 versus Fig. S28). In addition, the three key clusters exhibit essential biological implications related to RNA granule functionality, such as mRNA splicing (cluster 1), translation and mRNA decay (cluster 2), and rRNA processing with translation (cluster 3) according to the GO enrichment analysis (Figs. S28 and S30). In addition, we analyze the distribution of typical RNA and non-RNA granule components in dense clusters (Figs. S31 and S32). We find that typical RNA granules predominantly contain these extracted dense clusters, while typical non-RNA granules show distinct protein localization patterns. Visualizing the protein distribution of the heterogeneous experimental SG proteomes in Figs. 5b and S33 and Table S5 reveals a notable distribution of popular SG components from highly diverse experimental proteomes within the three selected central clusters. For instance, the most popular 14 proteins shared among all four SG proteome lists are contained in our three selected clusters. These findings strongly indicate that the extracted dense clusters may contribute to the main characteristics of membraneless RNA granules across heterogeneous stress and cellular contexts.

#### Extracted clusters contribute to the main functions of RNA granules

Exploring the enrichment analysis in the GO category in Figs. 5c, S28, S30, and S34, cluster 1 displays a significant association with biological implications closely related to mRNA splicing, with components of diverse RNA granules (Fig. S35). Meanwhile, cluster 2 and cluster 3 tend to serve as hot regions for mRNA decay, translation, and rRNA processing, shown in Figs. 5d and e and S36–S39, respectively. These findings align with the key functions of RNA granules in RNA metabolism (14), including mRNA splicing, mRNA decay, translation, and rRNA processing. Meanwhile, proteins in the three clusters are enriched as components of diverse RNA granules, encompassing PBs, cytoplasmic SGs, and even Cajal body (in cluster 1), shown in Figs. S35, S37, and S39, respectively. This observation further suggests that these central clusters could play a substantial role in the formation and functionality of diverse RNA granules. Figure 5c–e reveals that many RNA granule proteins in the extracted clusters exhibit their biological roles via interaction with essential PPI complexes, such as U2-type spliceosomal complex for mRNA splicing in cluster 1, CCR4–NOT complex for mRNA decay, and translation in cluster 2, and the cytoplasmic translation initiation complex for translation in cluster 3, respectively. In addition, there are many popular functional protein families housed in the three clusters pivotal for RNA granule formation and functions. For example, HNRNP family (e.g. HNRNPA3, HNRNPF, and HNRNPK) in cluster 1 (Fig. 5c), DEAD box protein family (e.g. DDX20, DDX17, and DDX23) in cluster 1 (Fig. 5c), and the protein synthesis initiation factors (e.g. EIF5, EIF3E, and EIF4B) in cluster 3 (Fig. 5e). These critical local network clusters underscore the significance of dense PPI clusters in the posttranscriptional regulation of gene expression (e.g. mRNA decay) in RNA granules. We propose a scientific hypothesis (Fig. 5f) suggesting that dense PPI networks form functional subunits within the stable cores of RNA granules, contributing to their overall dynamic yet stable nature.

## Discussion

This study utilizes machine learning to robustly identify RNA granule proteomes, offering more specific and reliable predictions compared with previous classical LLPS models (8, 9, 24–29). While these models have laid a foundation, they often suffer from limited scope and generalization issues across diverse RNA granule types. Our approach addresses these limitations by integrating a broader range of features, leading to a model that not only predicts RNA granule-associated proteins with higher accuracy but also offers insights into their overarching characteristics.

One of the key strengths of our model is its ability to capture the central characteristics of RNA granules despite their inherent heterogeneity under diverse in vitro conditions. Specifically, the model's predictions exhibit substantial enrichment in biological functions that are well-aligned with the known roles of RNA granules, such as RNA transport, spliceosome activity, and the presence of pivotal protein domains. This enrichment is not merely a repetition of existing knowledge but extends our understanding by quantitatively confirming the robustness of these functions across a more comprehensive proteome dataset. However, this consistency with high-confidence protein candidates, while encouraging, requires critical experimental evaluation.

The discovery of PPI community grammars within the RNA granule proteome is a notable advancement. By focusing on proteins with higher predicted propensities for inclusion in RNA granules, we identify those that occupy central roles within the PPI network. This finding underscores the critical role of PPIs in maintaining RNA granule stability and function. Our identification of three key PPI clusters, characterized by dense interactions and high prediction propensities, suggests the presence of shared functional subunits across different RNA granules. These clusters, which are conserved across diverse granules and stress conditions, likely represent stable “cores” that contribute to the dynamic yet stable nature of RNA granules. Notably, this study does not adequately address the impact of posttranslational modifications (PTMs) on RBPs. These modifications are known to significantly alter the functional properties of individual RBPs and likely play a critical role in shaping the protein interactions within RNA granules.

## Conclusion

In summary, our study applies robust machine learning models to identify RNA granule proteomes from the human proteome and provides new insights into the fundamental role of PPIs in RNA granule biology, proposing that dense PPI clusters may serve as integral functional subunits within these structures.

## Supplementary Material

pgaf093_Supplementary_Data

## Data Availability

Raw data, analysis codes, and step-by-step instructions are accessible via the GitHub repository: (https://github.com/BanZhan/RNAgranuleModel).
