# Supplementary material for: Unraveling biomolecular and community grammars of RNA granules via machine learning
Source: PNAS Nexus. 2025 Mar 19;4(4):pgaf093. doi: 10.1093/pnasnexus/pgaf093 (PMC11952899; doi:10.1093/pnasnexus/pgaf093)
Supplement: pgaf093_Supplementary_Data [file pgaf093_supplementary_data.zip › PNASNEXUS-PNASNEXUS-2024-01330-TRR-s02.docx]

## Unravelling biomolecular and community grammars of RNA granules via machine learning

Zhan Ban, Yuchen Lin, Yan Yan*, Kenneth A. Dawson*

Centre for BioNano Interactions, School of Chemistry, University College Dublin, Dublin 4, Ireland; School of Biomolecular and Biomedical Science, UCD Conway Institute of Biomolecular and Biomedical Research, University College Dublin, Dublin 4, Ireland

*Corresponding authors: yan.yan@cbni.ucd.ie; kenneth.a.dawson@cbni.ucd.ie

**Supplementary Methods**

**Construction of the RNA Granule Dataset**

As machine learning models are data-driven, the reliability and performance of the models largely depend on the dataset used. To construct our dataset, we retrieved samples of RNA granule proteins, including stress granule (SG) proteins, P-body (PB) proteins, and PB or SG (PBSG) proteins, from the RNAgranuleDB version 2.0 (http://rnagranuledb.lunenfeld.ca/, curation: November 2021 for SGs and February 2019 for PBs) as of February 2023 (reference 4 in the main manuscript). Specifically, we focused on human proteins from the database, collecting all tiers (tier 1 to 3, or 4) for our SG, PB, or PBSG datasets, respectively. In addition, we collected all reviewed (Swiss-Prot) human proteins (20423 proteins) from the UniProt (https://www.uniprot.org/, Version: UniProtKB 2023_01) as of March 2023^1^. We filtered out proteins with sequence lengths shorter than 3 residues or truncated the TTN protein to 32756 residues. Overall, we collected 20422 proteins from the human proteome.

**Negative Data Construction**

Studies typically report positive results, resulting in a shortage of negative samples in the RNA granule database and an imbalance between positive and negative samples for machine learning models. In this study, we considered all human proteins in the RNA granule database (RNAgranuleDB) as positive candidates for our models. We employed a random sampling strategy to construct negative samples with the same number of positive samples from the remaining human proteome (excluding all RNA granule protein candidates in the RNAgranuleDB).

Based on previous liquid-liquid phase separation (LLPS) models, the protein sequences in the publicly available protein data bank (PDB) dataset^2^ have a low likelihood of undergoing phase separation and they are commonly used as negative LLPS protein candidates for training relative models (reference 9 in the main manuscript). Therefore, the analysis also applied the PDB dataset from the work (reference 9 in the main manuscript) as negative samples to evaluate the performance of our RNA granule models.

**Physicochemical Features and Amino Acid (aa) Content of Sequences**

Inspired by the classic LLPS model (reference 8 in the main manuscript), we estimated important physicochemical properties and aa content of whole protein sequences and low complexity regions (LCRs) which have been shown to drive LLPS. These physicochemical features included length, molecular weight (mw, Da), isoelectric point (IEP), hydrophobicity (or hydrophilicity), aromaticity, Shannon entropy, cation fraction, protein secondary structures and low complexity. We applied the *ProteinAnalysis* module of *Biopython* package^3^ (Version: 1.80) in Python (Version: 3.8.8) to estimate the mw, IEP, gravy and aromaticity (*i.e.*, aromatic fraction) of protein sequences. The mw values of all residues were summed up to estimate protein mw of each protein sequence. The IEP was calculated following the methods of Bjellqvist et al.^4,5^. The gravy values were calculated to evaluate the hydrophobicity or hydrophilicity of each sequence using the Kyte and Doolittle hydropathy scale^6^, which summed the individual gravy value of each residue in the sequence. In addition, we also estimated hydrophobicity or hydrophilicity by calculating total number or fractions of residues with gravy values lower than -1.5, -2 or -2.5 in each protein sequence, respectively. The aromatic fraction was calculated by determining the relative frequency of phenylalanine, tryptophan, and tyrosine in the whole sequence. The Shannon entropy of individual sequences was estimated using the formula (reference 9 in the main manuscript):

$$H(X)=-\sum_{i=1}^{N=20} P_{i}\log_{2} P_{i} (1)$$

Where the variable *P* represents the frequency of the 20 naturally occurring residues in the given protein sequence. The cation fraction was estimated by calculating the relative frequency of lysine, arginine, and histidine in each protein sequence. As shown in the classic LLPS model, PSAP (reference 8 in the main manuscript), the fractions of protein secondary structures (*i.e.*, alpha-helix, beta-turn, beta-sheet) were estimated as the summed fractions of valine, isoleucine, tyrosine, phenylalanine, tryptophan and leucine (for alpha-helix); asparagine, proline, glycine and serine (for beta-turn) and glutamate, methionine, alanine and leucine (for beta-sheet), respectively.

We defined and estimated the low complexity score by the number of unique residues in each sliding window (20 aas) as the PSAP (reference 8 in the main manuscript) model. The total low complexity scores or fractions were calculated as the number or fraction of aa within sliding windows (20 aas) with low complexity score ≤ 7 in each whole protein sequence, respectively. We estimated the low complexity score of each aa by counting occurrence of the target aa in identified low complexity positions by sliding windows. The low complexity fraction of each aa was estimated by the ratio of the low complexity score of the target aa to total number of collected low complexity windows. The lowest complexity score was estimated by the lowest complexity score of all sliding windows of each whole protein sequence.

1. **mer Features for the Sequences**

We extracted all available 2-mers and 3-mers from the human proteome and determined their fractions as the ratios of occurrence to the sequence length of each protein. To build robust binary RNA granule protein classification models, it is necessary to select protein sequence features that can distinguish RNA granule protein candidates from negative protein candidates. To select important k-mers from human proteome, we counted the total occurrence of each k-mers in overall collected human RNA granule protein candidates from the RNA granule database and estimated the difference between k-mer fractions of collected RNA granule proteins with the k-mer fractions of negative protein candidates randomly selected from human proteome, with one-way ANOVA test using *Stats* module in *Scipy* package (Version 1.9.3) of Python (Version: 3.8.8). Then, we selected top 50 2-mers and 50 3-mers with significantly different occurrences (*p*<0.001) among overall collected human RNA granule proteins from the RNAgranuleDB.

Overall, there are 179 features for each protein sequence involved in our model, including 60 features for fractions in the whole sequence, scores, and fractions in LCRs of 20 common residues, 3 features for the low complexity fraction, the low complexity score, and the lowest complexity score in the overall protein sequence, 16 features related to physicochemical properties, and 50 selected 2-mers and 50 selected 3-mers.

**Building of the Machine Learning Models**

In this study, all RNA granule protein models were constructed using the *scikit-learn* package^7^ (Version 1.3.0) of Python. To achieve high accuracy and robustness, we employed the powerful ensemble machine learning algorithm, random forest (reference 20 in the main manuscript).

The random forest algorithm is a meta estimator that employs a collection of decision tree models on different subsamples of the dataset. By utilizing averaging techniques, a random forest can enhance predictive accuracy and avoid overfitting effectively (reference 20 in the main manuscript). We set the number of estimators to 2000, the class weight to ‘*balanced*’ to employ class frequencies of the input data to automatically adjust weights and applied the ‘entropy’ criterion to evaluate the effectiveness of a split in a random forest model, inspired by the classic LLPS model (reference 8 in the main manuscript).

In our analysis, the final SG, PBSG, and PB models (binary classification) were constructed using tier 1 proteins of SG (280 tier 1 SG proteins + 280 negative proteins, *N* = 560), PBSG (473 tier 1 PBSG proteins + 473 negative proteins, *N* = 946), or a combination of tier 1 and tier 2 proteins of PB (198 tier 1 & 2 PB proteins + 198 negative proteins, *N* = 396), respectively.

**Evaluation of the Machine Learning Models**

To prevent overfitting of the models, we randomly divided the sampled dataset into ten folds to apply ten-fold cross-validation to evaluate model’s performance. Therefore, the training and validation sets for each final model comprised 80% and 20% of the overall datasets for SG (*N*=560), PBSG (*N*=946), and PB (*N*=396), respectively. Additionally, we used the unlikely-LLPS PDB dataset as a negative dataset (*i.e.,* the testing set, *N*=1427) to evaluate the performance of the trained models. We also employed random seeds to ensure the reproducibility of our models, as demonstrated in the codes.

To evaluate the models’ predictive capability, we applied six important metrics in the analysis: accuracy, precision, recall, F1 value, the area under the receiver operating characteristic (ROC) curve (AUC) and the area under precision-Recall curve (PR AUC). The metrics were defined as below:

$Accuracy = \frac{TP + TN}{TP + TN + FP + FN}$(2)

$Precision = \frac{TP}{TP + FP}$(3)

$Recall = \frac{TP}{TP + FN}$(4)

$F1 = \frac{Precision\times Recall\times2}{Precision+Recall}$(5)

Where *TP* represents true positives, *TN* stands for true negatives, *FP* for false positives and *FN* for false negatives. Additionally, we utilized AUC values to assess the predictive performance of our RNA granule classification models. To determine the predictive performance at different probability thresholds, we constructed ROC curves. By varying thresholds, we calculated the AUC values, which quantify the model's performance on a scale from 0 to 1. An AUC value of 1 represents a perfect prediction, while an AUC value close to 0.5 typically indicates a random guess. The PR AUC metric assesses the balance between precision and recall at varying decision thresholds. A higher PR AUC value signifies that the model is performing well in terms of achieving high precision while maintaining an acceptable level of recall. A PR AUC value of 1 represents a perfect prediction. The final RNA granule classification model applies the average prediction probability of RNA granule protein scores predicted by ten trained models in the ten-fold cross evaluation. All metrics were calculated using the *scikit-learn* package (Version: 1.3.0) in Python (Version: 3.8.8).

**Sensitivity Analysis of Model Performance**

The performance of individual protein models was influenced by various hyperparameters, including the number of protein features (*i.e.*, physicochemical features and k-mer features), the complexity of machine learning algorithms (*i.e.,* the number of estimators), the number of sampled negative samples from the human proteome, and different powerful machine learning models (*i.e.,* random forest, AdaBoost, gradient boosting, k-nearest neighbors algorithm, and multilayer perceptron). To evaluate the sensitivity of model performance to the hyperparameters, we estimated models’ performance constructed using different protein features (*i.e.,* basic physicochemical features and aa contents, as used in the classic LLPS model (reference 8 in the main manuscript), with 20 k-mers, 100 k-mers, 200 k-mers and all significant (*p* < 0.001) 837 k-mers), different numbers of estimators in random forest algorithm (*i.e.,* 1500, 2000 and 2500) and different ratios of sampled negative samples from the human proteome to positive samples (*i.e.,* 0.5, 1 and 1.5), respectively. We constructed the machine learning classifiers with default settings (‘*estimators’* = 2000 for random forest models, AdaBoost and gradient boosting models, ‘*n_neighbors’* = 5 for k-nearest neighbors models, and *‘max_iter’* = 200 for the multilayer perceptron models) in the *scikit-learn* package (Version: 1.3.0).

**Feature Importance Evaluation**

To evaluate the contribution of each feature in trained machine learning models, we estimated the Gini feature importance scores for each feature from ten trained random forest models in the ten-fold cross validation by using the ‘*feature_importances_*’ function from the *ensemble* module in the *scikit-learn* package (Version: 1.3.0).

In the training process of a random forest classification model, each node within the binary trees seeks the optimal split using Gini impurity, which is a computationally efficient approximation of entropy (reference 20 in the main manuscript). This impurity measures how well a potential split separates the samples of the two classes at a particular node (reference 20 in the main manuscript). The Gini feature importance is essentially a by-product of this process, visualizing the outcome of the implicit feature selection performed by the random forest classification model and the significance of different features in the model^8^.

**Partial Dependence Evaluation**

To visualize the relationship between key protein features and the prediction propensity of an RNA granule protein candidate in our models, we applied the partial dependence method^9^ to estimate the average prediction propensity with actual feature values (*i.e.,* 5, 15, 25, 35, 45, 55, 65, 75, 85 and 95 percentiles) of each selected key feature in the whole human proteome. The method shows how the selected feature affects the average prediction propensity at the proteome level. The partial dependence method assumes the estimated key feature is independent and uncorrelated.

**Key Physicochemical Property Selection**

The distance correlation, Pearson correlation, and feature importance (as described in Feature Importance Evaluation) were applied to evaluate the strength and direction of the relationship, as well as the dependence between each physicochemical feature (*i.e.*, gravy, mw, hydrophobicity or hydrophobicity or hydrophilicity values (HPI) < -1.5, HPI < -2.0, HPI < -2.5, HPI < -1.5 fraction, HPI < -2.0 fraction, HPI < -2.5 fraction, alpha helix, beta turn, beta sheet, length, aromaticity, cation fraction, LCR fraction, IEP and entropy) and the target prediction probability of the selected PBSG model.

Distance correlation can detect both linear and nonlinear dependencies, and it is zero if and only if the variables are independent^10^. Pearson correlation^11^, on the other hand, measures the linear relationship between two variables, varying from -1 to 1. The distance correlation value was estimated using the python package *dcor* (Version 0.6), and the Pearson correlation was estimated using the *Stats* module of the python package *SciPy* (Version 1.9.3).

**Sliding Window Analysis**

We applied the sliding window method to evaluate our RNA granule model performance on key domains and intrinsically disordered regions (IDRs), which determine phase separation capability and RNA binding propensity, respectively. Three important biomarkers were selected for the analysis, including hnRNPA2B1, DDX6 and DCP2. We set the sliding window to 30 residues and calculated the average prediction probability as RNA granule proteins within each sliding window by the ten-fold cross-evaluated selected PBSG models. Then, we calculated and displayed the average values of the top two features (cysteine fraction and gravy) distributed across the highly evaluated RNA granule biomarkers (*i.e.,* hnRNPA2B1, DDX6 and DCP2) within each 30-residue sliding window.

**Model Performance on Published SG Proteomes**

To evaluate the performance of our selected RNA granule models (for SG, PB and PBSG models), we collected published proteins of SGs from papers (*N =* 253 in list 1 (reference 33 in the main manuscript), *N =* 221 in list 2 (reference 5 in the main manuscript), *N =* 486 in list 3 (reference 12 in the main manuscript), and *N =* 172 in list 4 (reference 34 in the main manuscript)). The total number of collected published unique SG proteins is 811. We applied our RNA granule models (*i.e.,* the selected PBSG model) to classify the collected proteins (proteins excluded from the model training set or overall proteins).

**Comparison with Classic LLPS Models**

To evaluate the reliability of our identified RNA granule proteome, we applied classic and widely-used LLPS models to predict the LLPS propensities of the human proteome. These models include the PSAP model (reference 8 in the main manuscript), the DeePhase model (reference 9 in the main manuscript), and the PScore model (reference 24 in the main manuscript). To assess the relative propensities of each protein within all predicted LLPS proteomes, we calculated the average percentile rank of the predicted scores for each protein in the three LLPS models. Additionally, we calculated the percentile rank of the predicted RNA granule propensity of each protein in the human proteome using our RNA granule model. To visualize the relationship between RNA granule prediction propensities from our RNA granule model and LLPS propensities of the human proteome, we classified the human proteome into six groups based on predicted RNA granule probabilities (*i.e.,* 0-0.5, 0.5-0.6, 0.6-0.7, 0.7-0.8, 0.8-0.9 and 0.9-1.0) according to the selected PBSG model. Then, we visualized the distribution of LLPS propensities predicted by different LLPS models in each group of RNA granule proteins with varying RNA granule probabilities.

Because the PScore model only predicts the probability for proteins with at least 140 residues, we calculated the average percentile rank value of predicted LLPS scores for each protein in the human proteome using the three LLPS models. Then, we selected the top 6694 (the same number as the predicted RNA granule proteome with RNA granule probabilities greater than 0.5) or 2225 (the same number as the predicted RNA granule proteome with RNA granule probabilities greater than 0.7) LLPS-prone proteins from the human proteome (*N* = 20422), based on the average percentile ranks of predicted LLPS scores for each protein. Then, we performed enrichment analysis of the selected LLPS-prone proteins and compared the functional enrichment results of LLPS-prone proteins with observed RNA granule candidate proteins, and high-confidence or overall identified RNA granule proteins from the RNAgranuleDB.

**Enrichment Analysis in Enrichr**

To assess the biological implications of the predicted RNA granule proteome, we conducted enrichment analyses using the Kyoto Encyclopedia of Genes and Genomes (KEGG) pathway (2021 version), Gene Ontology (GO) (2023 version), and the InterPro^12^ database (2019 version, accessed July 2023). This analysis aimed to identify enriched pathways in the KEGG database and GO biological process (GOBP), GO molecular function (GOMF), GO cellular component (GOCC), and functionally important domains. We utilized the observed high-confidence RNA granule proteins (*i.e.,* tier 1 proteins for SG, tier 1 & 2 proteins for PBs, and tier 1 proteins for combined PBSG), as well as the high-confidence or overall RNA granule proteome (with average probability over 0.7 or over 0.5) predicted by the selected RNA granule models. We compared the shared terms in the top 20 KEGG pathways, GOBPs, GOMFs, GOCCs and domains enriched by observed high-confidence RNA granule proteins (*i.e.,* tier 1 for SG and PBSG, tier 1 and tier 2 for PB), with overall or high-confidence identified RNA granule proteins (*i.e.,* RNA granule proteins with prediction probabilities ≥ 0.5 or ≥ 0.7) and selected LLPS-prone proteins, according to the -lg(*p* value) for each term. The *p* value was calculated using the Fisher exact test, which is a statistical test for proportions assuming a binomial distribution and independence of the probability of any gene belonging to any test. This test was used to assess the significance of observed differences between groups. For the enrichment analysis, we used Enrichr (https://maayanlab.cloud/Enrichr/), a web-based tool accessed in July 2023^13–15^.

**RBP Collection**

The human RBPs (*N* = 6163) were collected from the RBPbase database (https://apps.embl.de/rbpbase/, version 0.2.1 alpha, access date: Oct. 2023). We identified human RBPs from the RBPbase database by combining 35 lists, including Hs_HEK293-RIC, Hs_HuH7-RIC, Hs_HeLa-RIC, Hs_HeLa-RBDmap, Hs_K562-serIC-chr, Hs_K562-serIC, Hs_HeLa-RNPxl, Hs_HEK293-pCLAP, Hs_microRNA-RIC, Hs_JURKAT-2018-RIC, Hs_JURKAT-2018-eRIC, Hs_HeLa-RICK-hc, Hs_CARIC, Hs_Cyto-eRIC, Hs_Cyto-Ars-eRIC, Hs_Nuc-eRIC, Hs_Nuc-Ars-eRIC, Hs_OOPS_HEK293, Hs_OOPS_MCF10A, Hs_OOPS_U2OS, Hs_PTex_0015, Hs_PTex_015, Hs_PTex_15, Hs_R-Deep, Hs_XRNAX-MCF7, Hs_XRNAX-HeLa, Hs_XRNAX-HEK293, Hs_XRNAX-MCF7-ars, Hs_HEK293-cRIC-SINV, Hs_HEK293-cRIC, knownRBPs-2014-Hs, SONAR-Hs, hasRBD-Pfam-Hs, RBPDB-Hs, and humanRBPs-2021. In total, 5976 RBPs were involved in our filtered human proteome.

#### **Protein-protein interaction (PPI) Network Construction of Identified RNA Granule Proteome**

To evaluate the role of identified RNA granule proteins in the protein community, we used the human protein physical links from functional protein association networks in the STRING dataset (<https://stringdb-downloads.org/download/protein.physical.links.full.v12.0.txt.gz>, Version: 12.0, access date: July 2023). The STRING database integrates diverse sources of experimental evidence, co-expression data, and computational predictions to construct a comprehensive network of functional prediction associations (reference 36 in the main manuscript).

The analysis focused on human protein physical links with experimental proofs, including 947158 links and 19126 unique proteins from the STRING dataset. We constructed the overall RNA granule proteome PPI community (*N* = 6600) with the identified RNA granule proteome (with predicted propensities > 0.5 according to our model) and their physical links.

#### **Node Importance in PPI Network of the RNA Granule Proteome**

In this study, we employed graph theory to analyze the identified RNA granule proteome PPI network. We constructed the network and performed node importance analyses using the Python (Version: 3.8.8) package *NetworkX* (Version 3.0)^16^, where nodes in the graph represented proteins, and edges represented interactions between proteins.

To identify key proteins with central roles in the network, we computed various metrics of node importance. The measures of node importance included degree, degree centrality, betweenness centrality, eigenvector centrality, PageRank, closeness centrality, and clustering coefficient. Degree counts the number of interactions of each protein using the ‘*degree*’ module of the *NetworkX* package. Degree centrality quantifies the number of interactions a specific protein has with other proteins using the ‘*degree_centrality’* module of the *NetworkX* package. Betweenness centrality measures the extent to which a protein acts as a “bridge” between different parts of the network using the ‘*betweenness_centrality’* module of the *NetworkX* package^17^. Eigenvector centrality measures the influence of a protein within the network, considering both direct and indirect connections using the ‘*eigenvector_centrality’* module of the *NetworkX* package (reference 38 in the main manuscript). Like eigenvector centrality, PageRank measures the influence of a protein within the network, accounting for both direct and indirect connections using the *‘pagerank’* module of the *NetworkX* package (reference 39 in the main manuscript). Closeness centrality measures how close a protein is to all other proteins in the network using the ‘*closeness_centrality’* module of the *NetworkX* package (reference 40 in the main manuscript). Clustering coefficient measures the extent to which a protein’s interaction partners also interact with each other in the unweighted network using ‘*clustering’* module of *NetworkX* package.

#### **Protein Component Collection of Typical RNA Granules and Non-RNA Granules**

In this analysis, we systematically collected protein components from commonly classified RNA granules, using defined terms and GO annotations for precise categorization. The selected RNA granules include: PB (term: P-body; GO: 0000932), SG (term: cytoplasmic stress granule; GO: 0010494), ribonucleoprotein granule (term: cytoplasmic ribonucleoprotein granule; GO: 0036464), Cajal body (term: Cajal body; GO: 0015030), P granule (term: P granule; GO: 0043186), chromatoid body (term: Chromatoid body; GO: 0033391), nuclear body (term: nuclear body; GO: 0016604), and midbody (term: Midbody; GO: 0030496).

Additionally, we collected protein components from three biomolecular condensates that are not typically classified as RNA granules. These biomolecular condensates include postsynaptic density (term: postsynaptic density; GO: 0014069), lipid droplet (term: lipid droplet; GO: 0005811), and PML body (term: PML body; GO: 0016605).

The protein components were retrieved from the QuickGO database^18^ (https://www.ebi.ac.uk/QuickGO/, version 2023-10-06, access date: Oct. 2023). **Table S3** summarizes the collected components.

#### **PPI Network of RNA Granules in Different Cellular Locations**

Various cellular compartments, including the cytosol and nucleus, serve as sites for the main functions of RNA granules (RNA metabolism and regulation). In the assessment of community grammars (*i.e.,* node importance of each protein) within the RNA granule proteome PPI network across various cellular locations, we compiled human proteins from three crucial cellular compartments: cytosol (term: cytosol; GO: 0005829; *N* = 2652), nucleus (term: nucleus; GO: 0005634; *N* = 2752), and ER (term: endoplasmic reticulum; GO: 0005783; *N* = 325). The comprehensive protein selection was obtained from the QuickGO database^18^ (https://www.ebi.ac.uk/QuickGO/, version 2023-10-06, access date: Oct. 2023).

We then extracted community properties (*i.e.,* PageRank value of each protein and the percentile ranks of these values in the overall RNA granule proteome PPI network) for the collected proteins from the three cellular locations within the entire RNA granule PPI network.

#### **t-distributed Stochastic Neighbor Embedding (t-SNE) Visualization**

To efficiently unravel the intricate structure of the RNA granule proteome PPI network, we employed the t-SNE technique (reference 45 in the main manuscript). The primary objective of applying t-SNE was to visualize the high-dimensional network in a two-dimensional map, assigning each protein a two-dimensional location (component 1, component 2) for intuitive interpretation.

t-SNE is particularly well-suited for revealing complex local structures, such as clustered subcommunities, by representing similarities between data points in the original or embedded high-dimensional space using Gaussian joint probabilities or Student’s t-distributions (reference 45 in the main manuscript). In our analysis, the t-SNE technique was completed, and the resulting two-dimensional locations of each protein within the entire RNA granule proteome PPI network (*N* = 6600), as identified by our RNA granule model, were extracted.

The t-SNE visualization was implemented using the *‘TSNE’* module of the *scikit-learn* package (Version: 1.3.0) in Python (Version: 3.8.8). We configured the ‘*n_components’* as 2, while keeping other parameters as their default values.

#### **Distribution of Published SG Proteomes in the PPI Network**

To assess the performance of our selected RNA granule model, we gathered protein lists for SGs from four published papers (*N =* 253 in the list 1 (reference 33 in the main manuscript), *N =* 221 in the list 2 (reference 5 in the main manuscript), *N =* 486 in the list 3 (reference 12 in the main manuscript), and *N =* 172 in the list 4 (reference 34 in the main manuscript)). The total number of collected published SG proteins is 811. Notably, we classified these proteins using our RNA granule model.

To further evaluate the propensity of each collected SG protein component as an RNA granule protein, we utilized the prediction probability provided by our RNA granule protein model, as shown in **Supplementary Files**. Subsequently, we visualized the locations of the collected published SG proteomes in a two-dimensional map using protein locations on the whole RNA granule proteome PPI network by the t-SNE method.

#### **Extraction of RNA Granule Clusters**

To identify critical functional subcommunities within the RNA granule proteome PPI network, we applied the Louvain community detection algorithm to detect well-connected communities within the overall RNA granule proteome PPI network.

Louvain algorithm is simple and elegant^19^, which simplifies the community detection task of uncovering a network’s community structure via optimizing modularity. There are two steps for the Louvain algorithm. At the outset, the algorithm segregates each node within the RNA granule proteome into its own distinct community. Subsequently, it systematically investigates potential enhancements in modularity by evaluating the benefits of relocating each node to neighboring communities. In the absence of discernible improvements, such as an increase in modularity captured by the quality function, the node retains its original community^20^.

We completed the community detection using the ‘*louvain_communities*’ module of the *NetworkX* package (Version 3.0)^21^ in Python (Version: 3.8.8). We kept all parameters as their default values, and we set the random seed to make sure reproducibility of results.

#### **Functional Enrichment Analysis**

To evaluate the biological implications of extracted RNA granule clusters and assess their reliability, we performed functional enrichment analysis in the Enrichr (https://maayanlab.cloud/Enrichr/), a web-based tool accessed on July 2023^13–15^ or in the STRING dataset (https://version-12-0.string-db.org/, Version: 12.0, access date: July 2023).

In the Enrichr, we conducted the GO (2023 version) enrichment analysis to identify enriched BPs for the extracted clusters. We showed the top 10 significantly enriched BPs by extracted cluster proteins, based on the -lg(*p* values) of each term.

In the STRING, we constructed the PPI network for the extracted clusters. In the STRING network, the edges between two proteins represent physical and functional protein associations and the network lines between two proteins indicate the strength of data support. We applied all available active interaction sources to construct the networks, including text mining, databases, experiments, co-expression, gene fusion, neighborhood and co-occurrence. Meanwhile, we establish a minimum required interaction score threshold of 0.4 (the default value).

The constructed PPI network for overall Cluster 1 (with RNA granule proteins with predicted probabilities ≥ 0.5) comprised 567 nodes, 13854 edges, an average node degree of 48.9, an average local clustering coefficient of 0.495, and a PPI enrichment *p* value < 1.0e-16.

The constructed PPI network for high-confidence Cluster 1- (with RNA granule proteins with predicted probabilities ≥ 0.7) comprised 331 nodes, 7998 edges, an average node degree of 48.3, an average local clustering coefficient of 0.538, and a PPI enrichment *p* value < 1.0e-16.

The constructed PPI network for overall Cluster 2 (with RNA granule proteins with predicted probabilities ≥ 0.5) comprised 376 nodes, 2697 edges, an average node degree of 14.3, an average local clustering coefficient of 0.522, and a PPI enrichment *p* value < 1.0e-16.

The constructed PPI network for high-confidence Cluster 2- (with RNA granule proteins with predicted probabilities ≥ 0.7) comprised 193 nodes, 1565 edges, an average node degree of 16.2, an average local clustering coefficient of 0.528, and a PPI enrichment *p* value < 1.0e-16.

The constructed PPI network for overall Cluster 3 (with RNA granule proteins with predicted probabilities ≥ 0.5) comprised 495 nodes, 15923 edges, an average node degree of 64.3, an average local clustering coefficient of 0.542, and a PPI enrichment *p* value < 1.0e-16.

The constructed PPI network for high-confidence Cluster 3- (with RNA granule proteins with predicted probabilities ≥ 0.7) comprised 239 nodes, 4305 edges, an average node degree of 36, an average local clustering coefficient of 0.562, and a PPI enrichment *p* value < 1.0e-16.

The constructed PPI network for overall Cluster 4 (with RNA granule proteins with predicted probabilities ≥ 0.5) comprised 1174 nodes, 23091 edges, an average node degree of 39.3, an average local clustering coefficient of 0.403, and a PPI enrichment *p* value < 1.0e-16.

The constructed PPI network for high-confidence Cluster 4- (with RNA granule proteins with predicted probabilities ≥ 0.7) comprised 420 nodes, 5382 edges, an average node degree of 25.6, an average local clustering coefficient of 0.425, and a PPI enrichment *p* value < 1.0e-16.

The constructed PPI network for overall Cluster 5 (with RNA granule proteins with predicted probabilities ≥ 0.5) comprised 2862 nodes, which is too large to be analyzed in the STRING website.

The constructed PPI network for high-confidence Cluster 5- (with RNA granule proteins with predicted probabilities ≥ 0.7) comprised 768 nodes, 5918 edges, an average node degree of 15.4, an average local clustering coefficient of 0.389, and a PPI enrichment *p* value < 1.0e-16.

The constructed PPI network for overall Cluster 6 (with RNA granule proteins with predicted probabilities ≥ 0.5) comprised 829 nodes, 7679 edges, an average node degree of 18.5, an average local clustering coefficient of 0.408, and a PPI enrichment *p* value < 1.0e-16.

The constructed PPI network for high-confidence Cluster 6- (with RNA granule proteins with predicted probabilities ≥ 0.7) comprised 192 nodes, 594 edges, an average node degree of 6.19, an average local clustering coefficient of 0.461, and a PPI enrichment *p* value < 1.0e-16.

Subsequently, we conducted additional enrichment analysis on the PPI networks in the STRING database, encompassing GOBP, GOCC and local network cluster (STRING) analyses.

**Statistics Analysis**

All statistical analyses were conducted using Python (version 3.8.6), GraphPad Prism (version 9.0.0) or OriginPro 2023b software (Learning Edition). The Pearson method was used to calculate all correlation coefficients unless specified otherwise, the one-way ANOVA in the *SciPy* package^22^ was applied to calculated *p* value, and all confidence levels of the interval were set to 0.95 unless specified otherwise.

**Supplementary References:**

(1) Apweiler, R.; Bairoch, A.; Wu, C. H.; Barker, W. C.; Boeckmann, B.; Ferro, S.; Gasteiger, E.; Huang, H.; Lopez, R.; Magrane, M. UniProt: The Universal Protein Knowledgebase. *Nucleic Acids Res.* **2004**, *32* (suppl_1), D115–D119.

(2) Berman, H.; Westbrook, J.; Feng, Z.; Gilliland, G.; Bhat, T.; Weissig, H.; Shindyalov, I.; Bourne, P. The Protein Data Bank. *Nucleic Acids Res.* **2000**, *28* (1), 235–242.

(3) Cock, P.; Antao, T.; Chang, J.; Chapman, B.; Cox, C.; Dalke, A.; Friedberg, I.; Hamelryck, T.; Kauff, F.; Wilczynski, B.; de Hoon, M. Biopython: Freely Available Python Tools for Computational Molecular Biology and Bioinformatics. *BIOINFORMATICS* **2009**, *25* (11), 1422–1423.

(4) Bjellqvist, B.; Hughes, G. J.; Pasquali, C.; Paquet, N.; Ravier, F.; Sanchez, J.-C.; Frutiger, S.; Hochstrasser, D. The Focusing Positions of Polypeptides in Immobilized pH Gradients Can Be Predicted from Their Amino Acid Sequences. *Electrophoresis* **1993**, *14* (1), 1023–1031.

(5) Bjellqvist, B.; Basse, B.; Olsen, E.; Celis, J. E. Reference Points for Comparisons of Two‐dimensional Maps of Proteins from Different Human Cell Types Defined in a pH Scale Where Isoelectric Points Correlate with Polypeptide Compositions. *Electrophoresis* **1994**, *15* (1), 529–539.

(6) Kyte, J.; Doolittle, R. F. A Simple Method for Displaying the Hydropathic Character of a Protein. *J. Mol. Biol.* **1982**, *157* (1), 105–132.

(7) Pedregosa, F.; Varoquaux, G.; Gramfort, A.; Michel, V.; Thirion, B.; Grisel, O.; Blondel, M.; Prettenhofer, P.; Weiss, R.; Dubourg, V. Scikit-Learn: Machine Learning in Python. *J. Mach. Learn. Res.* **2011**, *12*, 2825–2830.

(8) Menze, B. H.; Kelm, B. M.; Masuch, R.; Himmelreich, U.; Bachert, P.; Petrich, W.; Hamprecht, F. A. A Comparison of Random Forest and Its Gini Importance with Standard Chemometric Methods for the Feature Selection and Classification of Spectral Data. *BMC Bioinformatics* **2009**, *10* (1), 213.

(9) Molnar, C. *Interpretable Machine Learning*; Lulu. com, 2020.

(10) Székely, G. J.; Rizzo, M. L.; Bakirov, N. K. Measuring and Testing Dependence by Correlation of Distances. **2007**.

(11) Pearson, K. VII. Note on Regression and Inheritance in the Case of Two Parents. *Proc. R. Soc. Lond.* **1895**, *58* (347–352), 240–242.

(12) Mitchell, A. L.; Attwood, T. K.; Babbitt, P. C.; Blum, M.; Bork, P.; Bridge, A.; Brown, S. D.; Chang, H.-Y.; El-Gebali, S.; Fraser, M. I. InterPro in 2019: Improving Coverage, Classification and Access to Protein Sequence Annotations. *Nucleic Acids Res.* **2019**, *47* (D1), D351–D360.

(13) Chen, E. Y.; Tan, C. M.; Kou, Y.; Duan, Q.; Wang, Z.; Meirelles, G. V.; Clark, N. R.; Ma’ayan, A. Enrichr: Interactive and Collaborative HTML5 Gene List Enrichment Analysis Tool. *BMC Bioinformatics* **2013**, *14* (1), 1–14.

(14) Kuleshov, M. V.; Jones, M. R.; Rouillard, A. D.; Fernandez, N. F.; Duan, Q.; Wang, Z.; Koplev, S.; Jenkins, S. L.; Jagodnik, K. M.; Lachmann, A. Enrichr: A Comprehensive Gene Set Enrichment Analysis Web Server 2016 Update. *Nucleic Acids Res.* **2016**, *44* (W1), W90–W97.

(15) Xie, Z.; Bailey, A.; Kuleshov, M. V.; Clarke, D. J.; Evangelista, J. E.; Jenkins, S. L.; Lachmann, A.; Wojciechowicz, M. L.; Kropiwnicki, E.; Jagodnik, K. M. Gene Set Knowledge Discovery with Enrichr. *Curr. Protoc.* **2021**, *1* (3), e90.

(16) Hagberg, A.; Swart, P.; S Chult, D. *Exploring Network Structure, Dynamics, and Function Using NetworkX*; Los Alamos National Lab.(LANL), Los Alamos, NM (United States), 2008.

(17) Brandes, U. A Faster Algorithm for Betweenness Centrality. *J. Math. Sociol.* **2001**, *25* (2), 163–177.

(18) Binns, D.; Dimmer, E.; Huntley, R.; Barrell, D.; O’donovan, C.; Apweiler, R. QuickGO: A Web-Based Tool for Gene Ontology Searching. *Bioinformatics* **2009**, *25* (22), 3045–3046.

(19) Traag, V. A.; Waltman, L.; Van Eck, N. J. From Louvain to Leiden: Guaranteeing Well-Connected Communities. *Sci. Rep.* **2019**, *9* (1), 5233.

(20) Wang, S.; He, X.; Du, J. Scientific Commentaries Are Dealing with Uncertainty and Complexity in Science. *Inf. Process. Manag.* **2024**, *61* (4), 103707.

(21) Hagberg, A.; Swart, P.; S Chult, D. *Exploring Network Structure, Dynamics, and Function Using NetworkX*; Los Alamos National Lab.(LANL), Los Alamos, NM (United States), 2008.

(22) Virtanen, P.; Gommers, R.; Oliphant, T. E.; Haberland, M.; Reddy, T.; Cournapeau, D.; Burovski, E.; Peterson, P.; Weckesser, W.; Bright, J. SciPy 1.0: Fundamental Algorithms for Scientific Computing in Python. *Nat. Methods* **2020**, *17* (3), 261–272.


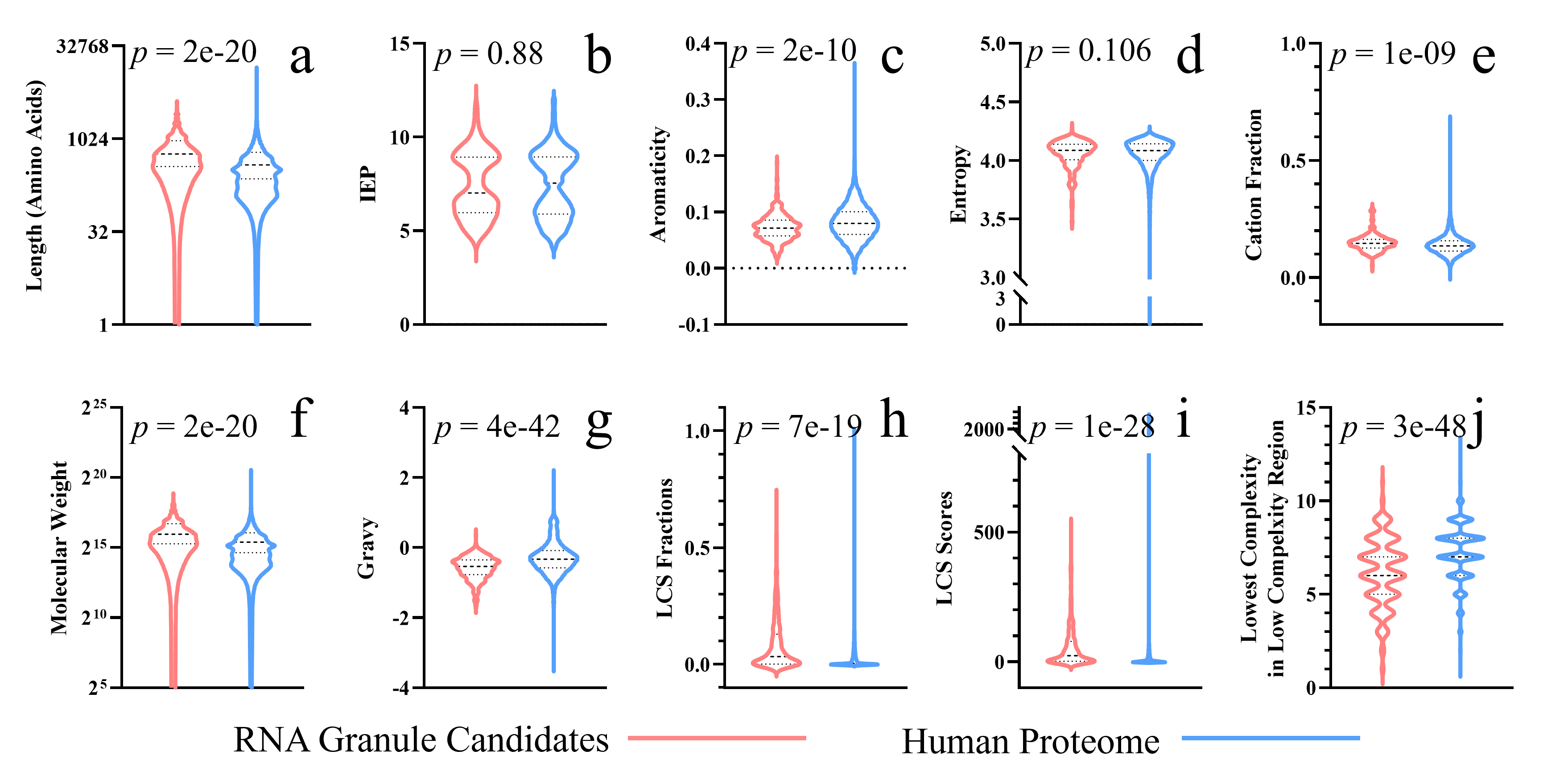
**Fig. S1. Comparison between RNA granule protein candidates and the human proteome on selected physical-chemical properties.** We compared the selected physicochemical properties of observed high-confidence RNA granule protein candidates (*i.e.,* tier 1 P-body or stress granule (PBSG) proteins, *N* = 473) from the RNAgranuleDB with the human proteome (excluding all RNA granule proteins in the RNAgranuleDB, *N* = 15697). We applied the one-way ANOVA to calculate the *p* values.

**Fig. S2. Distinct distribution of k-mer fractions in RNA granule protein candidates (tier 1 proteins) compared to the human proteome and collected RNA binding proteins (RBPs).** We compared the selected aa (*i.e.,* 1-mer) contents (**a&d**), 2-mer contents (**b&e**) and 3-mer contents (**c&f**) of observed RNA granule protein candidates (*N* = 473) with the human proteome (excluding all RNA granule proteins in the RNAgranuleDB, *N* = 15697) or selected RNA binding proteins (RBPs, *N* = 5976), respectively. To assess statistical significance, we applied one-way ANOVA to calculate *p* values comparing each k-mer fraction in tier 1 (T1) RNA granule protein candidates with the corresponding fractions in the human proteome (*N*=15697) or the collected RBPs. The occurrence counts the number of proteins containing the target k-mer among the T1 RNA granule proteins or collected RBPs, respectively. The fold change measures the ratios of the average fraction in the T1 proteins (or the collected RBPs) to the average fraction in the human proteome (or T1 RNA granule proteins) for each k-mer. L: leucine. C: cysteine. D: aspartate. P: proline. G: glycine. H: histidine. RG: arginine-glycine. FL: phenylalanine-leucine. DR: aspartate-arginine. PP: proline-proline. HT: histidine-threonine. EC: glutamate-cysteine. GYG: glycine-tyrosine-glycine.

**Fig. S3. Distinct distribution of k-mer fractions in RNA granule protein candidates (overall tier 1 to tier 4 protein candidates) compared to the collected RBPs.** We compared the selected aa (*i.e.,* 1-mer) contents (**a**), 2-mer contents (**b**) and 3-mer contents (**c**) of overall RNA granule protein candidates (*N* = 4725) and selected RBPs (*N* = 5976). To assess statistical significance, we applied one-way ANOVA to calculate *p* values comparing each k-mer fraction in overall RNA granule protein candidates with the corresponding fractions in collected RBPs. The occurrence counts the number of proteins containing the target k-mer among the collected RBPs (N = 5976). The fold change measures the ratios of the average fraction in collected RBPs to the average fraction in overall RNA granule protein candidates for each k-mer. C: cysteine. H: histidine. D: aspartate. HT: histidine-threonine. AI: alanine-isoleucine. EC: glutamate-cysteine. HTG: histidine-threonine-glycine. KPY: lysine-proline-tyrosine. CGK: cysteine-glycine-lysine. ECG: glutamate-cysteine-glycine.


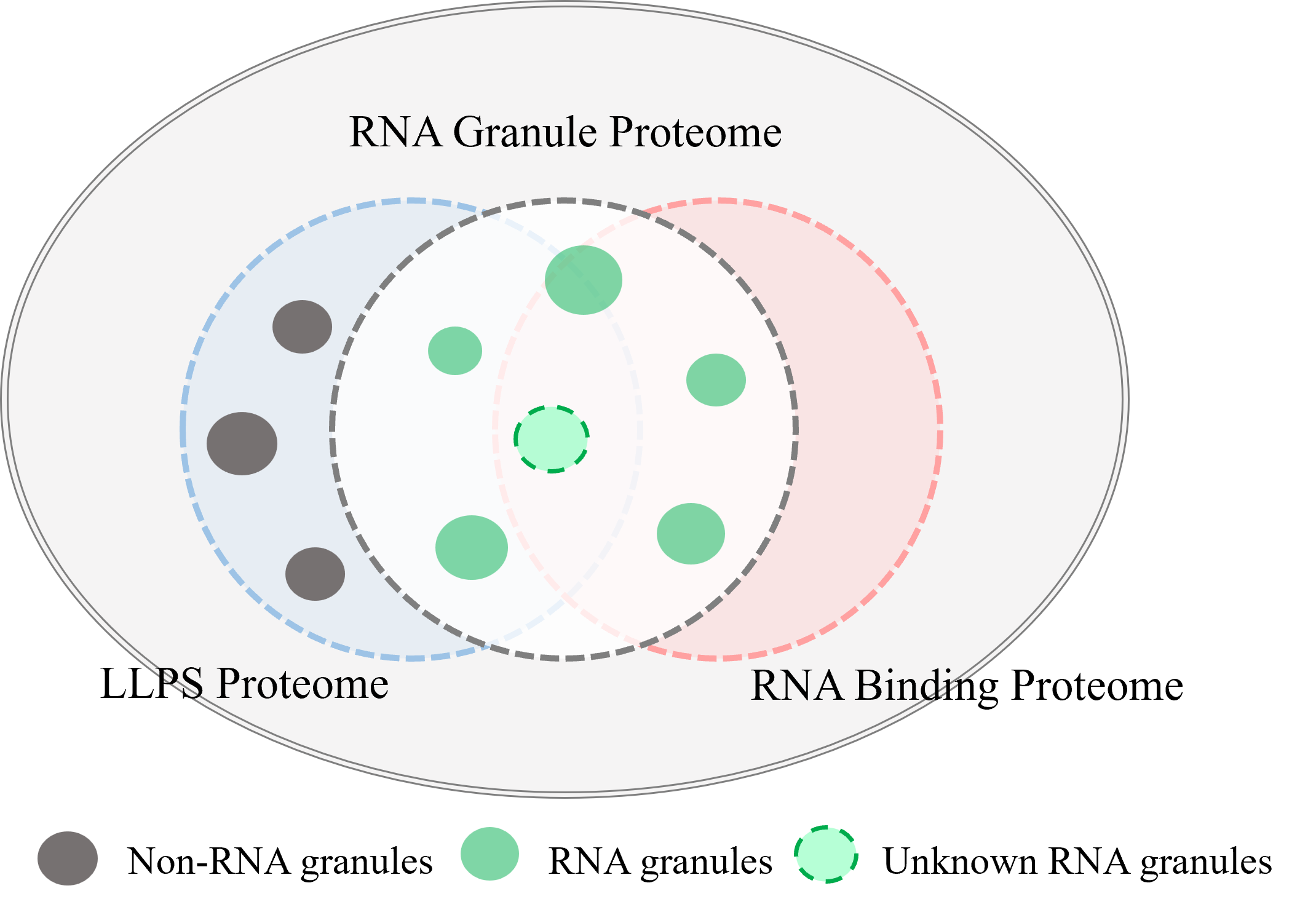
**Fig. S4. The biomolecular mechanisms distinguishing the RNA granule proteome from the liquid-liquid phase separation (LLPS) proteome and the RNA binding proteome.** The RNA granules tend to contain more protein components with larger (*i.e.,* high mws) and hydrophobic residues (*i.e.,* protein gravy values < 0) compared with predicted LLPS proteomes (Fig. 3 in the main manuscript). As shown in Fig. S2, the observed high-confidence RNA granule protein candidates (*i.e.,* the tier 1 RNA granule protein candidates) tend to show distinct biomolecular patterns with the RNA binding proteome.


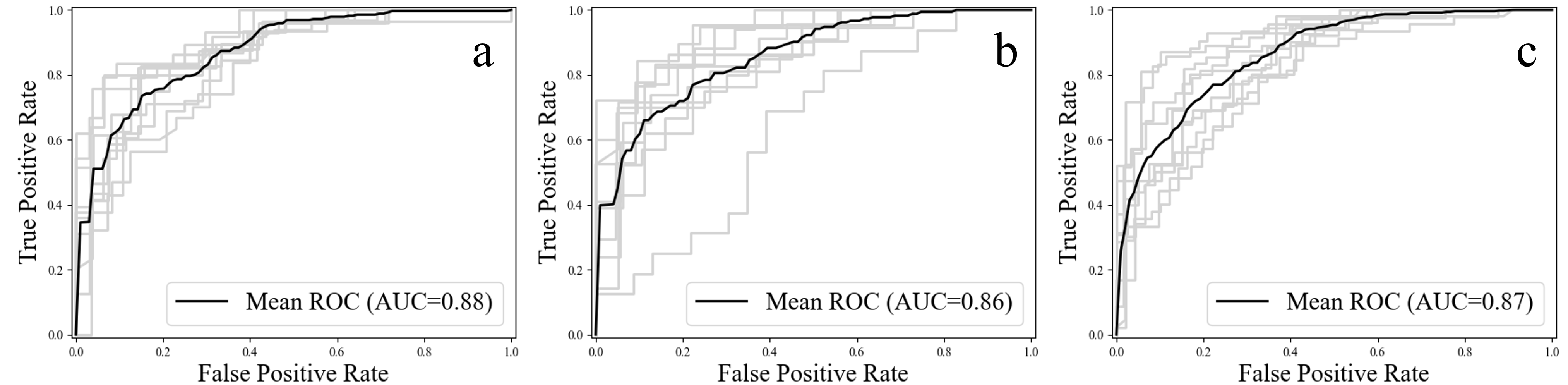
**Fig. S5. Model performance of SG, PB, and PBSG classifiers.** The analysis utilized ten-fold cross-evaluation to estimate the model prediction performance, quantified by the average AUC (represented by the black line) on testing sets for SG (a), PB (b), and PBSG (c) proteins, respectively. The gray lines illustrate the ten-fold evaluation performance of each model on each fold. The true positive rate, also known as sensitivity, is defined as the likelihood of the model correctly predicting a positive protein (*i.e.,* an RNA granule protein) given that the actual protein is indeed positive. The true negative rate, also known as specificity, represents the probability that the model accurately identifies a negative protein (*i.e.,* a non-RNA granule protein) when the actual protein is indeed negative.


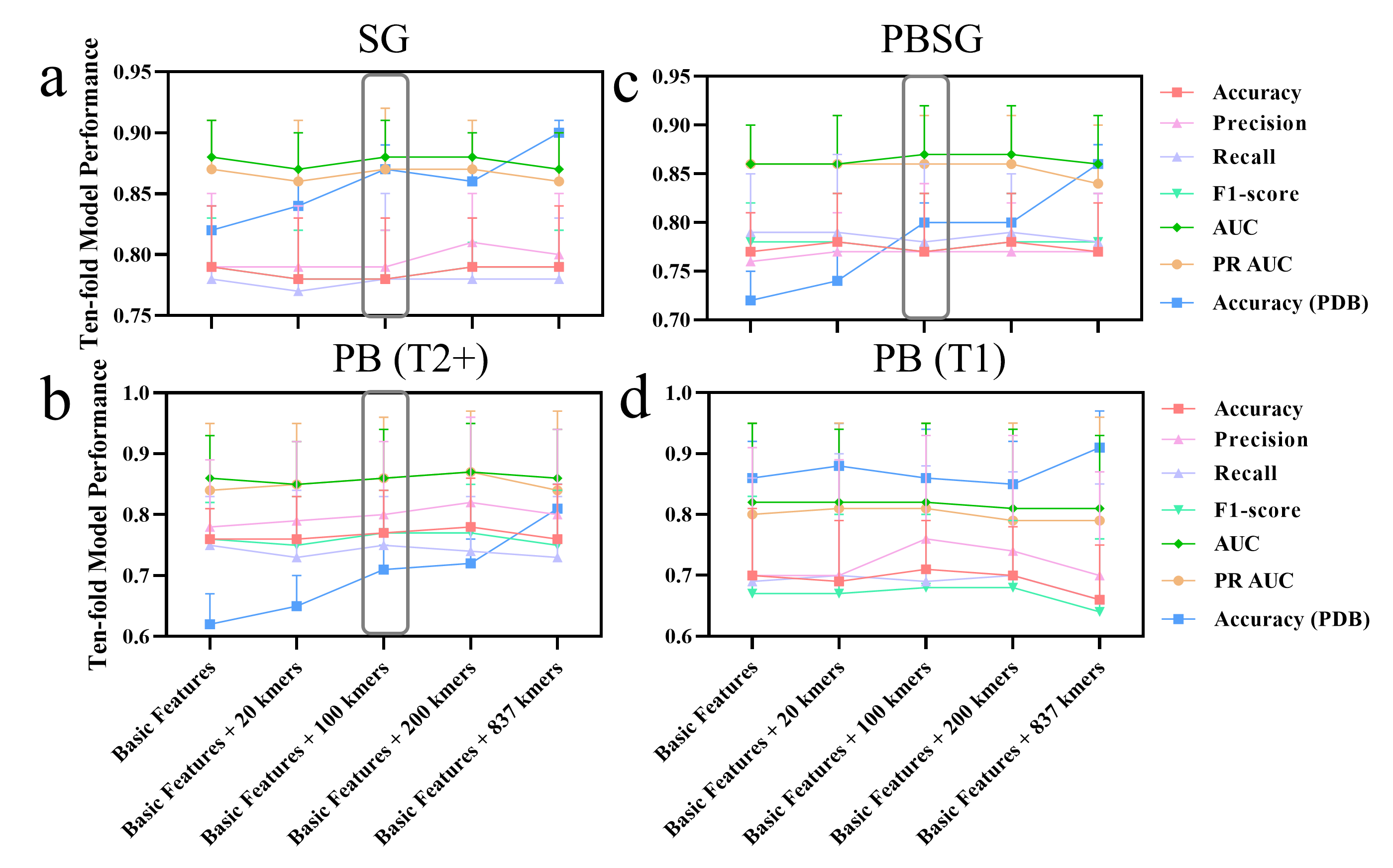


**Fig. S6. Model performance evaluation on various 2-mer and 3-mer contents with observed high-confidence RNA granule protein candidates (tier 1 SG proteins in a, tier 1&2 PB proteins in b, tier 1 PBSG proteins in c, and tier 1 PB proteins in d) from the RNAgranuleDB, employing the ten-fold cross-evaluation method.** Basic features include basic physicochemical properties (*N* = 19) and aa contents (*N* = 40). The k-mers were selected according to their abundance in observed RNA granule proteins, with significant difference (*p* ≤ 0.001) between target k-mer fractions of the selected RNA granule protein candidates and the k-mer fractions of the overall negative candidates in the human proteome. The terms '20,' '100,' '200,' and '837' k-mers refer to 2-mers (*N* = 10) and 3-mers (*N* = 10); 2-mers (*N* = 50) and 3-mers (*N* = 50); 2-mers (*N* = 100) and 3-mers (*N* = 100); and all 2-mers (*N* = 272) and 3-mers (*N* = 565) that show significant differences (*p* ≤ 0.001) compared with overall negative candidates from the human proteome, respectively.


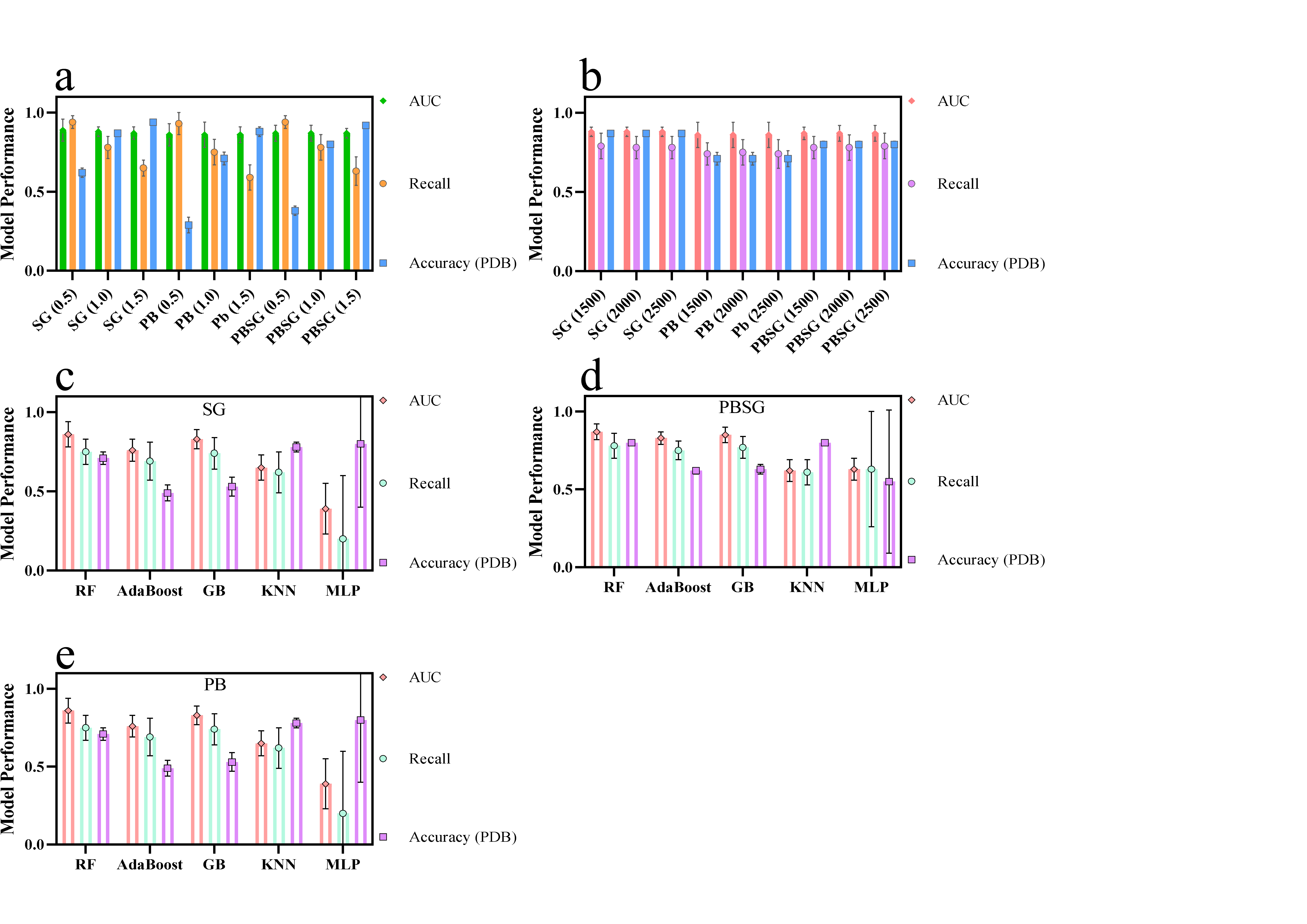


**Fig. S7. Sensitivity analysis of model performance for RNA granule classification across sampling strategies (a), model complexity (b), and machine learning algorithms (c-e).** (a) Performance evaluation of RNA granule classifiers (SG, PB, and PBSG) trained with varying negative-to-positive sample ratios (0.5, 1.0, and 1.5) using negative samples from the human proteome. (b) Assessment of model robustness across different random forest complexities (1500, 2000, and 2500 trees) for SG, PB, and PBSG classifiers. (c-e) Comparative analysis of five machine learning algorithms (random forest (RF), AdaBoost, gradient boosting (GB), k-nearest neighbor (KNN), and multilayer perceptron (MLP)) for SG (c), PBSG (d), and PB (e) classification. Performance metrics include AUC and recall (ten-fold cross-validation), and accuracy on an independent test set of unlikely-LLPS proteins from the Protein Data Bank (PDB) (*N* = 1427).


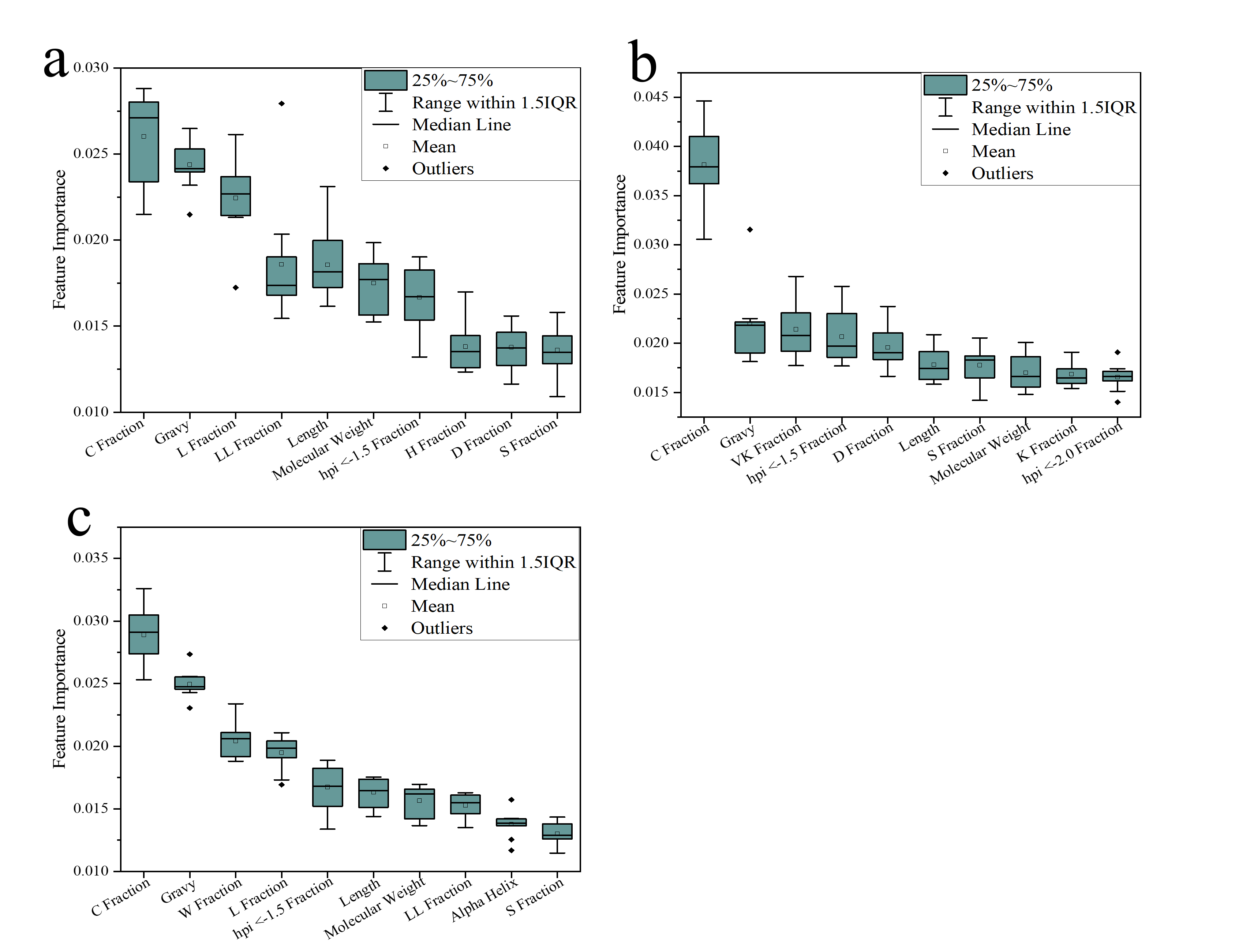


**Fig. S8. Top ten important features of RNA granule classifiers (SG classifier in a and PB classifier in b).** The average feature importance of ten-fold evaluated models was applied to select the top ten most important features in the SG (a) and PB (b) classifiers, respectively. The Gini feature importance scores were used to measure the relative importance of each feature from ten trained random forest models in the ten-fold cross-validation, by using the ‘*feature_importances_*’ function of the *ensemble* module in the *scikit-learn* package (Version: 1.3.0). IQR: interquartile range.


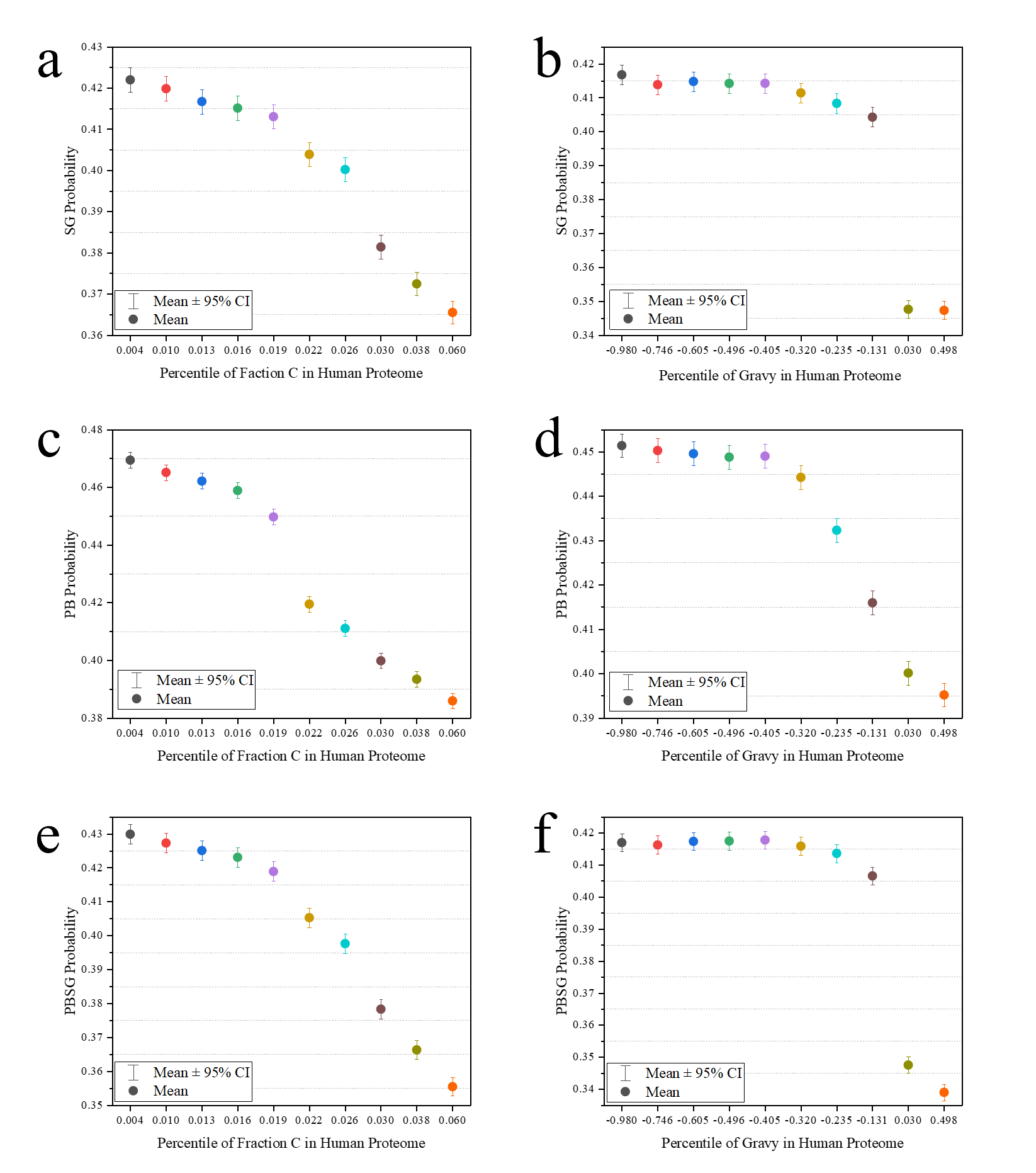
**Fig. S9. Partial dependence shows the relationship between selected key features (fraction C and gravy) with prediction probabilities of SG, PB or PBSG classifiers on the human proteome, respectively.** The partial dependence method was employed to estimate the average prediction probability, considering actual feature values (*i.e.,* 5, 15, 25, 35, 45, 55, 65, 75, 85, and 95 percentiles) of the selected key feature in the whole human proteome, respectively. The partial dependence method assumes the estimated key feature is independent and uncorrelated.


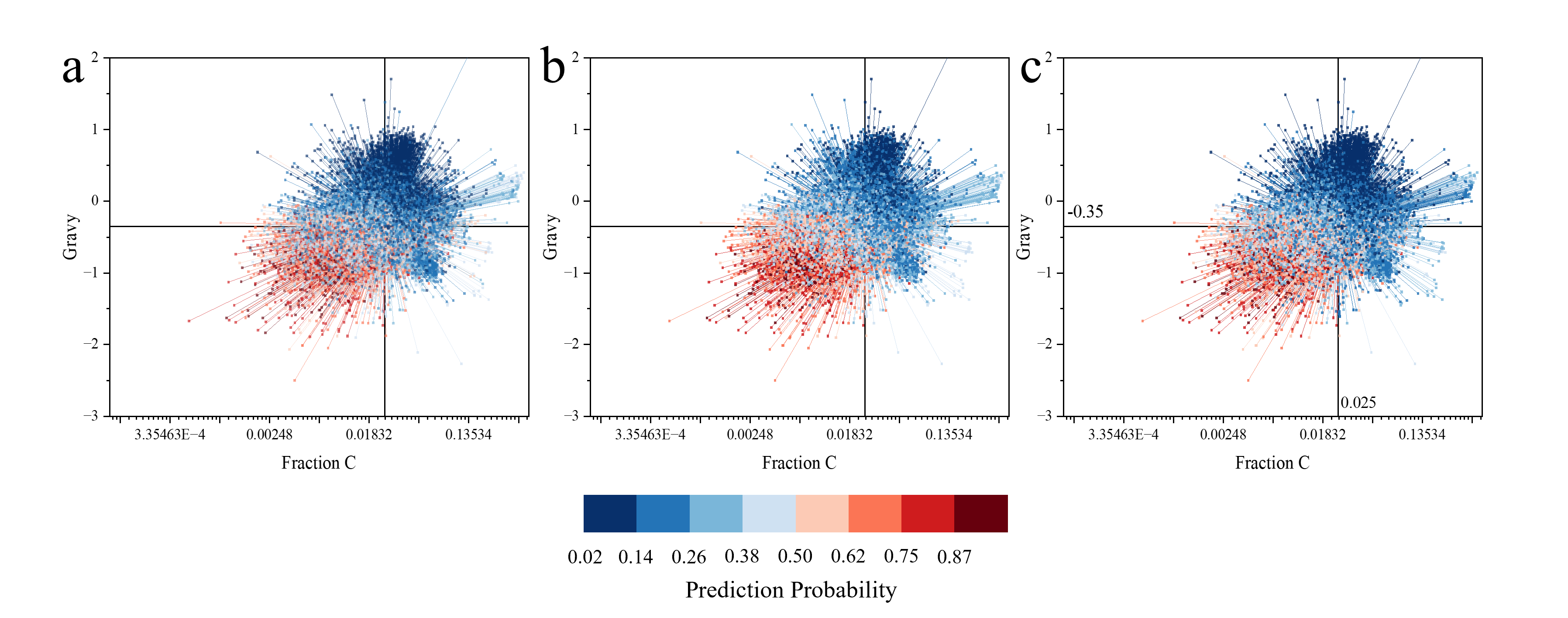


**Fig. S10. The relationship between prediction probabilities and two key features (fraction C and gravy) of the human proteome (the SG model in a, the PB model in b and the PBSG model in c).** The centroid was generated and performed using Origin Pro software.


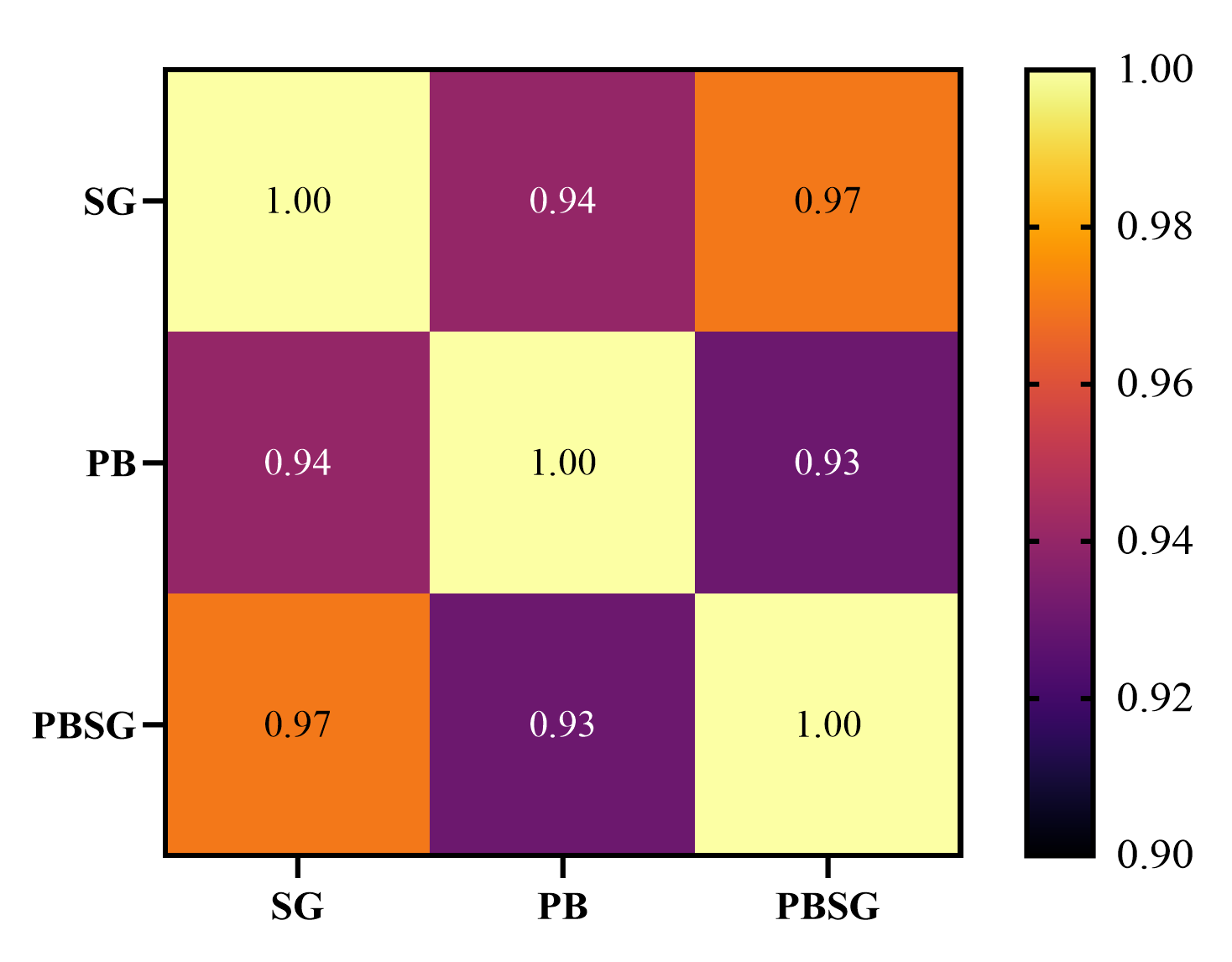


**Fig. S11. Similarity of prediction probabilities of the human proteome (*N* = 20422) across three RNA granule classifiers (*i.e.,* SG, PB and PBSG classifiers).** We selected the PBSG for further analysis based on the high similarities (*i.e.,* correlation coefficient values over 0.93) among the three RNA granule models. Similarity was estimated using the Pearson correlation coefficient.


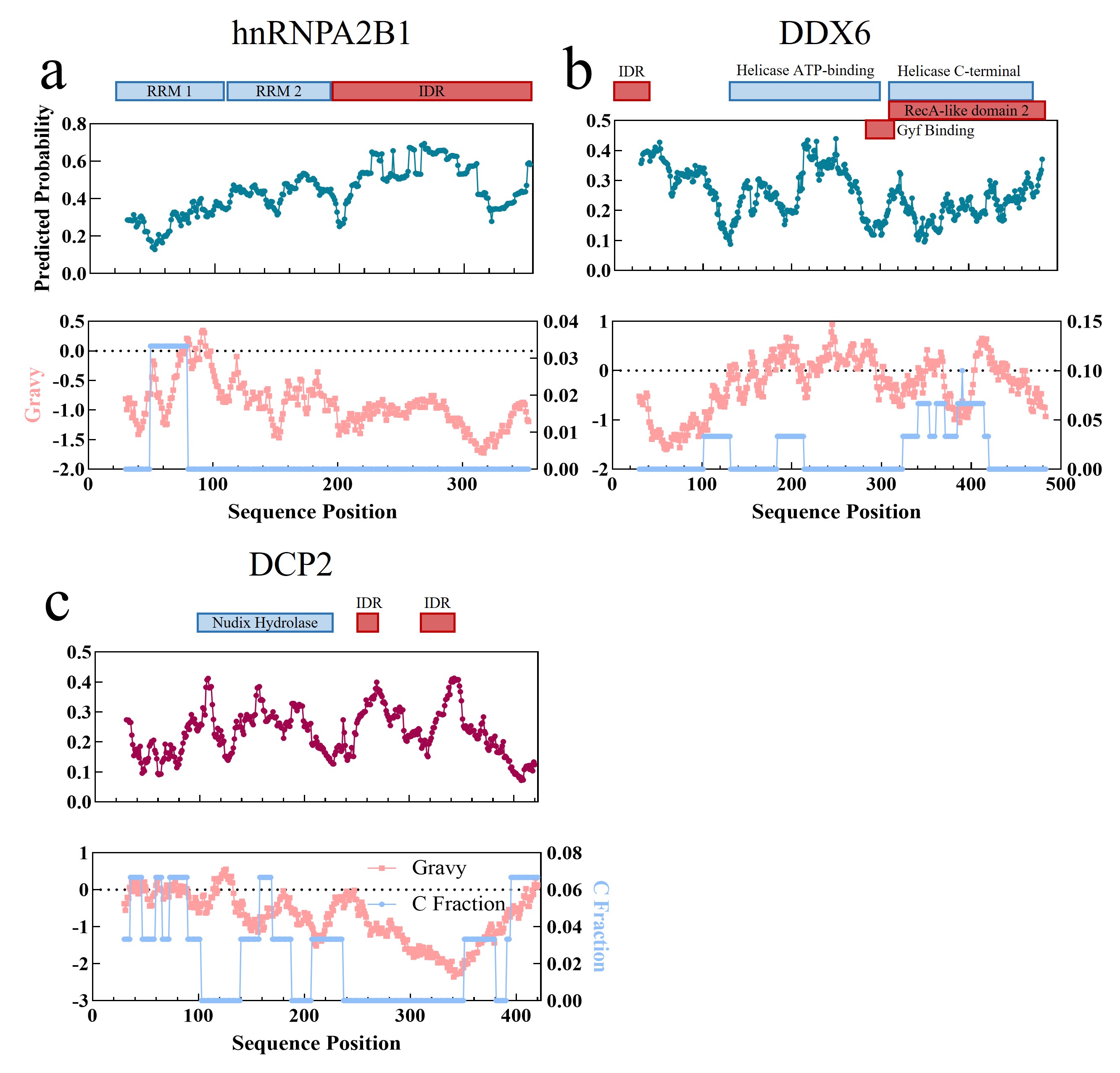


**Fig. S12. Model performance on highly evaluated RNA granule biomarker proteins within sliding windows (30 residues).** The average prediction probability from ten-fold cross-validation of the selected PBSG classifiers was evaluated on highly evaluated RNA granule biomarkers (hnRNPA2B1 in a and DDX6 in b for SG; DCP2 in c for PB) using a sliding window of 30 residues. The average values of the top two features (C fraction and gravy) are shown for these biomarkers. Domain and region information for these proteins was collected from the Uniprot database. RRM1: RNA recognition motif 1. RRM2: RNA recognition motif 2.


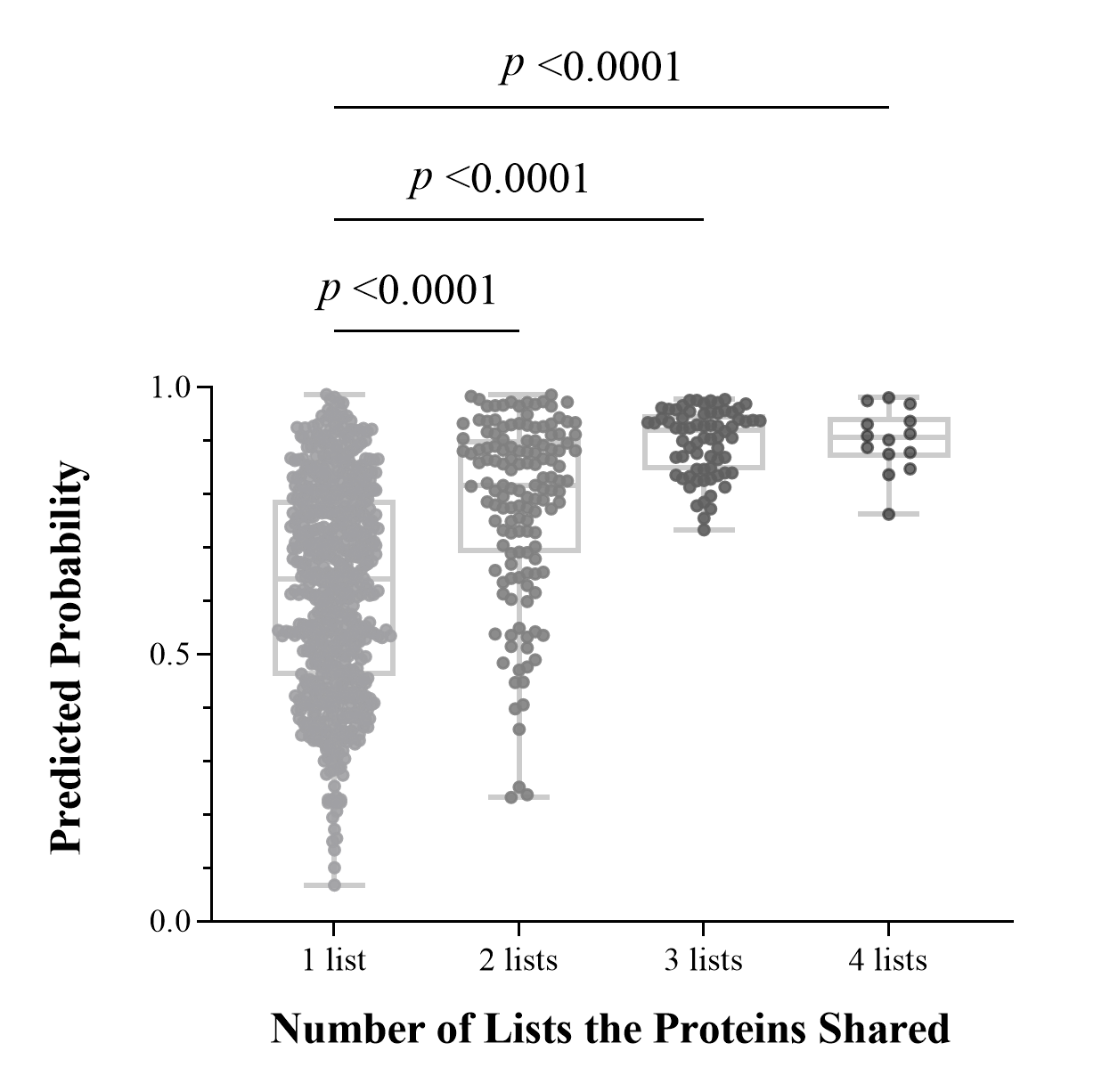


**Fig. S13. Comparison of prediction performance for our RNA granule protein classification model on SG proteins shared across different numbers of four published SG proteome lists.** One-way ANOVA analysis was applied to compare prediction probabilities of proteins shared in only one list, two lists, three lists or four lists by the RNA granule model**.** The number of proteins shared among these SG proteomes is listed in Table S5.


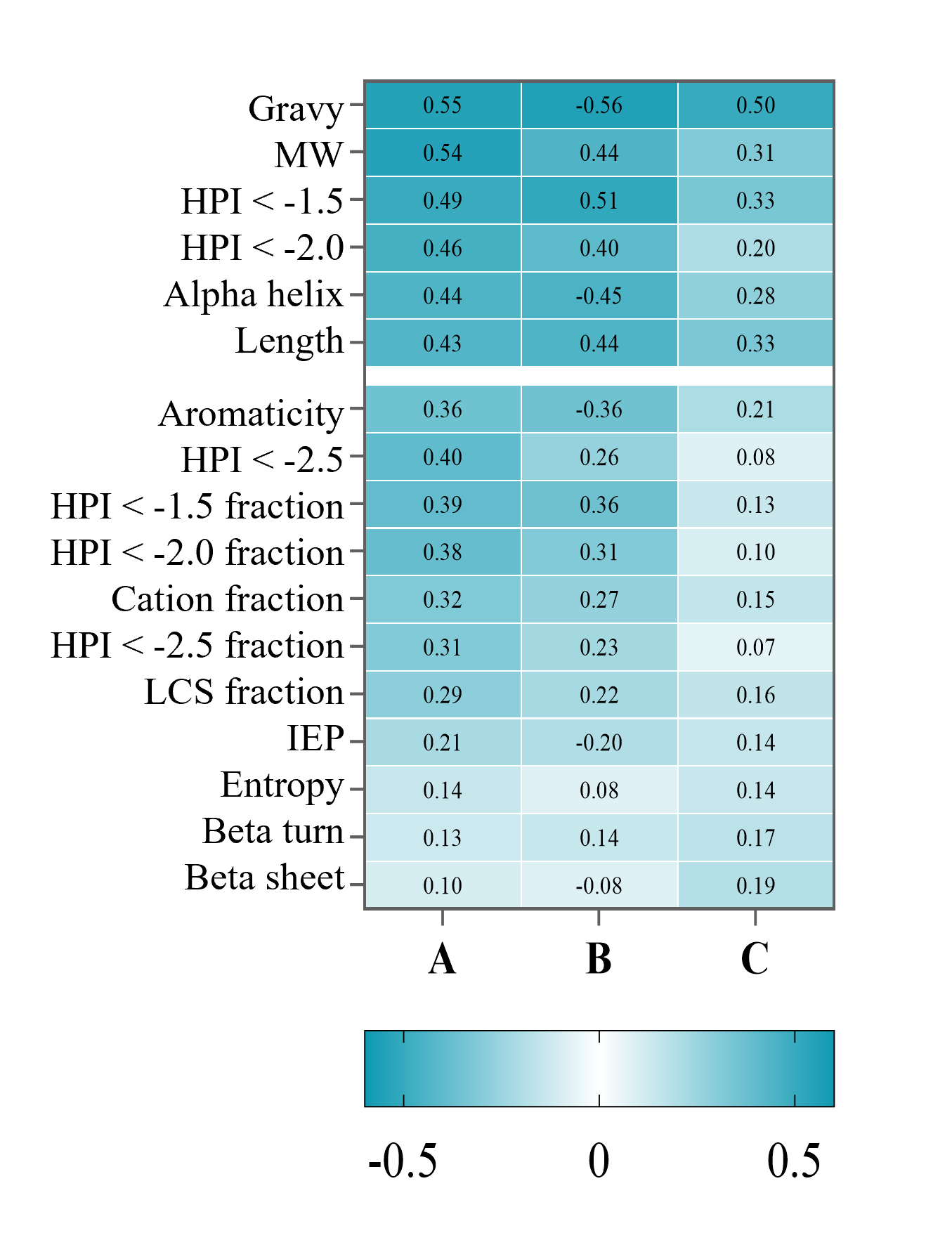


**Fig. S14. Top six important feature selections.** The top six important features were selected based on distance correlation coefficient values (A), Pearson correlation coefficient values (B), and mean feature importance (C). Distance correlation coefficient values and Pearson correlation coefficient values were calculated between the predicted propensities for RNA granule proteins (as predicted by our RNA granule classifiers) and the protein feature values of the human proteome. To simplify comparison, the feature importance of each feature was calculated as the mean feature importance multiplied by 20 in our RNA granule model.


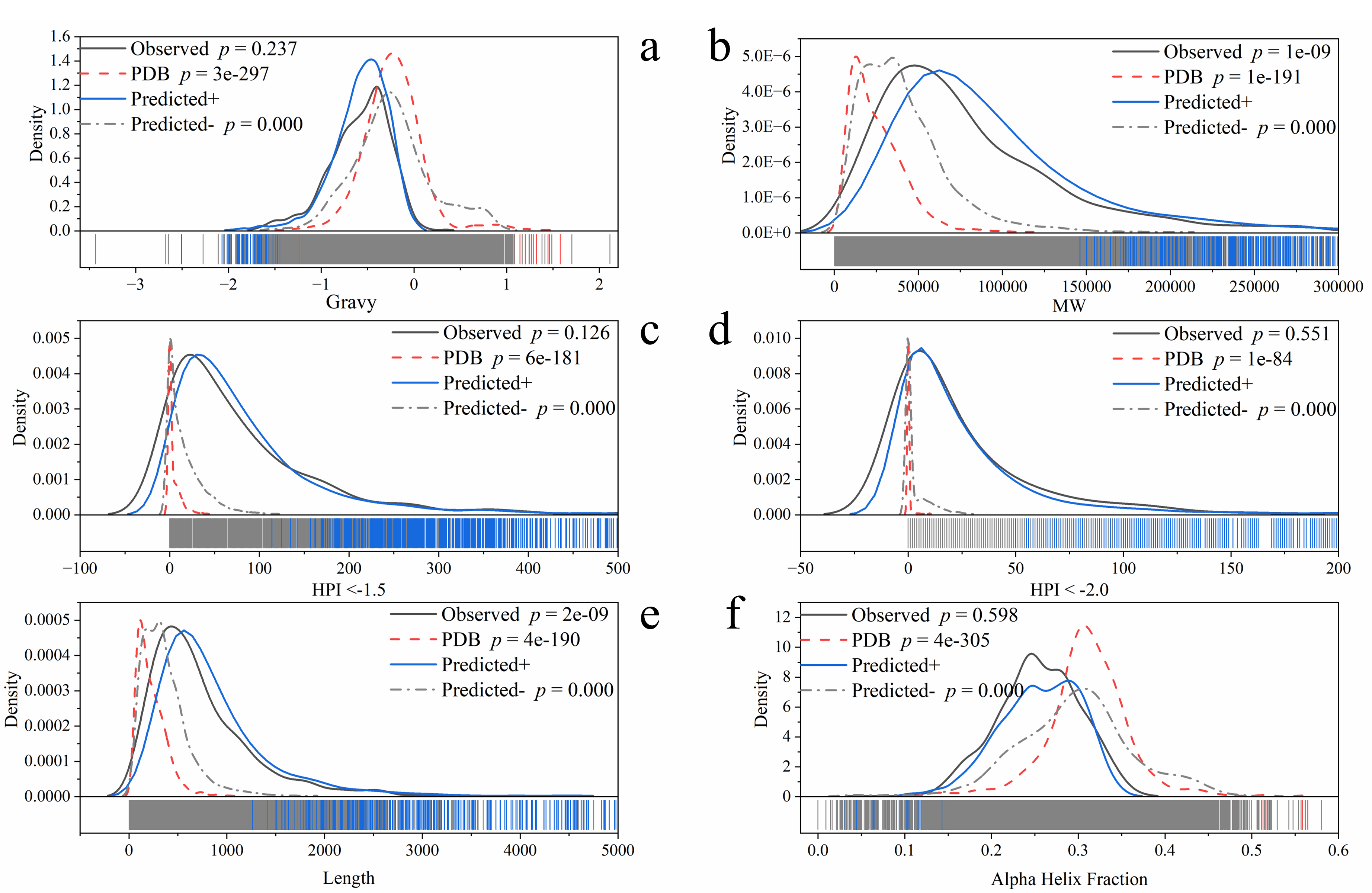


**Fig. S15. Distribution of selected important physicochemical features in observed and predicted RNA granule proteins.** We identified the RNA granule proteome (*N* = 6723) from the human proteome (*N* = 20422) using the selected PBSG classifiers with a probability greater than 0.5. Observed data sets consisted of high-confidence tier 1 or tier 2 proteins (tier 1 for SG, tier 1 for PBSG and tier 1 & 2 for PB, *N* = 951) from the RNAgranuleDB, which were used to train RNA granule classifiers. One-way ANOVA was applied to calculate the *p* values between the predicted RNA granule proteome (‘Predicted+’, probability over 0.5, *N* = 6723) and the observed RNA granule protein candidates (‘Observed’, *N* = 951), unlikely-LLPS PDB proteins (‘PDB’, *N* = 1427), and the predicted non-RNA granule proteome (‘Predicted-’, *N* = 13699). For example, the *p* value for comparing the gravy values of the predicted RNA granule proteome with observed high-confidence RNA granule proteins (tier 1 for SG and PBSG, tier 1&2 for PB) from the database was 0.237 in a.


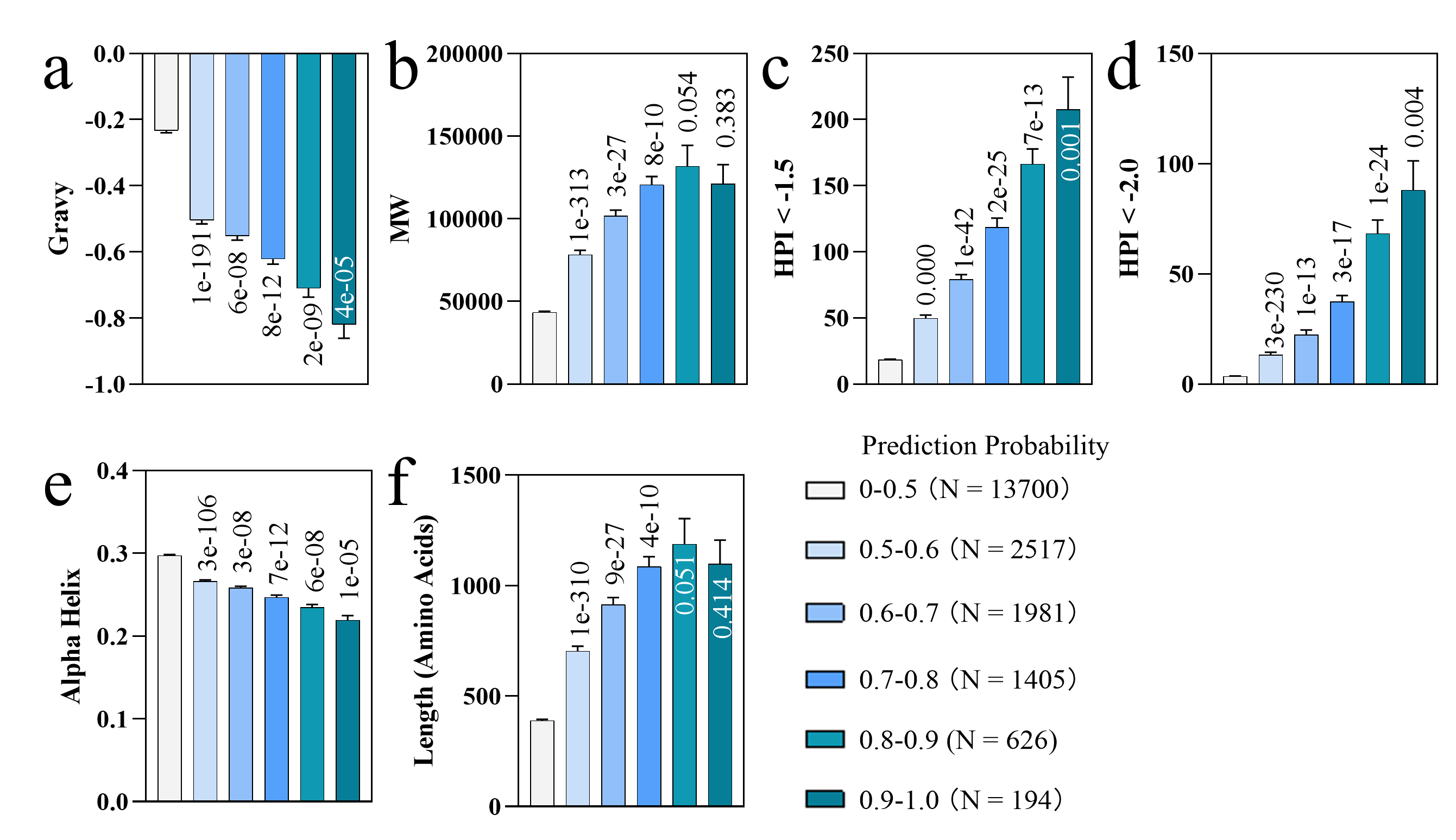


**Fig. S16. Selected important physicochemical features in the identified RNA granule proteome from the human proteome with different prediction probabilities by the selected PBSG classifier.** Bars represent the average value of each feature and error bars represent the 95% confidence interval (CI) of each feature. The *p* value from the one-way ANOVA test between data in the target group and the previous group data (*e.g.,* the *p* value is 1e-191 compared gravy values of proteins with prediction probabilities between 0-0.5 and those with probabilities between 0.5-0.6) is shown on or below the bar.


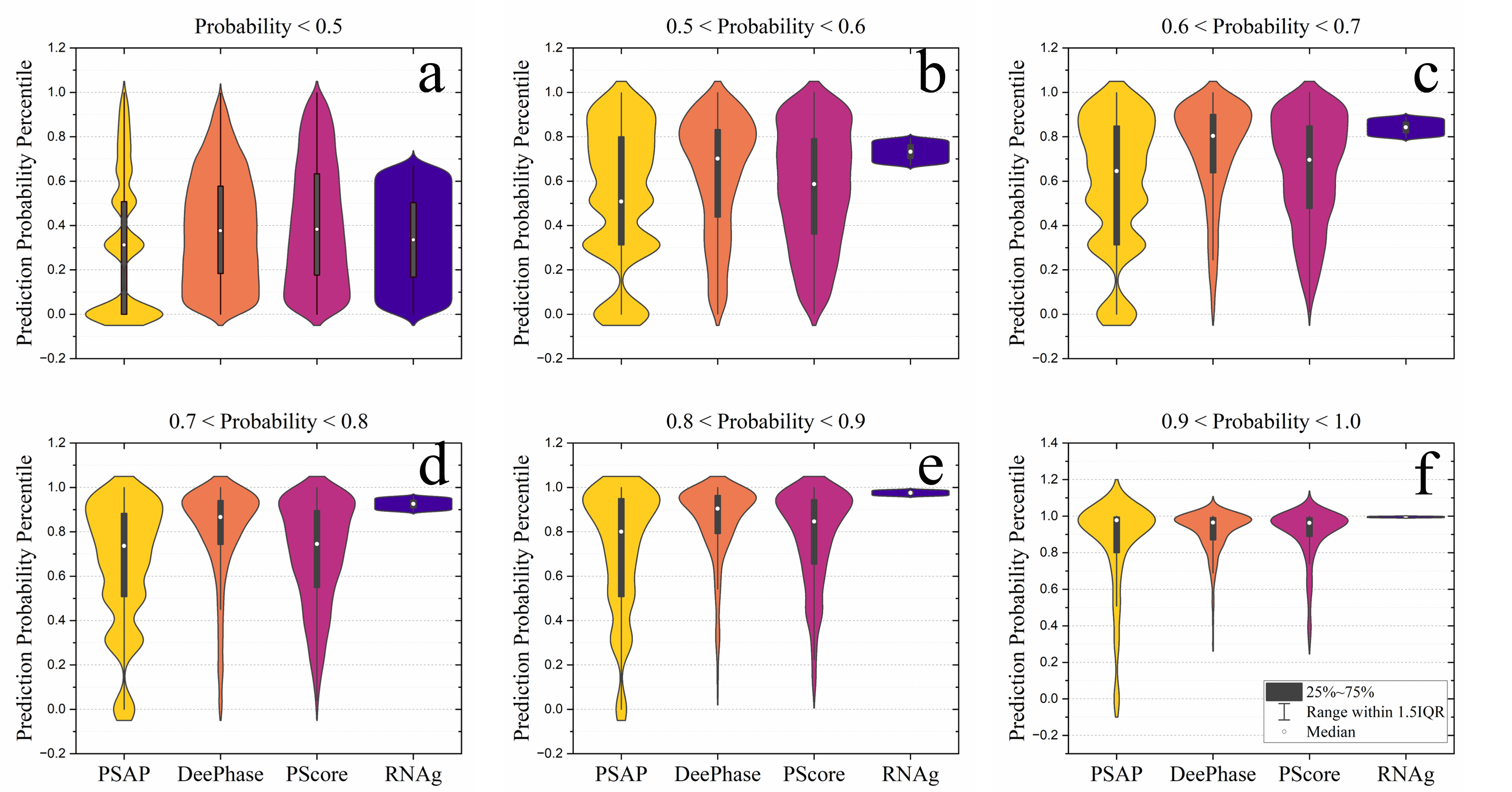


**Fig. S17. The relationship between predicted LLPS propensities and predicted RNA granule propensities in the identified RNA granule proteome (0-0.5 in a, *N* = 10212; 0.5-0.6 in b, *N* = 2043, 0.6-0.7 in c, *N* = 2040; 0.7-0.8 in d, *N* = 2043; 0.8-0.9 in e, *N* = 2041; and 0.9-1.0 in f, *N* = 2042).** We utilized three widely applied LLPS propensity prediction models (*i.e.,* PSAR, DeePhase and PScore) to predict LLPS probabilities for our identified RNA granule proteome. Then, we calculated the average rank percentile values of prediction LLPS scores or RNA granule probabilities to estimate the relative LLPS and RNA granule propensities in the human proteome. RNAg: our RNA granule model**.**

**Fig. S18. Functional enrichment analysis of the observed RNA granule proteins (a, *N* = 473), the overall identified RNA granule proteome (b, *N* = 6723), and the predicted LLPS-prone proteome (c, *N* = 6723).** (a) We collected the overall RNA granule proteins utilized to train our classifiers (*i.e.,* tier 1 PBSG proteins, *N* = 473). (b) We identified the overall RNA granule proteome based on prediction probabilities (over 0.5, *N* = 6723) from our RNA granule protein model. Then, we completed the functional enrichment analysis on Gene Ontology biological process (GOBP), GO cellular component (GOCC), GO molecular function (GOMF), KEGG and domains of observed RNA granule protein candidates, predicted RNA granule proteome, and the predicted LLPS proteome, respectively. Shared terms among the top 20 significantly enriched terms (based on their *p* values) for the observed (*N* = 473) and predicted high-confidence RNA granule proteins (*N* = 2225) are shown in Fig. 3 of the main manuscript. (c) We used three classic and widely applied LLPS prediction models (PSAR, DeePhase and PScore) to select LLPS-prone proteins (*N* = 6723, the same number as the overall predicted RNA granule proteins) based on the average rank percentile values of prediction LLPS scores. The Enrichr platform was applied for the enrichment analysis.


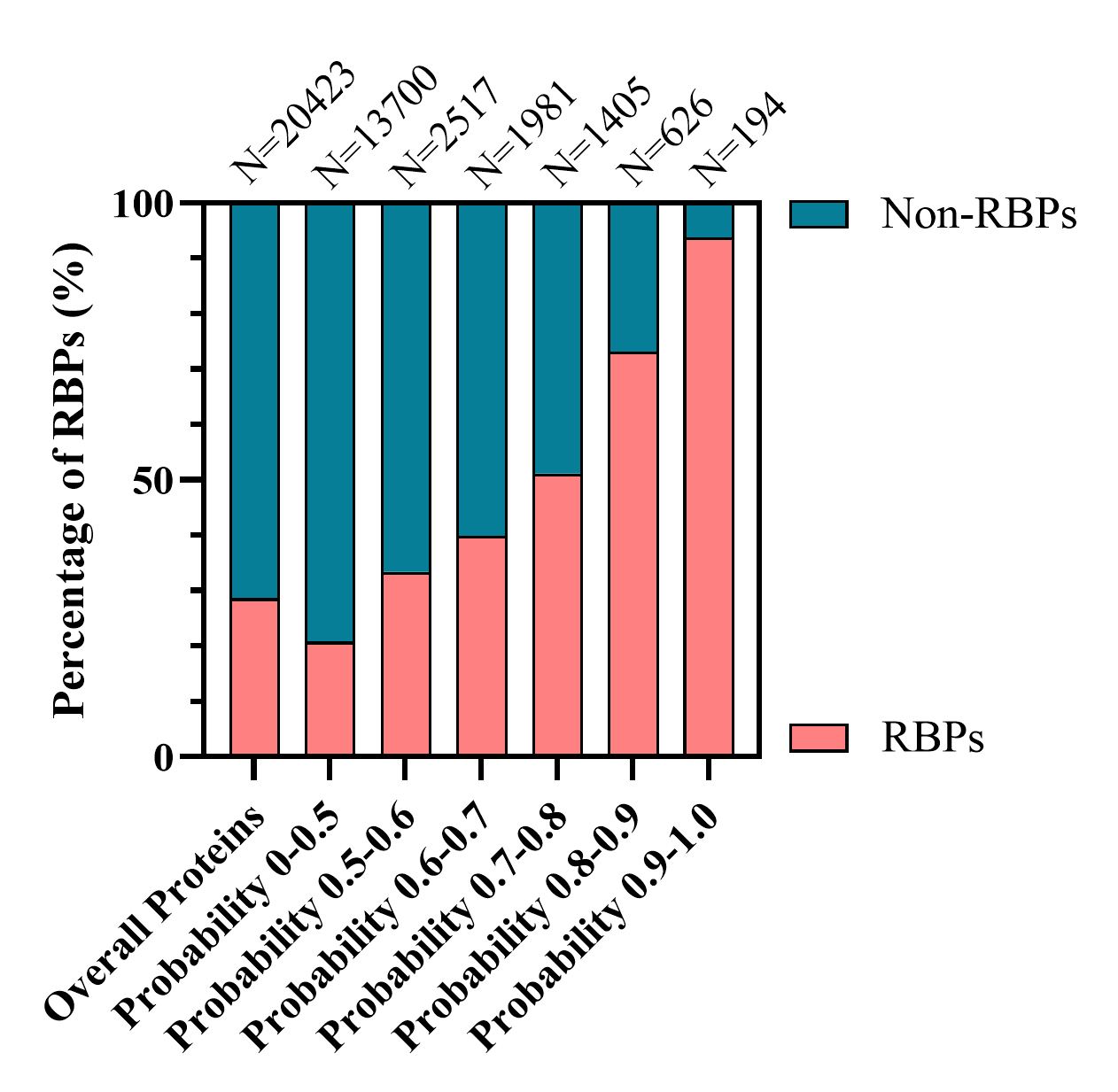
**Fig. S19. Distribution of RBPs in human proteins with different RNA granule protein propensities (overall human proteome, 0-0.5, 0.5-0.6, 0.6-0.7, 0.7-0.8, 0.8-0.9, and 0.9-1.0) predicted by our RNA granule model.** We collected human RBPs (*N* = 6163) from the RBPbase database and applied our RNA granule protein model to predict the RNA granule probabilities of each protein in the human proteome. Then, we calculated the percentage of RBPs in each group of proteins with varying RNA granule protein propensities.


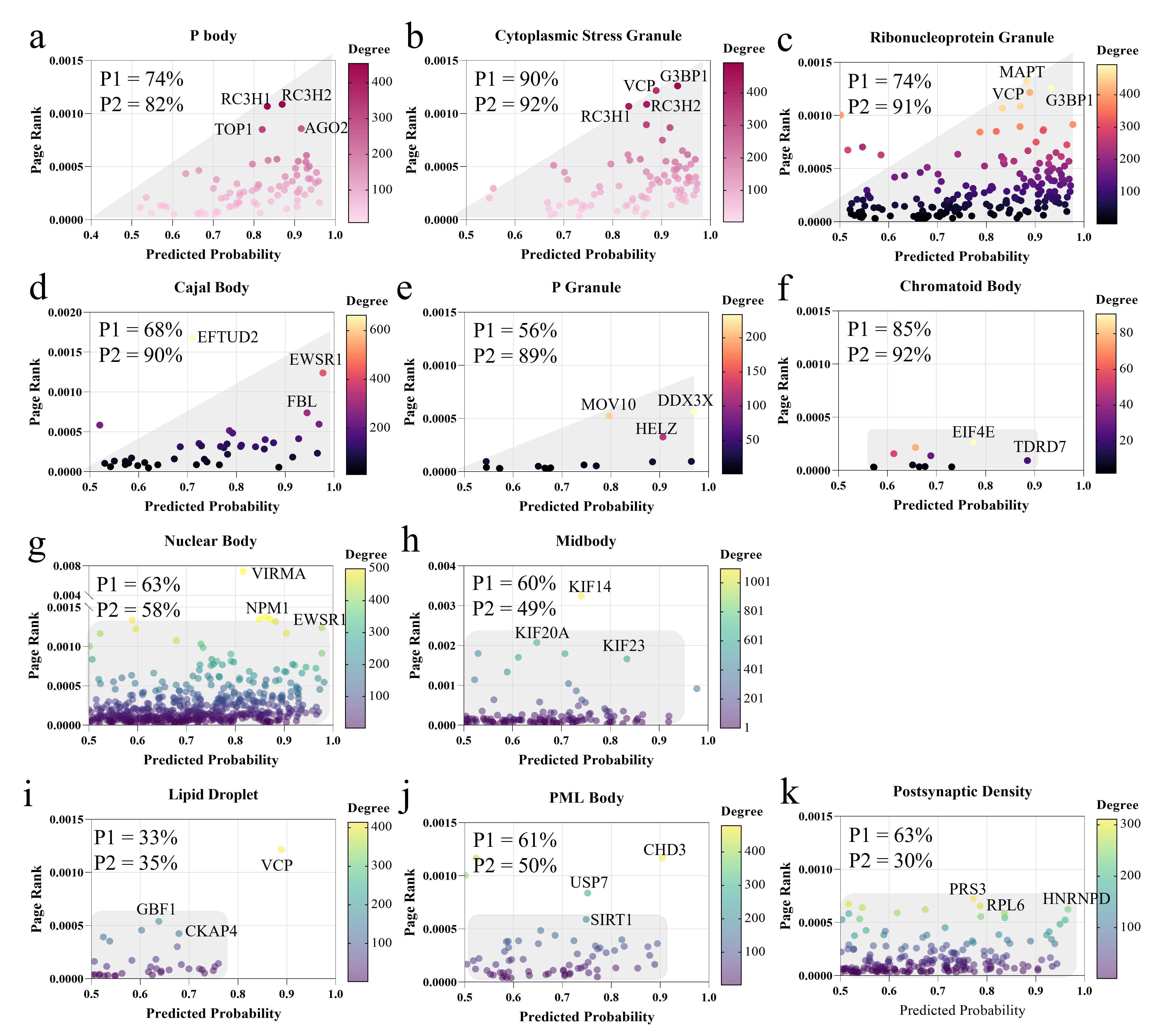
**Fig. S20.** **Evaluation of biomolecular and community grammars of our model on typical RNA granules and other biomolecular condensates.** We applied the predicted probability of each protein in our model to assess its likelihood of being a component of RNA granules (*i.e.,* PB or SG). The PageRank value evaluates the centrality of each protein in the identified RNA granule proteome community according to our model. We collected typically classified RNA granules, including PB (a), SG (b), ribonucleoprotein granule (c), Cajal body (d), P granule (e) and Chromatoid body (f), nuclear body (g), Midbody (h) and other biomolecular condensates not typically classified as RNA granules, including Lipid droplet (i), PML body (j) and postsynaptic density (k) from the QuickGO database. We applied the predicted probabilities from our PBSG model and PageRank values for the collected components to evaluate biomolecular and community grammars in diverse RNA granules. P1: the percentage of protein components with predicted probabilities over 0.5 by our model in the total number of protein components of each RNA granule or biomolecular condensate. P2: the percentage of protein components identified as RBPs in the total number of protein components of each RNA granule or biomolecular condensate. Protein components of all biomolecular condensates were collected from the QuickGO database (Version: 2023-10-06; access date: Oct. 2023). RBPs were collected from the RBPbase database (version: v0.2.1 alpha; access date: Oct. 2023).


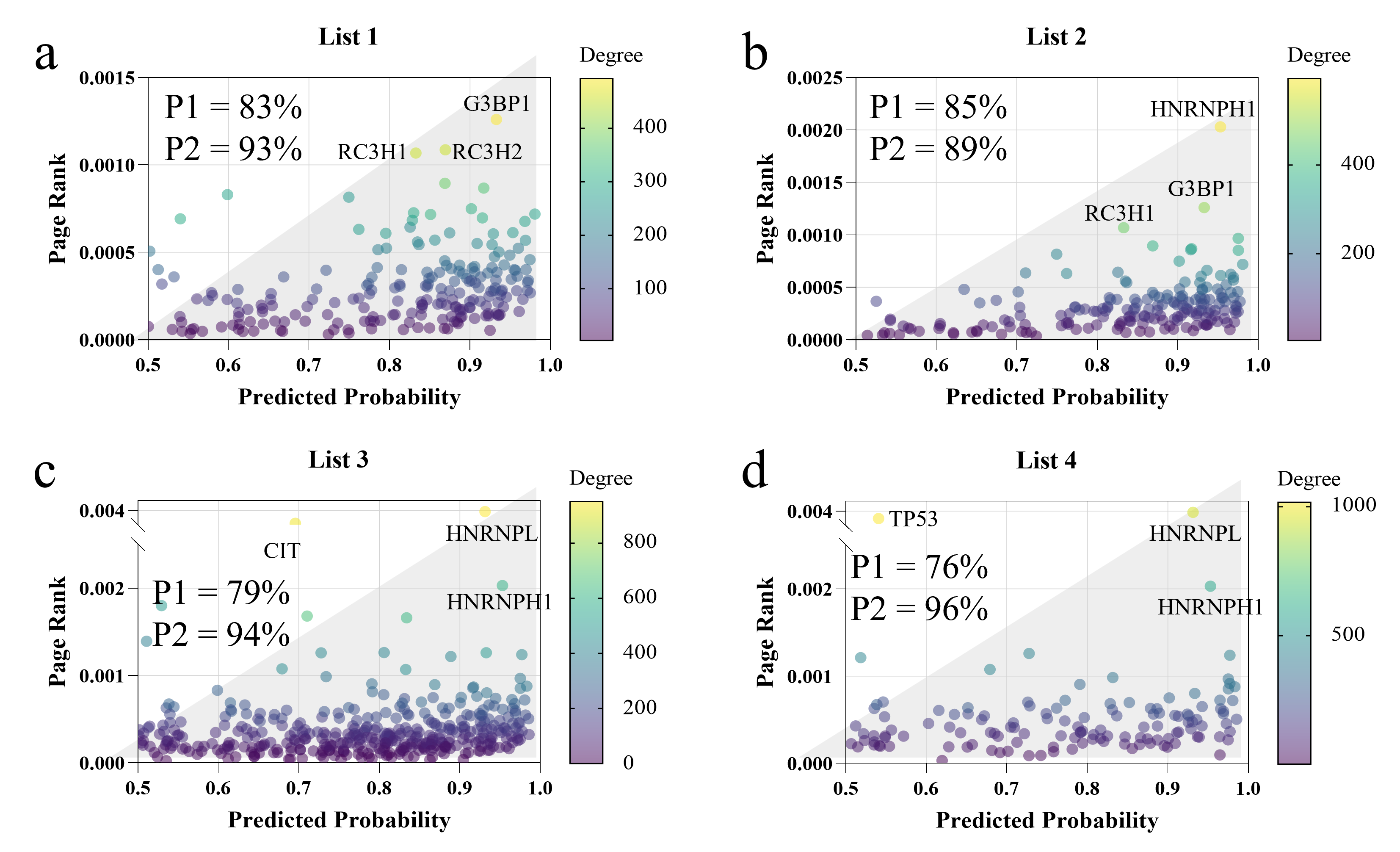
**Fig. S21.** **Evaluation of biomolecular and community grammars in four experimental SG proteome lists.** We applied the predicted probability of each protein in our model to assess its likelihood of being a component of RNA granules. The PageRank value evaluates the centrality of each protein in the identified RNA granule proteome community. Four experimental SG proteome lists were collected (list 1: *N* = 253, list 2: *N* = 221, list 3: *N* = 486, and list 4: *N* = 172). We used the predicted probabilities from our model and the PageRank values of the collected components to evaluate the biomolecular and community grammars of the SG proteomes. P1: the percentage of protein components with predicted probabilities over 0.5 by our model in the total number of protein components in each SG proteome. P2: the percentage of protein components identified as RBPs in the total number of protein components in each SG proteome. The RBPs were collected from the RBPbase database (version: v0.2.1 alpha; access date: Oct. 2023).


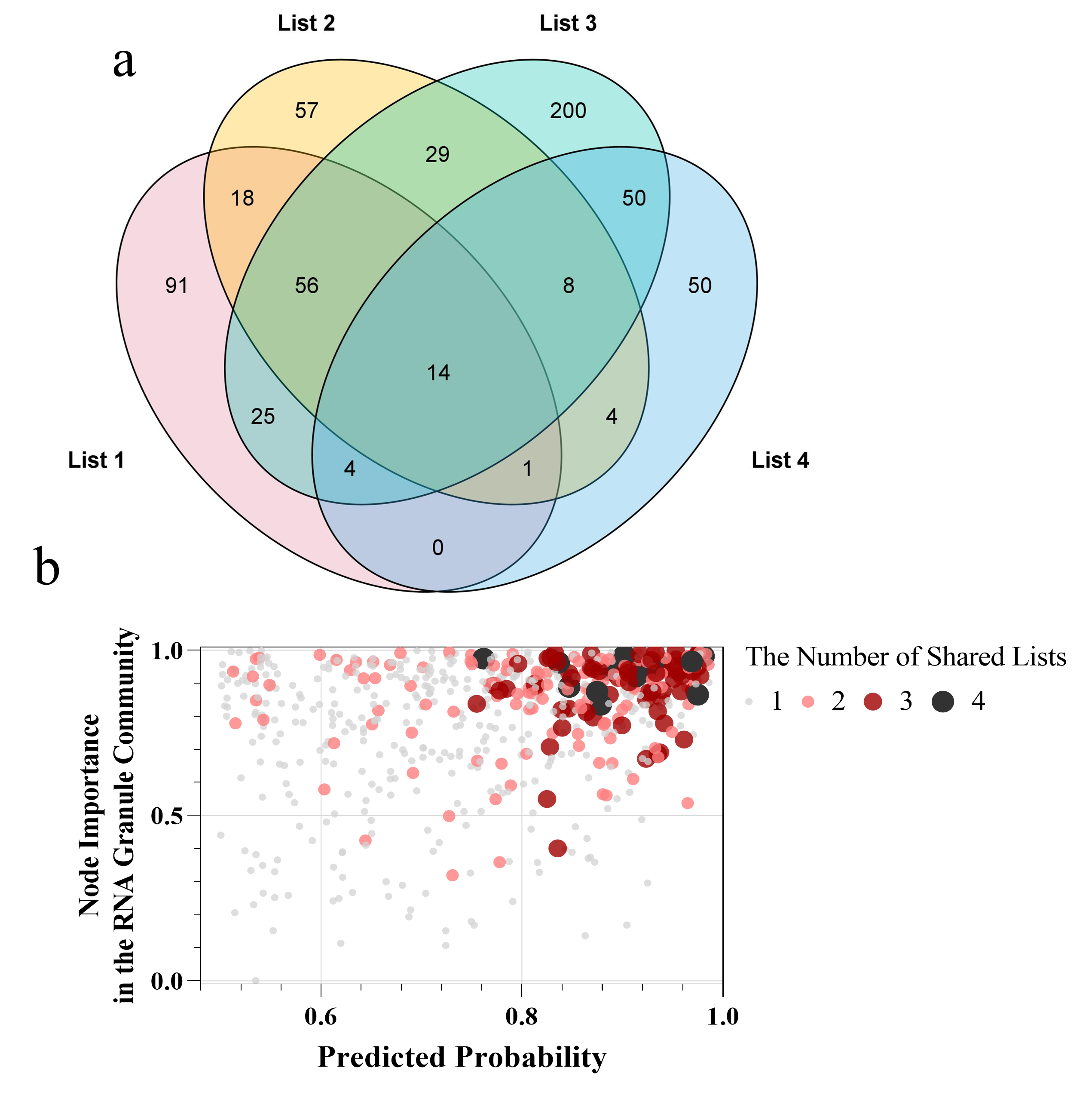


**Fig. S22.** **Relationship between predicted probabilities from our model, centrality in the identified RNA granule protein protein-protein interaction (PPI) network, and popularity of proteins across four SG proteomes collected from publications.** The Venn plot (a) illustrates the number of proteins shared among the four different SG proteome lists. The dot plot (b) illustrates the distribution of the most popular SG proteins with varying centrality and predicted probabilities across the four collected SG proteomes.

**Fig. S23.** **Relationship between node importance in the RNA granule proteome PPI network and molecular functions of predicted RNA granule proteins.** We evaluated the potential molecular functions by applying the top ten GO molecular functions (*p* value < 0.05) for proteins with different levels of node importance. The percentile rank of PageRank was provided to measure each protein’s importance in the RNA granule proteome PPI network (*N* = 6600). Enrichment analysis was performed using the Enrichr platform.


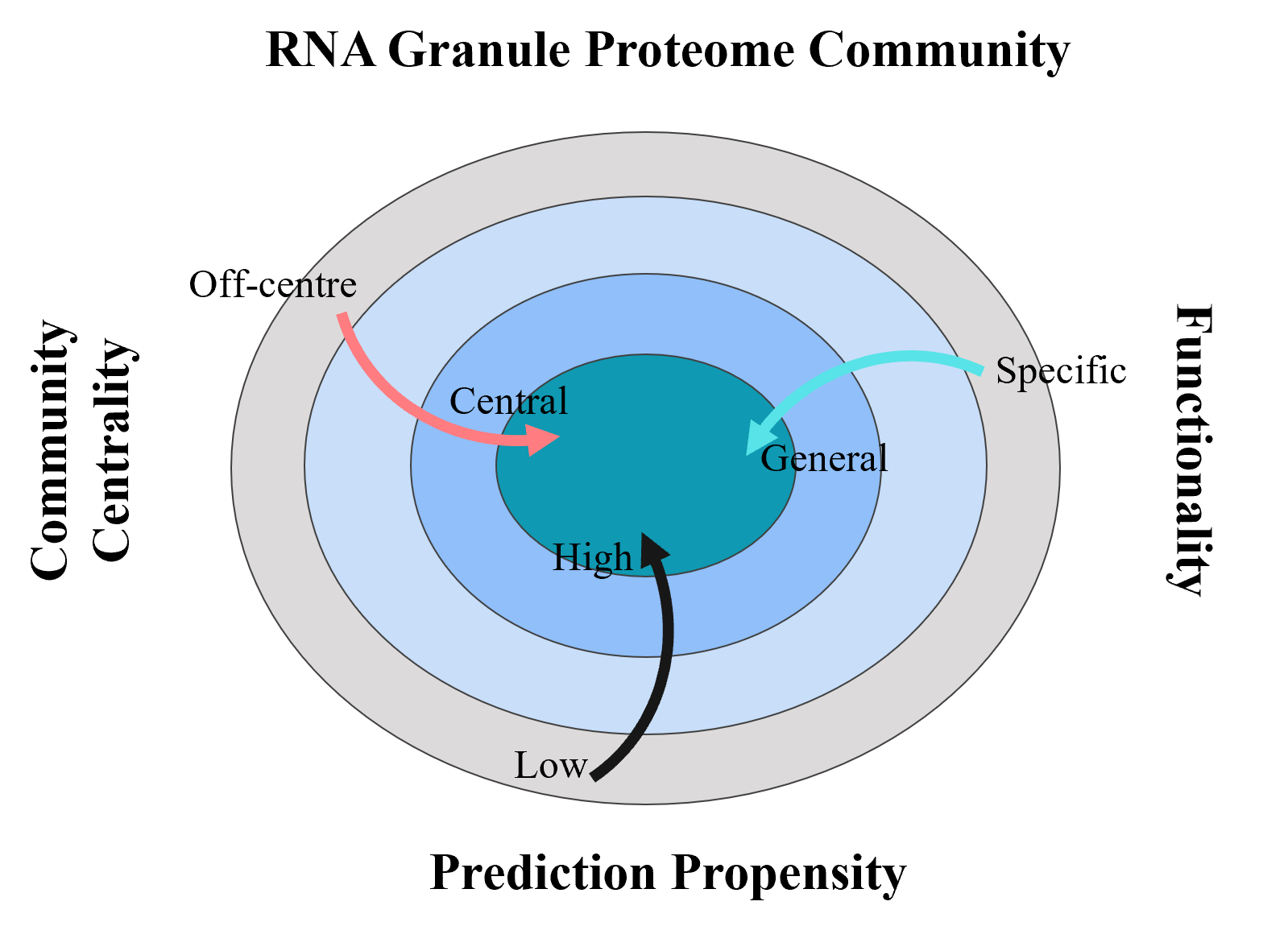
**Fig. S24.** **Biomolecular and community grammar in the predicted RNA granule proteome.** Community centrality represents the node importance of each protein in the RNA granule proteome PPI community (Figure 4b-h in the main manuscript). The prediction probability represents the likelihood of each protein being an RNA granule protein, as predicted by our RNA granule model. The relationship between protein centrality and biological implications (*i.e.,* central proteins in the community tend to contribute to the main and general characteristic of RNA granule functionality) is shown in Fig. S23.


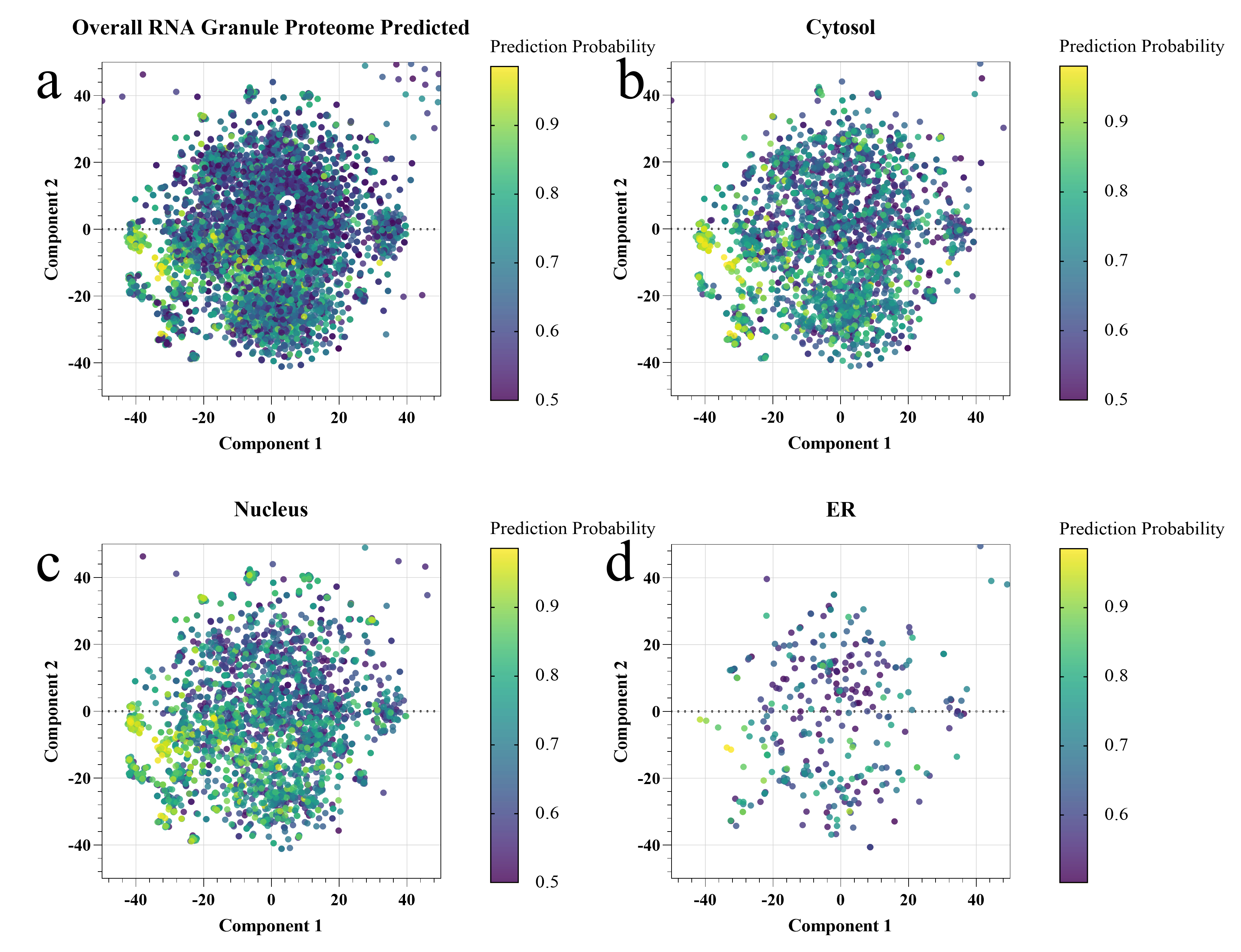


**Fig. S25.** **Visualization of the identified RNA granule proteome in the whole cell, cytosol, nucleus and ER using t-distributed Stochastic Neighbor Embedding (t-SNE).** We visualized overall identified RNA granule proteome PPI network (*N* = 6600) into a 2-dimensional map and collected the locations of each protein in the overall map (*i.e.,* the component 1 and component 2 value) using the t-SNE method. According to the QuickGO database, we collected predicted RNA granule proteins located in the whole cell (a, *N* = 6600), cytosol (b, GO ID: 0005829, *N* = 2652), nucleus (c, GO ID: 0005634, *N* = 2752) and ER (d, GO ID: 0005783, *N* = 325). We visualized the locations of protein components in the whole cell (a), cytosol (b), nucleus (c) and ER (d) using the collected protein locations in the overall map. ER, endoplasmic reticulum.


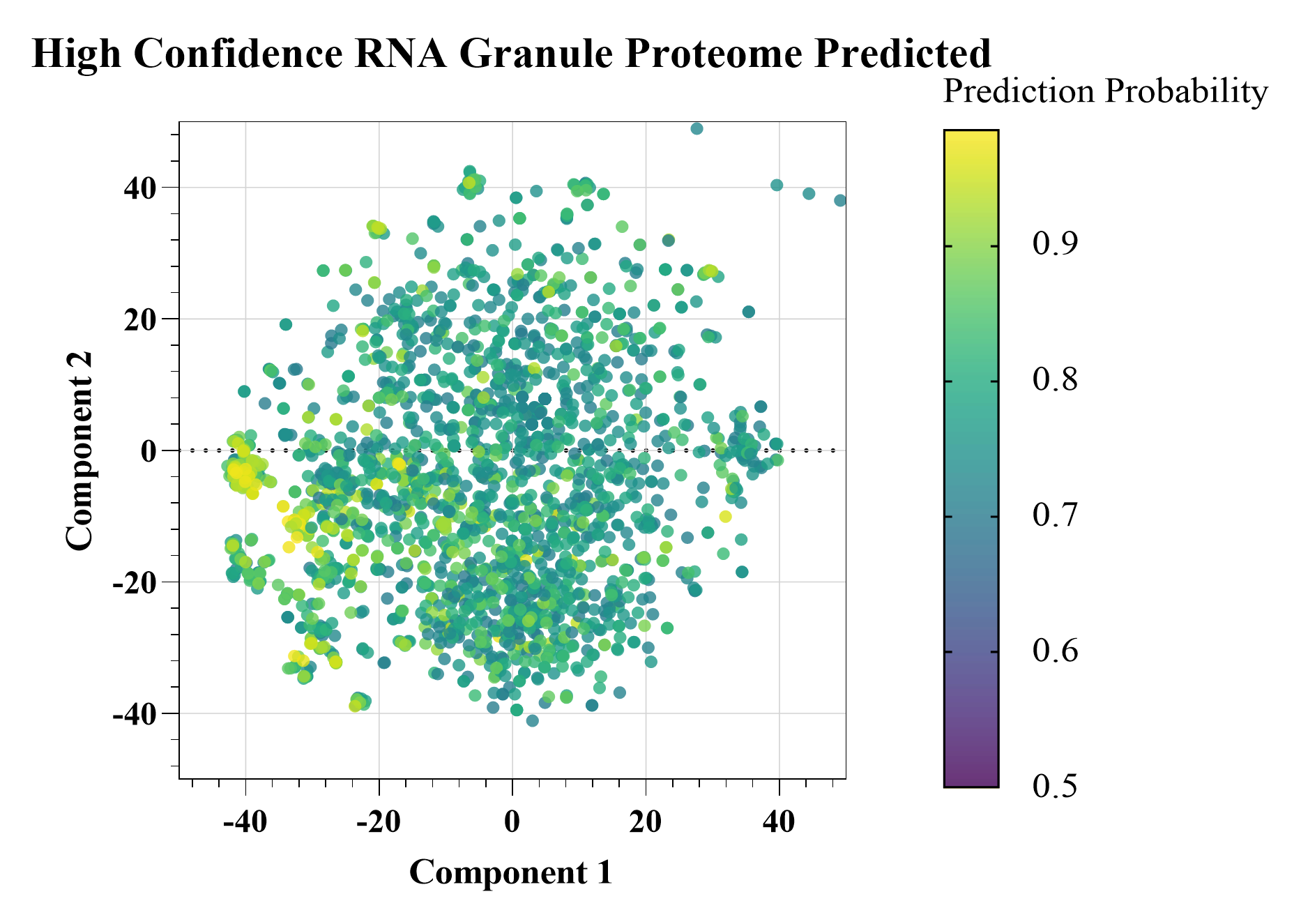
**Fig. S26.** **Visualization of the predicted high-confidence (predicted probabilities ≥ 0.7) RNA granule proteome using t-SNE.** We visualized the identified high-confidence (predicted probabilities ≥ 0.7) RNA granule proteome PPI network (*N* = 2194) into a 2-dimensional map and collected the locations of each protein in the overall map (*i.e.,* the component 1 and component 2 values, *N* = 6600).


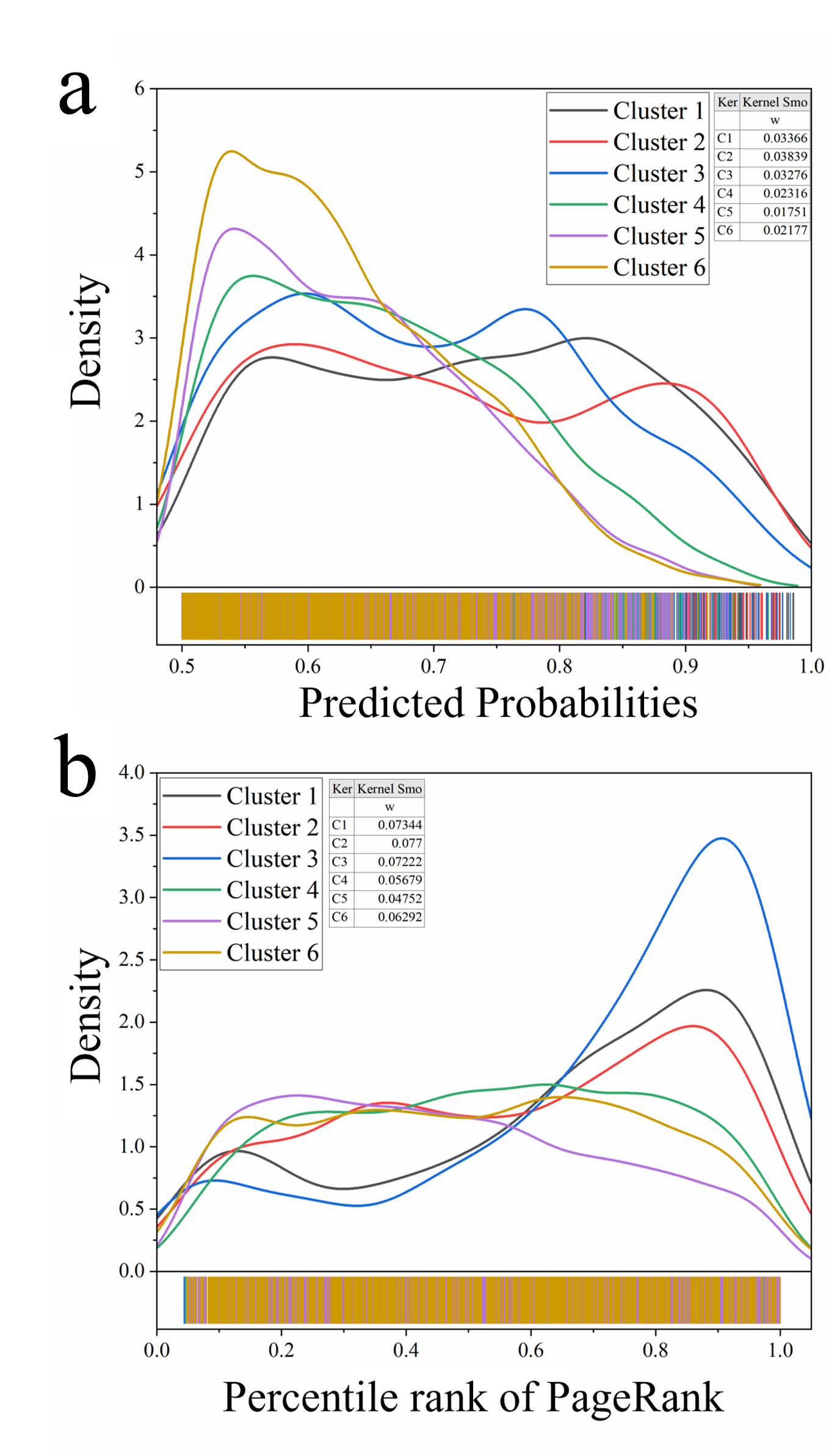
**Fig. S27.** **Distribution of prediction probabilities and percentile ranks of PageRank values for each protein in the RNA granule community (*N* = 6600) across six identified clusters.** Kernel density plots were used to visualize the distribution of predicted probabilities (a) and percentile ranks of PageRank values (b) of proteins in each extracted cluster. The kernel density plots were generated by the OriginPro 2023b software (Learning Edition).

**Fig. S28.** **GO enrichment analysis of six extracted clusters from the overall RNA granule proteome PPI community.** We evaluated the top ten biological processes significantly enriched (*p* value < 0.05) by proteins within different clusters. We applied the Enrichr platform to perform the enrichment analysis.

**Fig. S29.** **Cluster identification in the high-confidence predicted RNA granule proteome PPI network (Cluster 1-, Cluster 2- and Cluster 3- with prediction probabilities ≥ 0.7) (*N* = 2194).** (a) The high-confidence predicted RNA granule proteins were extracted from the six communities detected in the overall RNA granule proteome PPI network (Fig. 5a in the main manuscript). High-confidence Cluster 1-: 331 proteins, average prediction probability of 0.83, and average percentile rank of PageRank values of 0.72. Hich confidence Cluster 2-: 193 proteins, average prediction probability of 0.84, and average percentile rank of PageRank values of 0.70. High-confidence Cluster 3-: 239 proteins, average prediction probability of 0.81, and average percentile rank of PageRank values of 0.75. High-confidence Cluster 4-: 420 proteins, average prediction probability of 0.78, and average percentile rank of PageRank values of 0.60. High-confidence Cluster 5-: 768 proteins, average prediction probability of 0.77, and average percentile rank of PageRank values of 0.52. High-confidence Cluster 6-: 192 proteins, average prediction probability of 0.77, and average percentile rank of PageRank values of 0.57. (b&c) Kernel density plots were used to visualize the distribution of predicted probabilities (b) and percentile ranks of PageRank values (c) of proteins in each extracted cluster. The red circle: Cluster 1. The blue circle: Cluster 2. The green circle: Cluster 3.

**Fig. S30.** **GO enrichment analysis of the three detected high-confidence clusters (Cluster 1-, Cluster 2-, and Cluster 3-) with proteins having predicted probabilities ≥ 0.7.** We evaluated the top ten biological processes significantly enriched (*p* value < 0.05) by proteins within the high-confidence clusters. We used the Enrichr platform to perform the enrichment analysis.


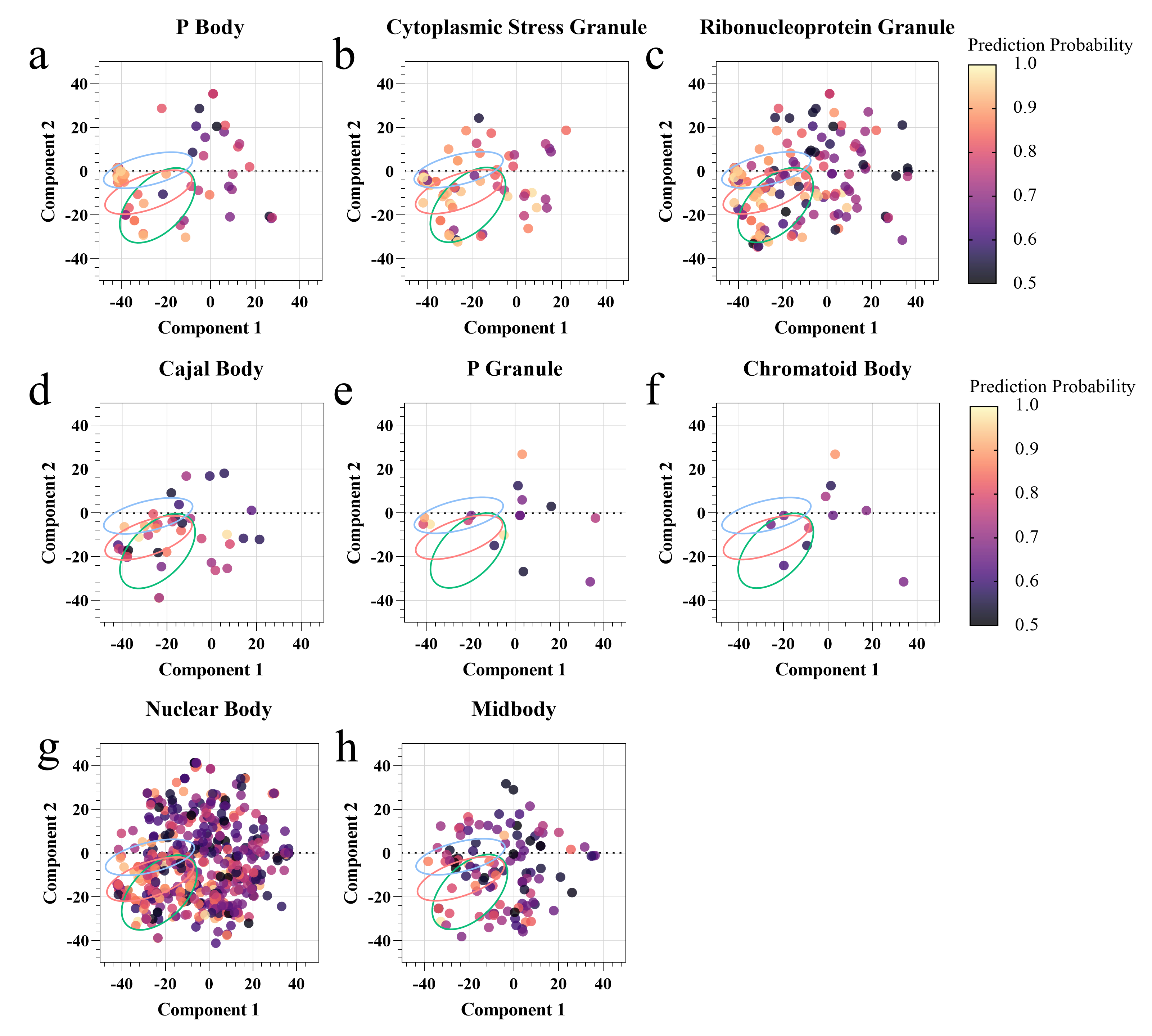


**Fig. S31.** **Visualization of the selected Cluster 1, Cluster 2 and Cluster 3 in the collected typical RNA granules using t-SNE.** (a) We visualized the overall identified RNA granule proteome PPI network (*N* = 6600) in a 2-dimensional map and collected the locations of each protein in the overall map (*i.e.,* component 1 and component 2 values). We collected and visualized the locations of proteins from typically classified RNA granules, including PB (a), SG (b), ribonucleoprotein granule (c), Cajal body (d), P granule (e), Chromatoid body (f), nuclear body (g) and Midbody (h) using the collected protein locations in the overall map. The protein components of each typical RNA granule were collected from the QuickGO database (access date: Oct. 2023), as shown in **Table S3**. The red circle: Cluster 1. The blue circle: Cluster 2. The green circle: Cluster 3.

**Fig. S32.** **Visualization of the selected Cluster 1, Cluster 2 and Cluster 3 in the collected non-RNA granules using t-SNE.** (a) We visualized the overall identified RNA granule proteome PPI network (*N* = 6600) in a 2-dimensional map and collected the locations of each protein in the overall map (*i.e.,* component 1 and component 2 values). We collected and visualized the locations of proteins from typically classified non-RNA granules, including lipid droplet (a), PML body (b), postsynaptic density (c) using the collected protein locations in the overall map. The protein components of each typical non-RNA granule were collected from the QuickGO database (access date: Oct. 2023), as shown in **Table S3**. The red circle: Cluster 1. The blue circle: Cluster 2. The green circle: Cluster 3.


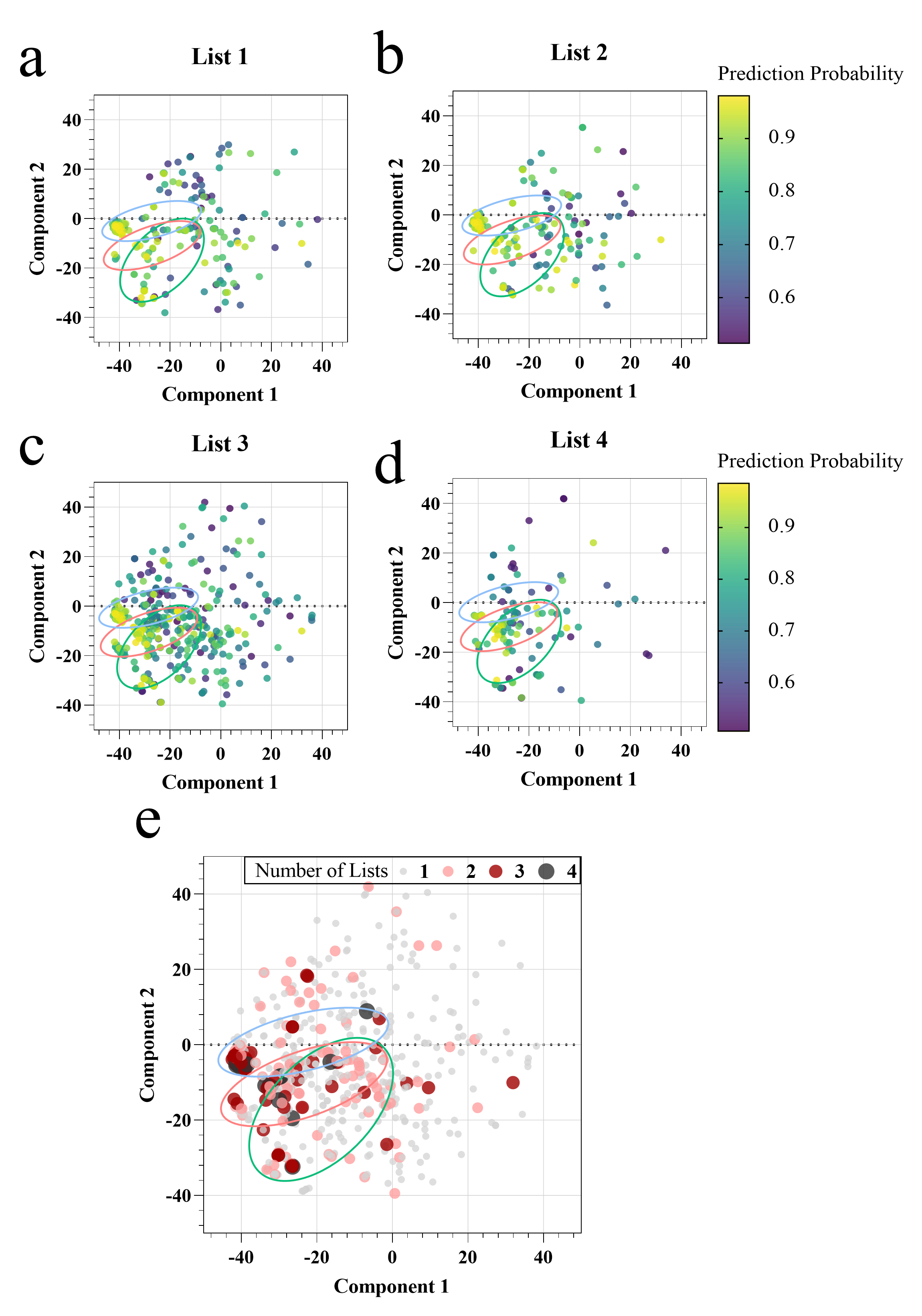


**Fig. S33.** **Visualization of the selected Cluster 1, Cluster 2 and Cluster 3 in SG proteome lists using t-SNE.** We collected four experimental SG proteome lists (List 1: *N* = 253; List 2: *N* = 221; List 3: *N* = 486; List 4: *N* = 172). We visualized the overall identified RNA granule proteome PPI network (*N* = 6600) in a 2-dimensional map and collected the locations of each protein in the overall map (*i.e.,* the component 1 and component 2 value). We visualized the locations of protein components in List 1 (a), List 2 (b), List 3 (c) and List 4 (d) using the collected protein locations of each protein in the overall map. The red circle: Cluster 1. The blue circle: Cluster 2. The green circle: Cluster 3.


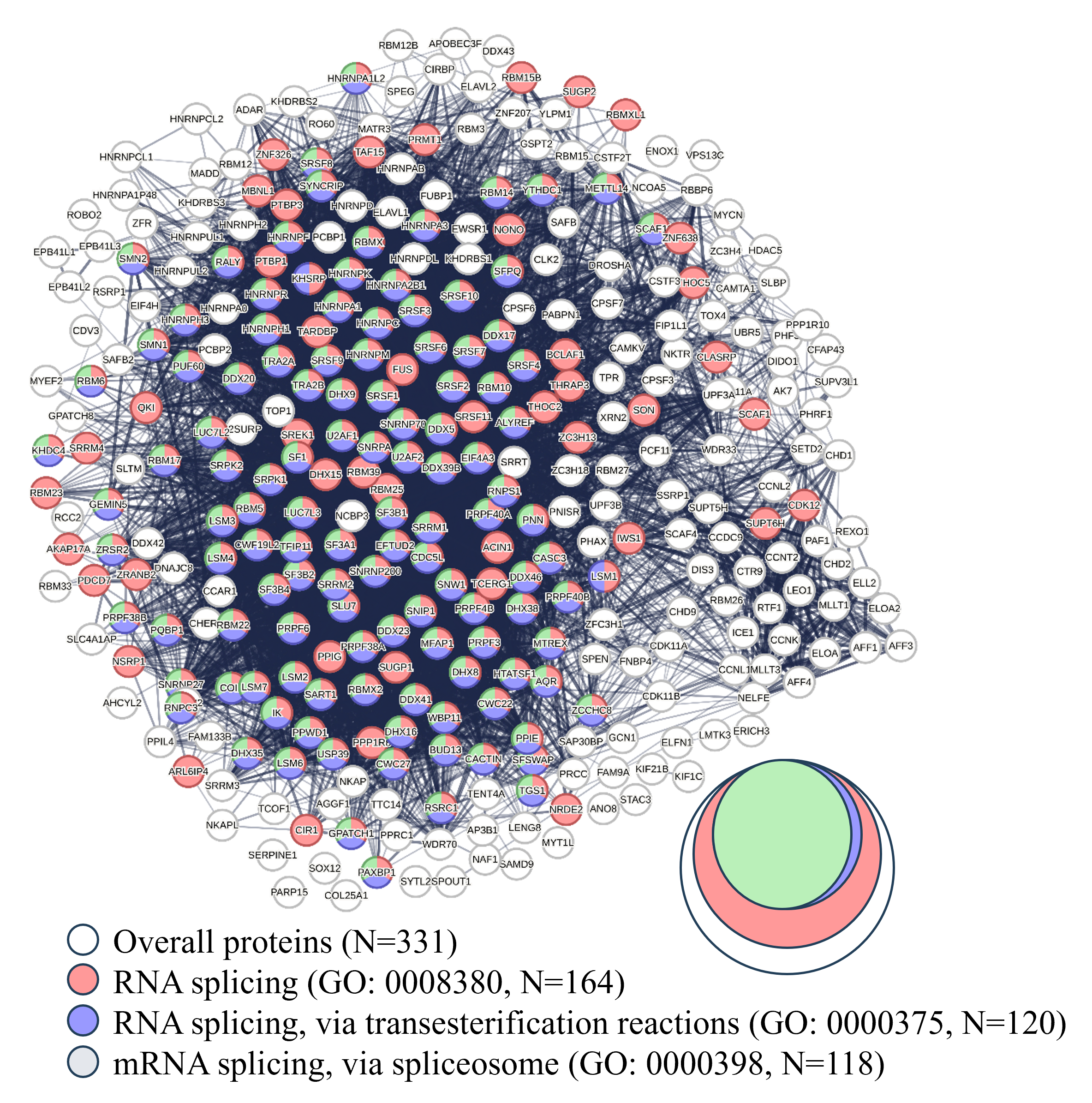


**Fig. S34.** **Visualization of the selected high-confidence Cluster 1- with RNA splicing (*i.e.,* GO: 0008380: RNA splicing; GO: 0000375: RNA splicing, via transesterification reactions; GO: 0000398: mRNA splicing, via spliceosome).** We visualized the PPI network for the high-confidence predicted proteins (with prediction probabilities ≥ 0.7) of Cluster 1- using the STRING website. In the STRING network, the edges between two proteins represent physical and functional protein associations and the network lines between two proteins indicate the strength of data support. We evaluated the potential biological processes by applying the GO enrichment biological processes significantly enriched (*p* value < 0.05) by proteins of the high-confidence RNA granule protein candidates in Cluster 1-.


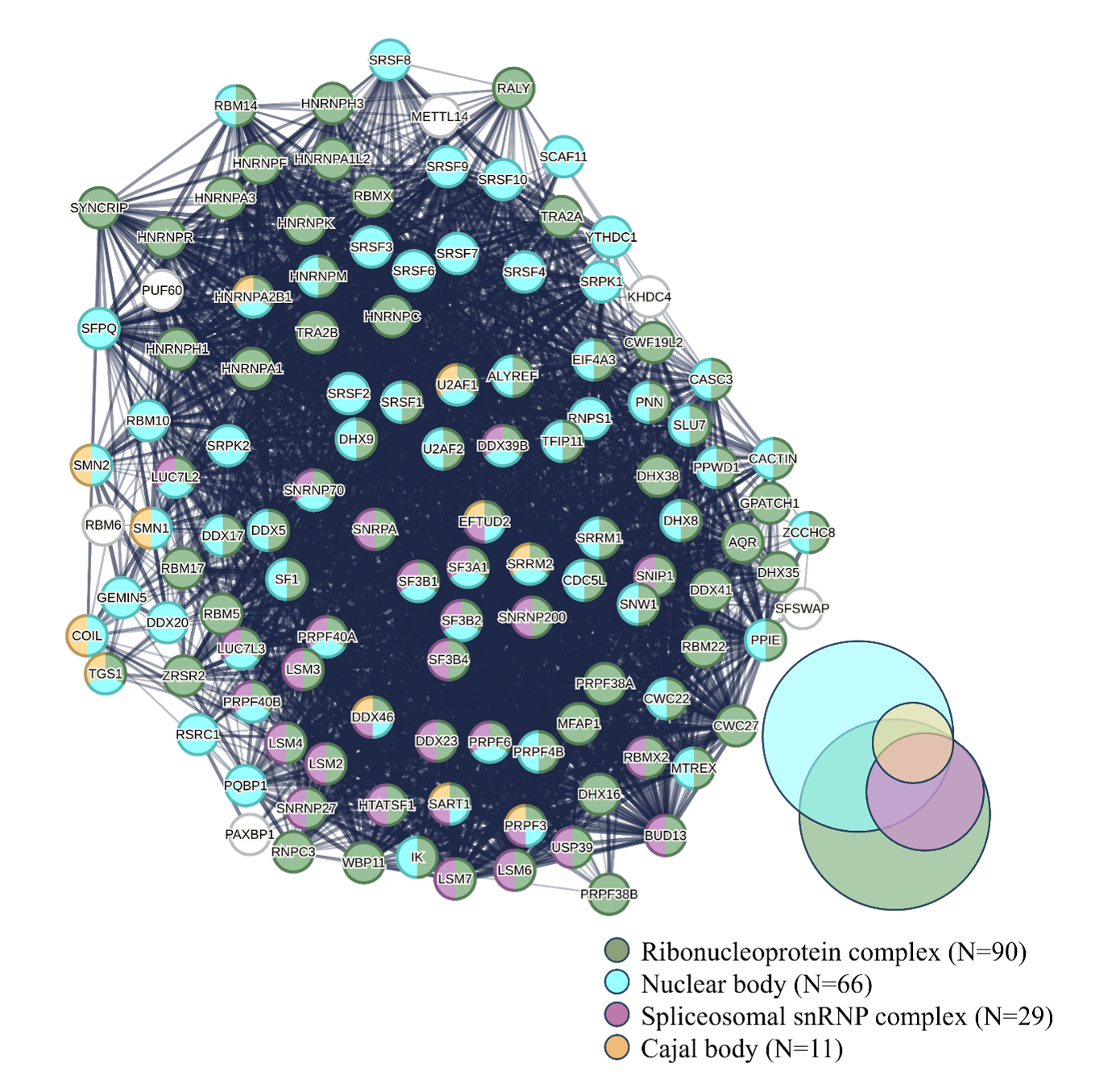


**Fig. S35.** **Visualization of the selected GOCC enrichment analysis on the selected proteins for the mRNA splicing, via spliceosome (GO: 0000398, *N* = 118) in Cluster 1- (as shown in Fig. S34).** We evaluated the PPI network of the selected proteins (*N* = 118) with the selected GOCCs significantly (*p* value < 0.05) enriched in Cluster 1- using the STRING website. In the STRING network, the edges between two proteins represent physical and functional protein associations, and the network lines between two proteins indicate the strength of data support.


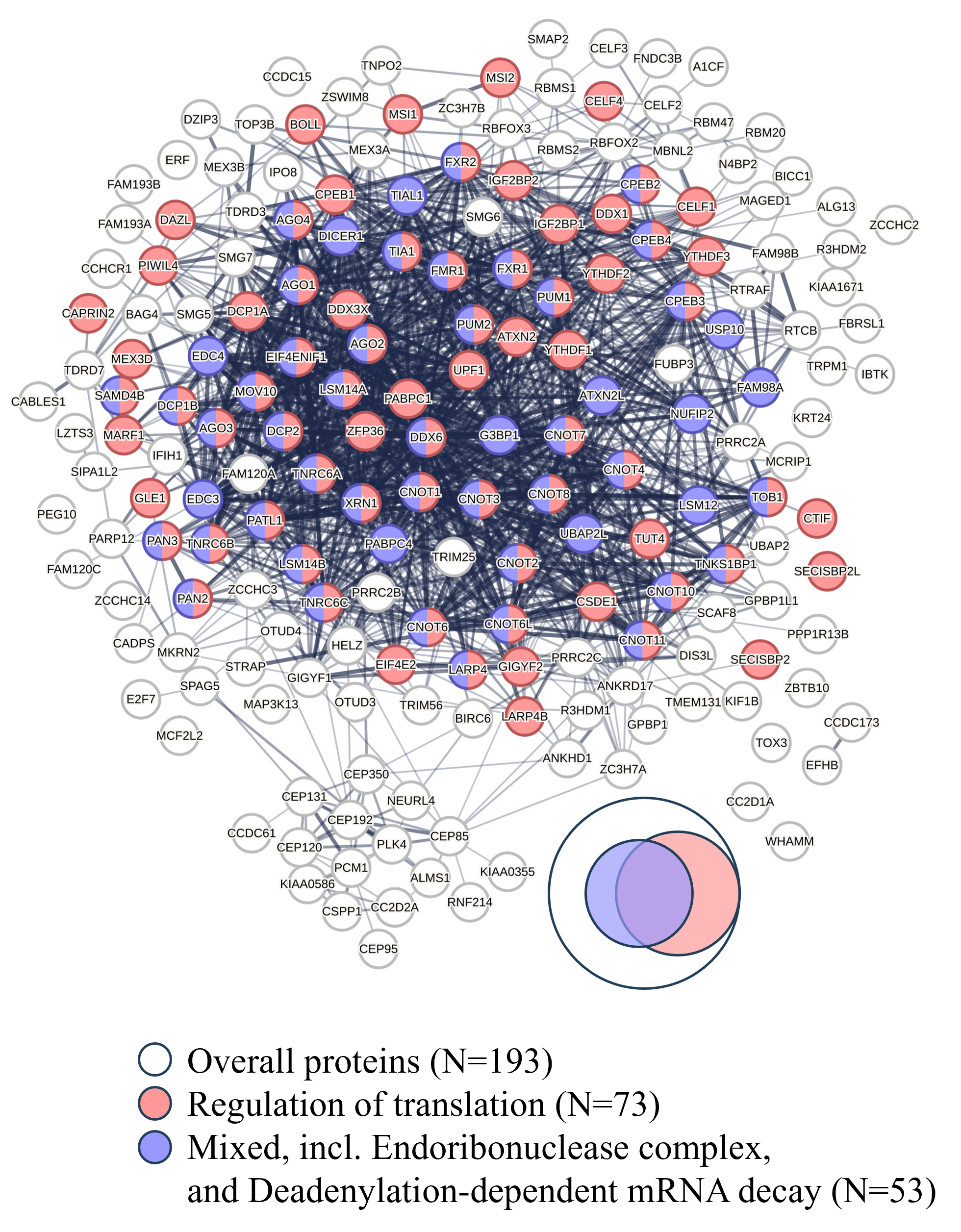


**Fig. S36.** **Visualization of the selected high-confidence Cluster 2- (proteins with prediction probabilities over 0.7, *N* = 193) with translation (GO: 0006417: Regulation of translation) and mRNA decay (CL: 2921: Mixed, incl. Endoribonuclease complex, and Deadenylation-dependent mRNA decay).** We visualized the high-confidence predicted proteins (with prediction probabilities ≥ 0.7) in Cluster 2- using the STRING website. In the STRING network, the edges between two proteins represent physical and functional protein associations, and the network lines between two proteins indicate the strength of data support. We evaluated the potential biological processes by applying the GOBPs and local network clusters significantly (*p* value < 0.05) enriched by high-confidence RNA granule proteins in Cluster 2-.


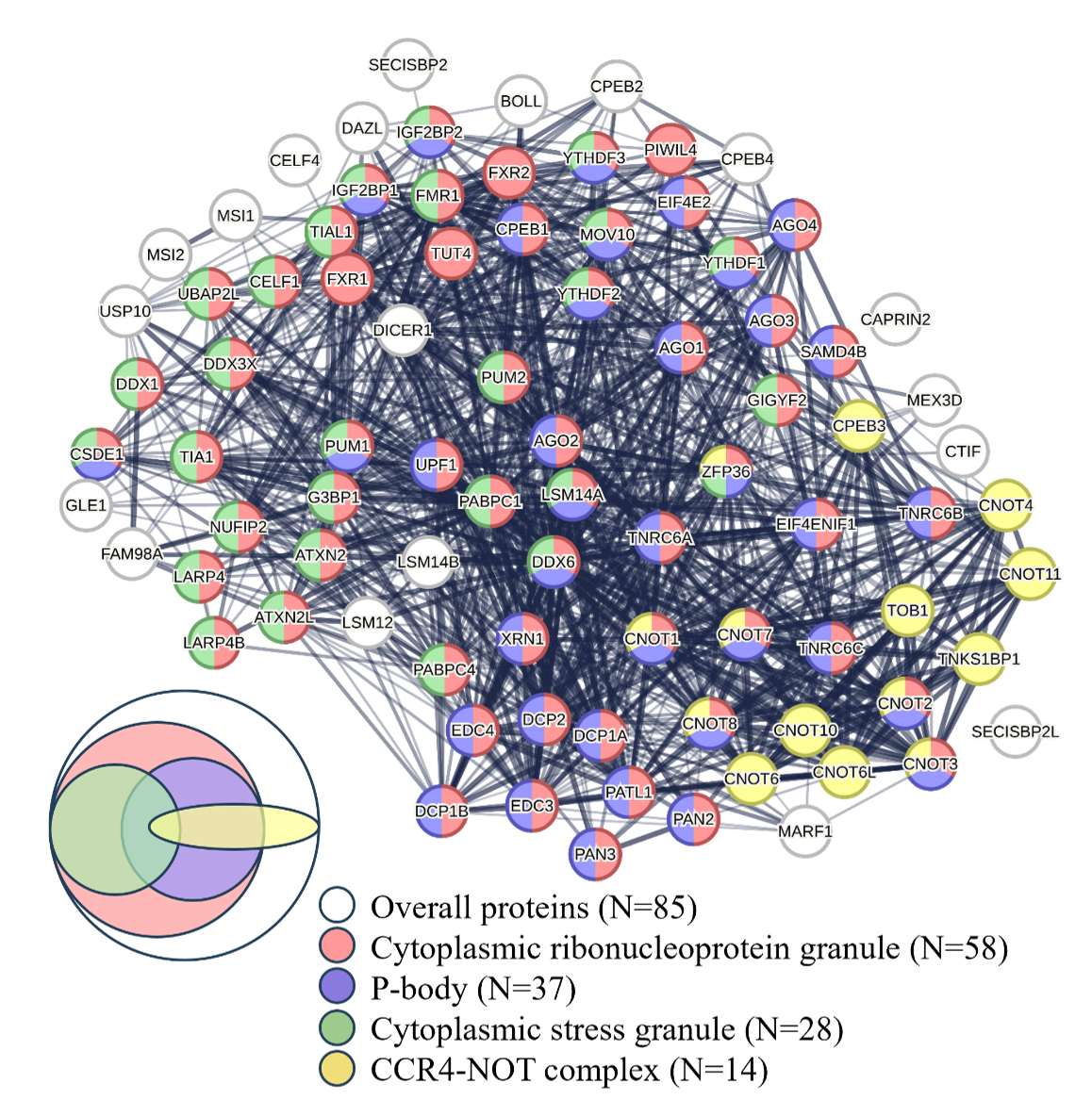


**Fig. S37.** **Visualization of the selected GOCCs enriched by the selected proteins of the Regulation of translation term (GO: 0006417, *N* = 73) and Mixed, incl. Endoribonuclease complex, and Deadenylation-dependent mRNA decay term (CL: 2921, *N* = 53) in Cluster 2-.** We visualized the PPI network for the potential cellular components significantly (*p* value < 0.05) enriched by the selected proteins in the terms of translation and mRNA decay in Cluster 2- using the STRING website. In the STRING network, the edges between two proteins represent physical and functional protein associations, and the network lines between two proteins indicate the strength of data support.


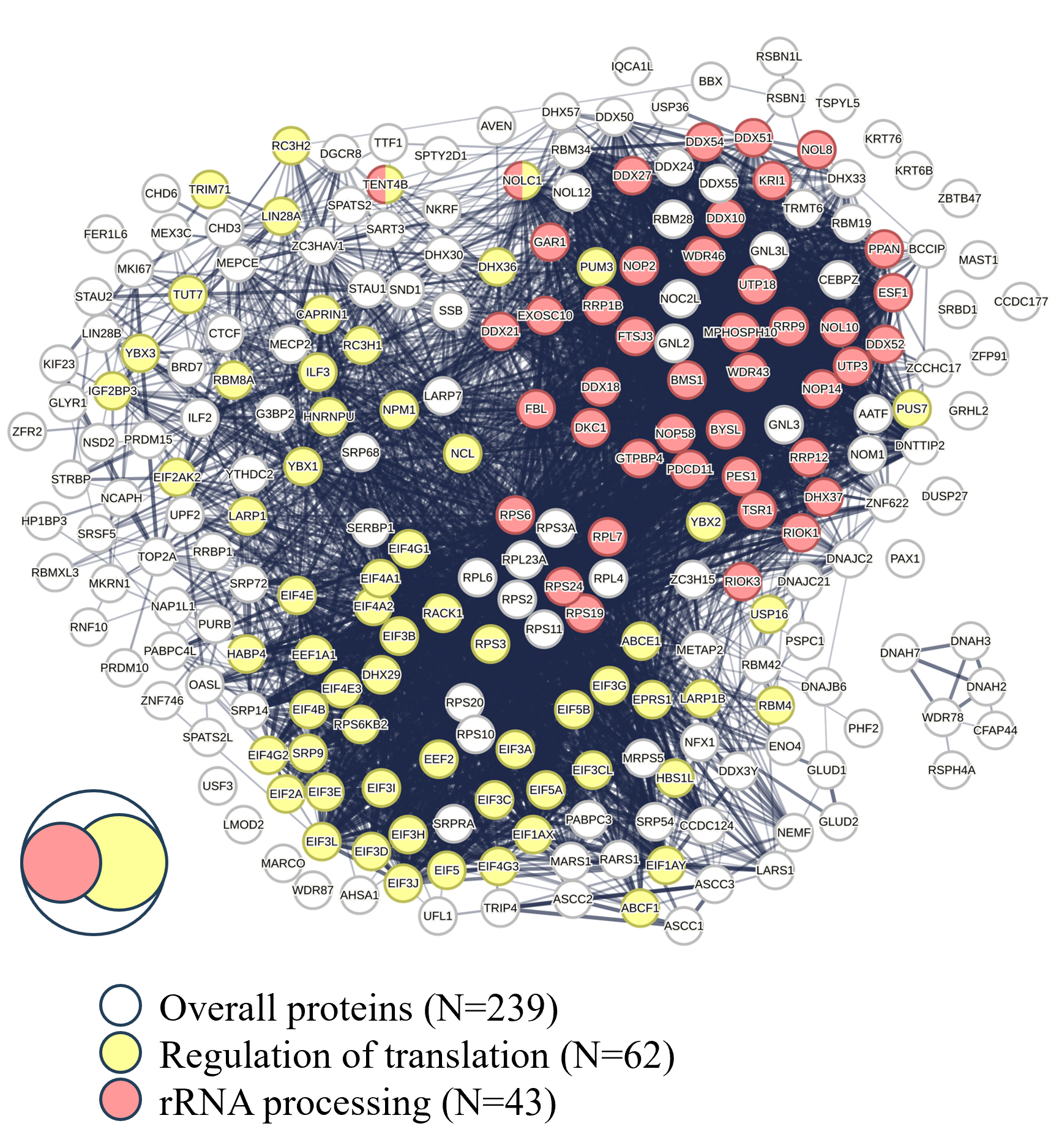


**Fig. S38.** **Visualization of the selected high-confidence Cluster 3- (proteins with prediction probabilities over 0.7, *N* = 239) with translation (GO: 0006417: Regulation of translation) and rRNA processing (GO: 0006364: rRNA processing).** We visualized the high-confidence predicted proteins (with prediction probabilities ≥ 0.7, *N* = 239) of Cluster 3- using the STRING website. In the STRING network, the edges between two proteins represent physical and functional protein associations, and the network lines between two proteins indicate the strength of data support. We evaluated the potential biological processes by applying the GOBPs and local network clusters significantly (*p* value < 0.05) enriched by proteins of the selected proteins.


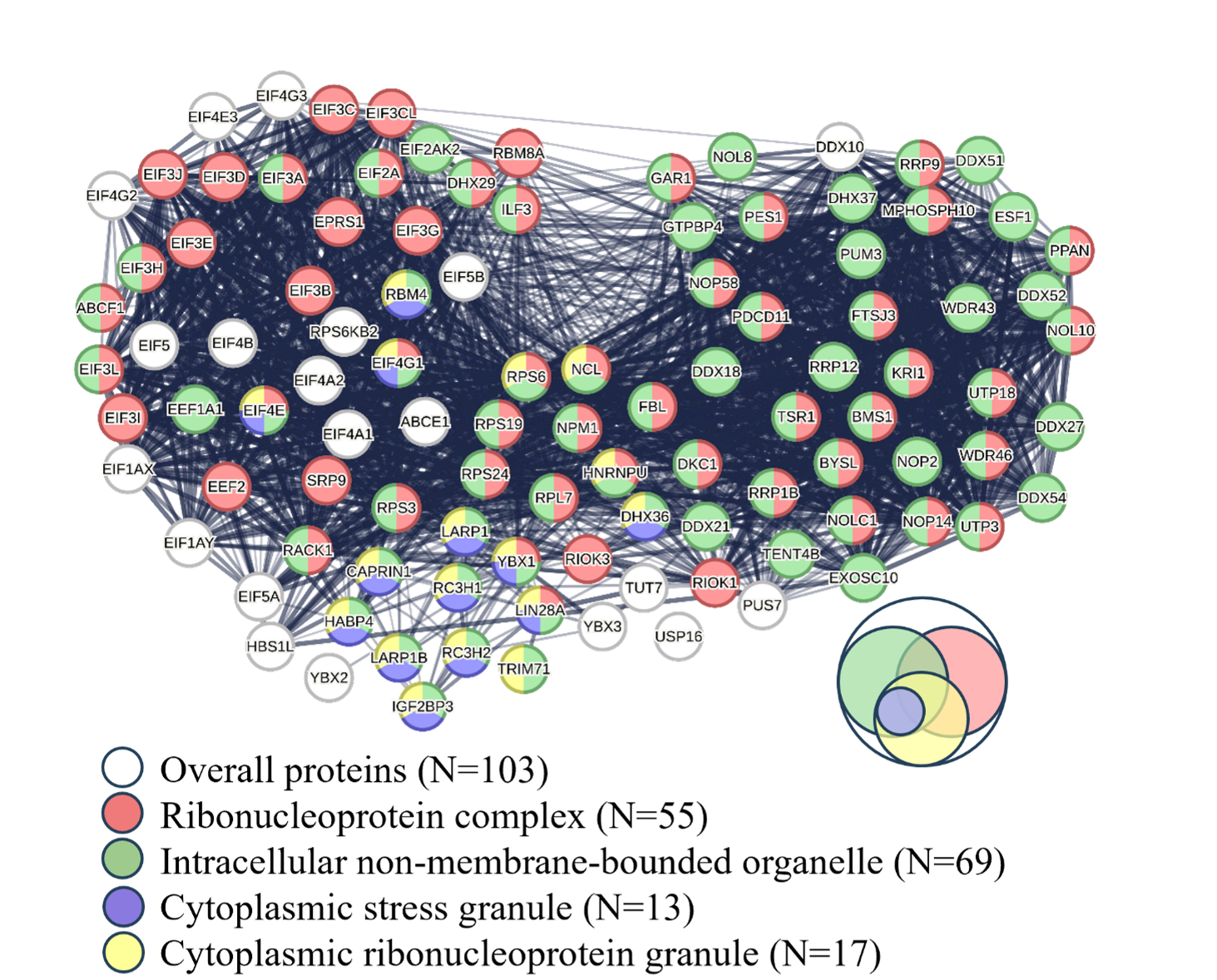


**Fig. S39.** **Visualization of the selected GO enrichment analysis on the selected proteins (*N* = 103) of the Regulation of translation (GO: 0006417, *N* = 62) and rRNA processing (GO: 0006364, *N* = 43) in Cluster 3-.** We evaluated the potential cellular components significantly (*p* value < 0.05) enriched by the selected proteins in Cluster 3- using the STRING website. In the STRING network, the edges between two proteins represent physical and functional protein associations, and the network lines between two proteins indicate the strength of data support.

**Table S1.** **Summary of human RNA granule proteins of different tiers collected from the RNAgranuleDB (access date July 2023).** SG_RBP_: total number of SG proteins of each tier in the RNAgranuleDB are identified as RBPs. PB_RBP_: total number of PB proteins of each tier in the RNAgranuleDB are identified as RBPs. PBSG_RBP_: total number of PBSG proteins of each tier in the RNAgranuleDB are identified as RBPs.

| **Standard** | **SG** | **PB** | **PBSG** | **SG_RBP_** | **PB_RBP_** | **PBSG_RBP_** |
| --- | --- | --- | --- | --- | --- | --- |
| Tier 1 | 280 | 61 | 473 | 264 | 55 | 426 |
| Tier 2 | 675 | 137 | 853 | 563 | 124 | 700 |
| Tier 3 | 399 | 189 | 854 | 288 | 82 | 501 |
| Tier 4 | - | - | 2548 | - | - | 2223 |
| Total Number | 1354 | 387 | 4728 | 1115 | 261 | 3850 |

**Table S2.** **Summary of distribution of different tier proteins collected from the RNAgranuleDB in the identified RNA granule proteome (*N*=6600) with different predicted probabilities by our RNA granule model.** N_overall_: total number of proteins in the prediction probability group. N_tier_1_ to N_tier_4_: the number of the prediction probability group in tier 1 to tier 4 proteins of the RNAgranuleDB. N_new_: the number of proteins in the prediction probability group not in the RNAgranuleDB.

| **Prediction Probability** | **N_overall_** | **N_tier_1_** | **N_tier_2_** | **N_tier_3_** | **N_tier_4_** | **N_new_** |
| --- | --- | --- | --- | --- | --- | --- |
| 0.9-1.0 | 194 | 138 | 18 | 7 | 24 | 7 |
| 0.8-0.9 | 626 | 207 | 78 | 48 | 130 | 163 |
| 0.7-0.8 | 1374 | 108 | 159 | 103 | 334 | 670 |
| 0.6-0.7 | 1944 | 14 | 161 | 137 | 435 | 1197 |
| 0.5-0.6 | 2462 | 1 | 147 | 154 | 485 | 1675 |

**Table S3.** **Summary of GO terms for human RNA granules and other biomolecular condensates collected from the QuickGO database (access date Oct. 2023).**

|  | **GO ID** | **GO Term** | **Total Number of Proteins** | **Number of Proteins with Predicted Probability ≥ 0.5** | **Percentage of Proteins with Predicted Probability ≥ 0.5** |
| --- | --- | --- | --- | --- | --- |
| **RNA Granules** | 0000932 | P-body | 100 | 74 | 74% |
|  | 0010494 | cytoplasmic stress granule | 89 | 80 | 90% |
|  | 0036464 | cytoplasmic ribonucleoprotein granule | 252 | 186 | 74% |
|  | 0043186 | P granule | 27 | 15 | 56% |
|  | 0033391 | chromatoid body | 13 | 11 | 85% |
|  | 0015030 | Cajal body | 60 | 41 | 68% |
|  | 0030496 | Midbody | 204 | 122 | 60% |
|  | 0016604 | nuclear body | 833 | 521 | 63% |
| **Other Biomolecular condensates** | 0014069 | postsynaptic density | 315 | 197 | 63% |
|  | 0005811 | lipid droplet | 105 | 35 | 33% |
|  | 0016605 | PML body | 109 | 67 | 61% |

| **Extracted clusters** | **N_overall_** | **N_tier_1_** | **N_tier_2_** | **N_tier_3_** | **N_tier_4_** | **N_new_** |
| --- | --- | --- | --- | --- | --- | --- |
| Cluster 1- | 331 | 88 | 67 | 26 | 101 | 49 |
| Cluster 2- | 193 | 142 | 13 | 7 | 5 | 26 |
| Cluster 3- | 239 | 104 | 45 | 14 | 47 | 29 |

**Table S4.** **Summary of distribution of different tier proteins collected from the RNAgranuleDB in the extracted high-confidence Cluster 1-, Cluster 2- and Cluster 3- (prediction propensities of proteins over 0.7).** N_overall_: total number of proteins in each selected high-confidence cluster. N_tier_1_ to N_tier_4_: the number of tier 1 to tier 4 proteins collected from the RNAgranuleDB in each selected high-confidence cluster. N_new_: the number of proteins of each selected high-confidence cluster not in the RNAgranuleDB.

**Table S5**. **Summary of distribution of popular proteins from the collected four published SG proteomes in selected clusters (prediction probabilities of proteins ≥ 0.7).**

| Num. Shared lists | Num. Proteins in the Cluster 1- (%) | Num. Proteins in the Cluster 2- (%) | Num. Proteins in the Cluster 3- (%) | Num. Proteins in All clusters (%) |
| --- | --- | --- | --- | --- |
| 4 (*N*=14) | 4 (29%) | 7 (50%) | 3 (21%) | 14 (100%) |
| 3 (*N*=70) | 12 (17%) | 39 (56%) | 16 (23%) | 67 (96%) |
| 2 (*N*=139) | 31 (22%) | 24 (17%) | 26 (19%) | 90 (65%) |
| 1 (*N*=588) | 52 (9%) | 38 (6%) | 36 (6%) | 174 (30%) |
| Overall (*N*=811) | 99 (12%) | 108 (13%) | 81 (10%) | 345 (43%) |
